# Thalamocortical bursts encode reward contingencies and drive associative learning

**DOI:** 10.1038/s41467-026-76581-6

**Published:** 2026-08-11

**Authors:** Filippo Heimburg, Nadin Mari Saluti, Lars-Lennart Oettl, Josephine Timm, Katharina Ziegler, Maria Helena Bortolozzo-Gleich, Thomas Kuner, Alexander Groh

**Affiliations:** 1 Medical Biophysics, Institute for Physiology and Pathophysiology, Heidelberg University, Heidelberg, Germany; 2 Institute for Anatomy and Cell Biology, Heidelberg University, Heidelberg, Germany; 3 Present Address: Institute for Experimental Epileptology and Cognition Research, University of Bonn, Bonn, Germany

**Keywords:** Learning and memory, Neural circuits, Cellular neuroscience, Ion channels in the nervous system

## Abstract

Learning requires adaptive changes in neuronal circuits, but how neurons encode learning content in their activity patterns to construct memories remains poorly understood. Using longitudinal multi-site recordings in freely moving male mice performing a sensory discrimination task, we discover the emergence of burst-coding neurons (BCNs) across cortical, thalamic, and extrathalamic regions. BCNs encoded task rules through the presence or absence of bursts, with their proportion increasing as learning progressed. Decoding analyses reveal that BCNs act as the principal carriers of rule information within the thalamocortical system. BCN burst rates scaled with stimulus valence, collapsed when contingencies were degraded, and inverted after repeated rule reversals, demonstrating that bursts dynamically track associative context during learning. Indeed, pharmacological and focal genetic suppression of thalamocortical bursting disrupted learning and task performance, establishing neuronal bursts as context-sensitive drivers of associative learning. These findings identify a burst-based neural code for stimulus–outcome associations in the thalamocortical system and provide causal evidence linking cellular firing dynamics to reward contingency learning.

## Abstract (web summary)

Researchers find that brief high-frequency bursts of neural firing in mouse thalamus and cortex track the value of sensory cues during learning and invert their stimulus selectivity in response to task rule inversions. They further show suppressing these bursts impairs learning.

## Introduction

Learning relies on plastic changes in neural circuits, but how such changes are initiated by neuronal firing patterns in vivo that signal when and where information should be stored remains unclear. Synaptic plasticity is highly sensitive to firing frequency and timing^1–7^, suggesting that specific firing motifs in vivo may instruct synapses to strengthen or weaken in order to store new information. Among neuronal firing motifs, high-frequency action-potential bursts stand out as stereotyped, syllable-like trains of rapid firing^8^ embedded within ongoing activity, raising the possibility that bursts act as dedicated cellular learning signals.

Neuronal burst firing is found across brain regions and species^8–10^. In the thalamocortical system, bursts have been extensively characterized mechanistically, from the ion-channel dynamics that generate bursting^10,11^ to dendritic input coupling across cortical layers^12^. Functionally, however, thalamocortical bursts are understood as mediators of sensory processing, perception or brain state^13–17^. Meanwhile, the relationship between thalamocortical bursts and learning remains unexplored. Recent simulations and mathematical analyses support the idea that bursts may act as learning signals to instruct synaptic plasticity without affecting sensory processing^18^, but direct experimental evidence linking thalamocortical bursts to behavioral learning has been lacking.

To address this gap, we employ a novel whisker-discrimination learning task^19^ in freely behaving mice, combining multi-site recordings of unit activity in four key regions of the whisker thalamocortical system with pharmacological and focal genetic interventions, to reveal that thalamocortical bursts contribute causally to learning and may therefore constitute a cellular mechanism involved in memory formation.

## Results

### Aperture discrimination learning during different task rules in mice

**Fig. 1:**
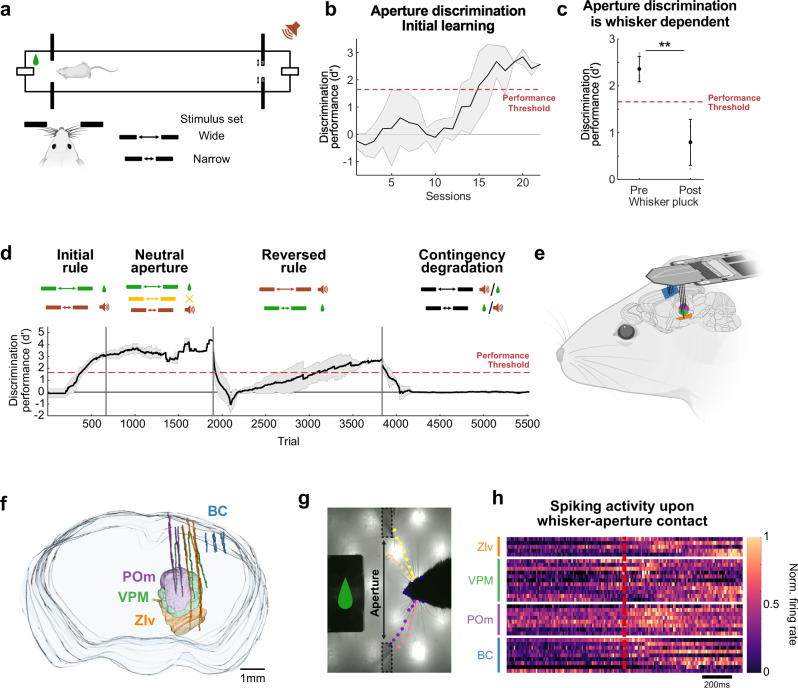
Whisker-dependent discrimination task to study neuronal dynamics during learning in freely moving mice. **a** Schematic of the whisker-dependent aperture discrimination paradigm, where the wide aperture is rewarded and the narrow aperture is punished. **b** Learning curve for the initial rule from six mice (average ± std). Expert-level discrimination performance (d’ ≥ 1.65, red dashed line) was achieved after 13.5 (±4.4) sessions. **c** Discrimination performance before (Pre) and after (Post) whisker removal (*p* = 0.0011, two-tailed paired *t*-test). **d** Performance across learning stages: initial learning, neutral aperture, reversed rule, and degradation. Each stage’s rules are indicated at the top, with average trial performance (black line) and standard deviation (shaded area). **e** Schematic of multi-site tetrode recordings: 16 tetrodes were implanted in barrel cortex (BC, 5), ventral posteromedial nucleus (VPM, 4), posterior medial nucleus of the thalamus (POm, 4), and zona incerta ventralis (ZIv, 3). **f** Example tetrode track reconstruction from post-mortem histology. **g** High-speed camera frame showing tracked whiskers (colored dotted lines) via DeepLabCut (20) to mark whisker-aperture touch events. **h** Example spiking activity from BC, VPM, POm, and ZI in response to whisker-aperture touches (red line), normalized from 0 to 1. **: *p* ≤ 0.01; **c:** two-tailed paired *t*-test, *n* = 6 mice (comparing the session before with the session after whisker pluck). For detailed description of statistics see Supplementary Data 1. Source data are provided as a Source data file.

In this novel go/no-go operant learning task^19^ we trained mice to associate apertures of different widths with a respective reward or punishment (Fig. 1a). Food-controlled mice were freely roaming on a linear platform with one lick-port at each end of the platform. To reach the lick-ports, mice had to pass through two-winged motorized apertures, which they touch with their whiskers. Licking was registered at the lick-ports and triggered either a reward (condensed milk) or a punishment (white noise, 120 dB, random duration between 1 and 3 s). In the initial rule, we trained mice to associate the wide aperture with reward and the narrow aperture with punishment. Mice learned to discriminate between the two apertures (d’ above 1.65) after an average of 13.5 sessions (±4.4 SD, *n* = 6 mice) or 388 trials (± 53 SD, *n* = 6), corresponding to seven days of training (Fig. 1b, Supplementary Fig. 1 and Supplementary Movies 1–3). We validated the whisker-dependency of the task by removing their whiskers (comparing the sessions before and after whisker removal with a performance of 2.36 ± 0.27 SD and 0.79 ± 0.49 SD, respectively, *p*-value 0.0011, two-sided paired *t*-test, *n* = 6) (Fig. 1c) and by numbing their whisker pads with a topical anesthetic (lidocaine cream)^19^, both of which abolished task performance. We trained mice in four consecutive training stages with distinct stimulus-outcome contingencies (Fig. 1d). The introduction of a third aperture, which provided neither reward nor punishment (henceforth referred to as the “neutral aperture”), did not disrupt overall task performance (Fig. 1d). Introducing a rule reversal abolished performance and led to subsequent re-learning of the reversed rule, albeit over a significantly extended period (1077 ± 330 trials, *p*-value compared to initial learning speed 0.03125, Wilcoxon signed rank test, *n* = 6, Supplementary Fig. 1). Degrading the aperture-outcome contingencies by randomizing reward and punishment presentations resulted in a complete loss of discrimination ability, yielding an average performance of d prime of −0.02 ( ± 0.20 SD, *n* = 40 sessions). The ability to train mice in multiple rule settings in this paradigm allowed us in the following to examine how stimulus-outcome associations are encoded in the brain to drive learning.

### Burst-coding neurons (BCNs) encode the aperture width through the presence or absence of bursts

We investigated four key brain regions implicated in somatosensory processing by recording multi-site unit activity (Fig. 1e) in the barrel cortex (BC), the ventral posteromedial nucleus (VPM), the posterior medial nucleus of the thalamus (POm), and in the zona incerta ventralis (ZIv). Post-mortem tetrode track reconstructions validated tetrode localization (Fig. 1f), and only data from correctly placed tetrodes were included in the analysis. The time points of the whisker-aperture interactions were extracted from the high-speed videos using DeepLabCut^20^ (Fig. 1g, h). In the following, we present the results from a cohort of six mice that went through the four training stages (Fig. 1d) while their unit activity was recorded in BC, VPM, POm, and ZIv. We split units according to waveform analysis into regular- (RS) and fast-spiking (FS) units (Supplementary Fig. 2). We restricted the following analyses to RS units in BC, VPM, and POm and RS/FS units in ZIv, unless otherwise stated.

**Fig. 2:**
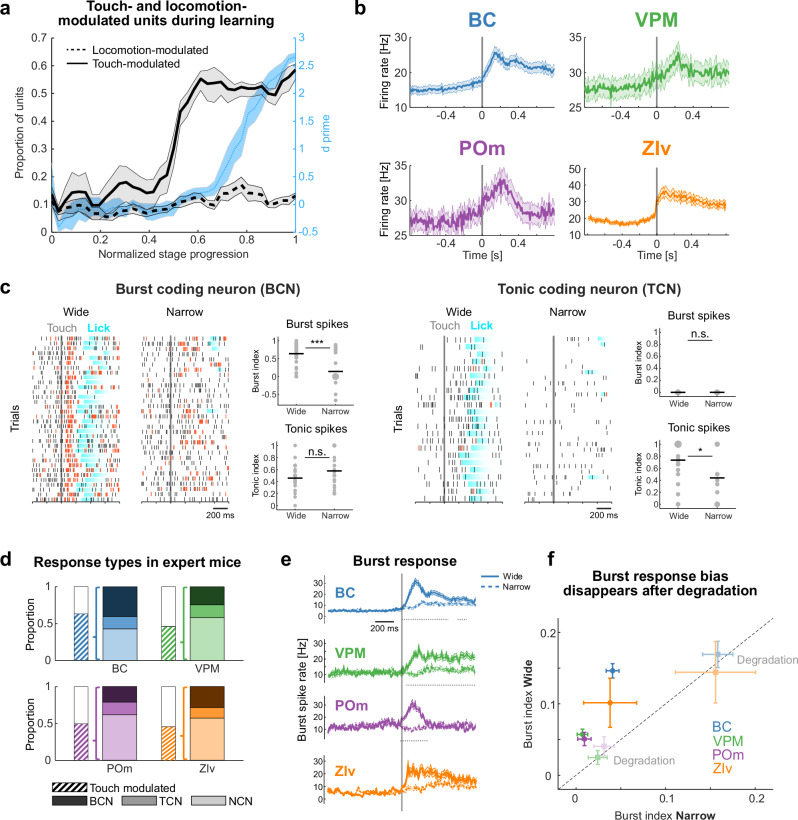
Burst encoding of aperture width during task performance. **a** Proportion of whisker-touch- (solid line) and locomotion-modulated (dashed line) units across learning (mean ± SEM). Whisker-touch modulation is defined by significant responses to the apertures (two-sided Wilcoxon signed rank test, based on unit firing rates), and locomotion-modulated units were identified by stepwise multiple linear regression. Discrimination performance (d′) is indicated with a blue line. **b** Mean population firing rate (shaded areas show SEM) in response to aperture whisker-touches in expert sessions (d′ ≥ 1.65). **c** Spike rasters for example BCN (left) and TCN (right). Burst spikes are marked in red, lick events in blue. Burst and tonic indices were calculated for each wide and narrow trial: *n* = 34 wide trials and *n* = 32 narrow trials for the example BCN, and *n* = 23 wide trials and *n* = 30 narrow trials for the example TCN. BCNs and TCNs were defined by significant differences in their burst or tonic indices in response to the different apertures (two-tailed Wilcoxon signed-rank test; for the example BCN, *p* = 2.3492 × 10⁻⁶ for the burst index and *p* = 0.1210 for the tonic index; for the example TCN, *p* = NaN for the burst index and *p* = 0.0242 for the tonic index), shown in adjacent panels for one exemplary BCN and TCN. **d** Proportion of response types (BCNs, TCNs, and NCNs) in expert mice across regions (for exact values see Supplementary Data 1). **e** Burst firing rates of BCNs in response to wide (solid) and narrow (dashed) apertures (mean ± SEM). Significant differences (α = 0.05) are indicated with dotted lines. **f** Mean burst indices per region, split between wide and narrow apertures, with SEM as error bars. Dotted lines indicate equal burstiness for wide and narrow apertures. The burst bias in expert sessions (circles) disappears during the degradation stage (pale squares). n.s.: *p* > 0.05, *: *p* ≤ 0.05, **: *p* ≤ 0.01 and ***: *p* ≤ 0.001; **c** two-tailed Wilcoxon signed rank test, **e**: two-tailed Welch’s *t*-test with Benjamini–Hochberg correction for multiple comparisons. For detailed description of statistics see Supplementary Data 1. **a**, **b**, **d**–**f** data pooled from *n* = 6 mice. Source data are provided as a Source data file.

First, we quantified the proportion of units responsive to whisker touch and locomotion across all recording sites (see Methods). We observed a substantial increase in the proportion of whisker touch-responsive units, from 12.5% (±3.2%) in the initial third to 52.2% (±2.5%) in the final third of the learning phase. In contrast, the proportion of locomotion-encoding units remained relatively constant throughout learning at around 10% (Fig. 2a). Looking at individual recording sites, we found robust whisker touch responses in each of the four recorded regions (Fig. 2b). Whisker-touch responses consisted of a mix of both tonic and burst spikes in all four regions. Baseline burst fractions varied among the regions and peaked shortly after touch onset (Supplementary Fig. 3a).

We then asked if the aperture width is encoded in the spiking response profiles. By inspecting response profiles of individual units when mice encountered either the wide or narrow aperture, we identified a subset of units which exhibited higher bursting activity for the wide aperture compared to narrow aperture (Fig. 2c, left). We defined these units, which encode the aperture state through the presence or absence of bursts, as burst-coding neurons (BCNs). A different subset of units also encoded the aperture state, but only through tonic spiking (Fig. 2c, right), henceforth defined as tonic-coding neurons (TCNs). The remainder of touch-responsive units did not respond differently to the wide and narrow aperture, and these are defined as non-coding neurons (NCNs). For exact definitions of BCNs, TCNs, and NCNs, see Methods “Neural response metrics” section. Notably, we found that these three response types (BCNs, TCNs, NCNs) are present in all four recorded brain regions, with BCNs being the largest subset among the aperture coding units in expert animals (Fig. 2d).

To determine whether the prevalence of these coding types depends on firing-rate statistics alone, we generated session-specific surrogate spike trains that preserved per-neuron spike counts within baseline and response windows while randomizing spike timing across neurons. This approach effectively disrupts burst-specific spike patterns while maintaining the overall firing rate dynamics. Comparing observed to surrogate counts using Monte-Carlo statistics demonstrated a strong enrichment of BCNs (Fisher combined *p* = 3.95 × 10⁻²⁴, *χ*² = 233.21, df = 54), while TCNs did not differ from chance levels (*p* = 0.070). Consistently, z-statistics comparing observed to surrogate counts of BCNs and TCNs per session showed a strong positive shift for BCNs (mean *z* = 2.91) but remained near zero for TCNs (mean *z* = 0.67), indicating that burst-based coding cannot be explained by firing-rate alone (Supplementary Fig. 3d).

When we compared the burst-firing rate of BCNs between the wide and narrow apertures in a time-resolved manner the burst-coding of the aperture state became even more apparent. Across all recorded brain regions, wide-aperture-contacts consistently elicited higher burst-firing rates than narrow-aperture-contacts in BCNs but not in TCNs (Fig. 2e for BCNs, Supplementary Fig. 3b for TCNs). Notably, the POm exhibited only transient activation upon aperture-contact (Fig. 2e), while other brain areas showed prolonged activation after aperture-contact.

To further resolve how this population bias arises at the single-unit level, we examined the joint distribution of burst indices for wide and narrow apertures (Supplementary Fig. 3c). BCNs were predominantly associated with enhanced bursting during wide-aperture sampling, whereas responses to narrow apertures were split between weak suppression and enhancement. In total, 92.11% of BCNs exhibited burst enhancement during wide-aperture trials, while narrow-aperture responses were divided between suppression (46.84%) and weak enhancement (53.16%). Only a small subset of neurons showed the opposite coding motif, favoring the narrow aperture. These coding motifs indicate that the population bias toward the wide aperture is driven primarily by selective burst enhancement during wide aperture sampling, rather than by generalized suppression during narrow aperture sampling.

Finally, we tested whether burst-coding is maintained after degrading the contingencies between stimulus and outcome during a degradation stage during which the aperture states and outcomes were randomized in each trial (50% chance for “wide” or “narrow” and reward or punishment, respectively) (Fig. 1d). We found that burst-coding disappeared following the degradation stage (Fig. 2f).

### BCNs track stimulus-outcome associations even after multiple rule reversals by inverting burst-encoding of the physical stimuli

**Fig. 3:**
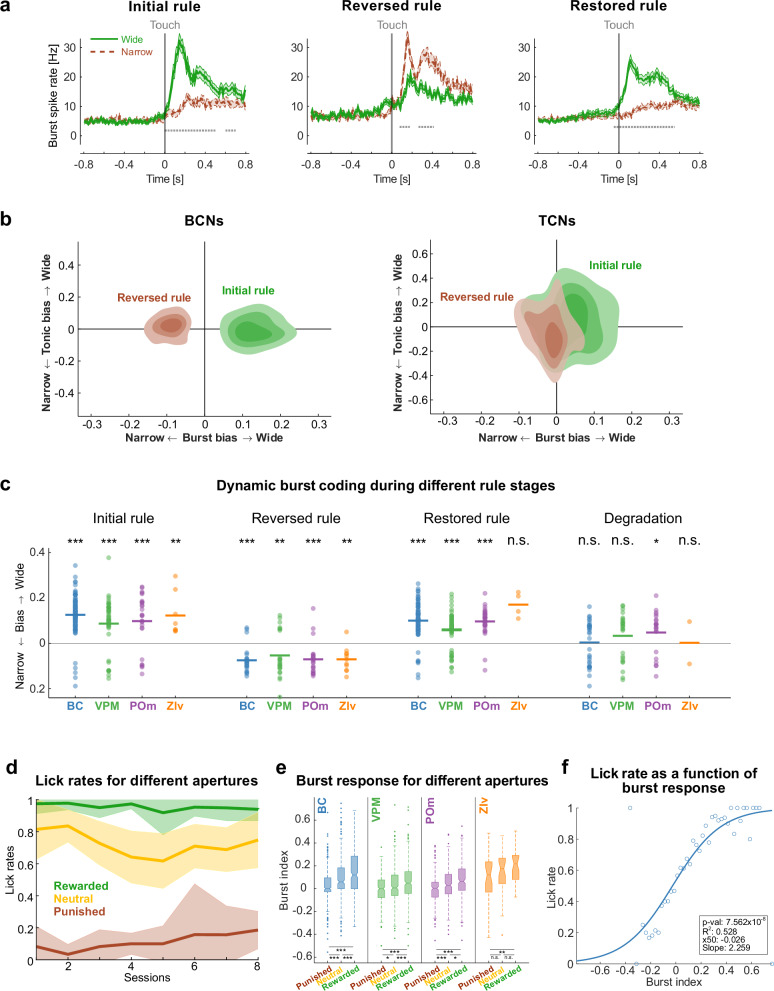
BCNs track stimulus-outcome associations even after multiple rule-reversals by inverting burst-encoding of the physical stimuli. **a** Burst firing rates of BCNs in BC in response to wide (green) and narrow (maroon) aperture (represented as mean ± SEM, *n* = 6 mice). Statistically significant differences (*α* = 0.05) are indicated with dotted lines. For VPM, POm, ZIv, see Supplementary Fig. 4. **b** Distribution of burst and tonic bias (see Methods for details) of BCNs (left) and TCNs (right), pooled from BC, VPM, POm, and ZIv (*n* = 6 mice). The initial rule is shown in green and the reversed rule in maroon. Contour lines correspond to the first (Q1), median (Q2), and third quartile (Q3) of units in the scatter plot. For individual regions, see Supplementary Fig. 5. **c** Burst bias of individual BCNs across the initial rule, reversed rule, restored rule, and degradation stage in BC, VPM, POm, and ZIv (*n* = 6 mice). Exact *p*-values were as follows: BC, *p* = 7.7833 × 10⁻¹⁶ initial rule, *p* = 2.6180 × 10⁻⁵ reversed rule, *p* = 2.2040× 10⁻¹² restored rule, and *p* = 0.9702 degradation; VPM, *p* = 4.0875 × 10⁻⁵ initial rule, *p* = 0.0093 reversed rule, *p* = 3.0897 × 10⁻⁶ restored rule, and *p* = 0.0865 degradation; POm, *p* = 9.1496 × 10⁻⁴ initial rule, *p* = 5.4514 × 10⁻⁴ reversed rule, *p* = 1.9330 × 10⁻⁶ restored rule, and *p* = 0.0424 degradation; ZIv, *p* = 0.0078 initial rule, *p* = 0.0068 reversed rule, *p* = 0.1250 restored rule, and *p* = 1.0000 degradation. **d** Mean lick rates (STD as shaded regions) across sessions of the neutral aperture stage. The three different apertures are represented separately (punished in red, neutral in yellow, and rewarded in green). **e** Box plots comparing the burst indices in response to the different aperture types for each region (BC, VPM, POm, and ZIv). Box plots show the median, with box limits indicating the 25th and 75th percentiles. Median [Q1, Q3] values were as follows: BC units: narrow, 0 [−0.0268, 0.0952]; intermediate, 0.0591 [0, 0.1829]; wide, 0.1193 [0, 0.2843]. VPM units: narrow, 0 [−0.0780, 0.0769]; intermediate, 0.0072 [−0.0603, 0.1193]; wide, 0.0490 [−0.0412, 0.1492]. POm units: narrow, 0.0029 [−0.0735, 0.0561]; intermediate, 0.0299 [−0.0448, 0.1256]; wide, 0.0651 [−0.0145, 0.1733]. ZIv units: narrow, 0.1205 [−0.0299, 0.2353]; intermediate, 0.1746 [0.0435, 0.2664]; wide, 0.2178 [0.0812, 0.2913]. Exact *p*-values were as follows: BC, *p* = 4.6968 × 10⁻⁷ for narrow vs. intermediate, *p* = 9.0064 × 10⁻¹⁸ for narrow vs. wide, and *p* = 1.9799 × 10⁻⁹ for intermediate vs wide; VPM, *p* = 0.0160 for narrow vs. intermediate, *p* = 1.2633 × 10⁻⁶ for narrow vs. wide, and *p* = 5.4900 × 10⁻⁴ for intermediate vs wide; POm, *p* = 4.0669 × 10⁻⁴ for narrow vs. intermediate, *p* = 3.6910 × 10⁻⁷ for narrow vs. wide, and *p* = 0.0113 for intermediate vs wide; ZIv, *p* = 0.1631 for narrow vs intermediate, *p* = 0.0086 for narrow vs. wide, and *p* = 0.3147 for intermediate vs. wide. **f** Lick rate as a function of burst bias in BC, considering go, no-go, as well as neutral trials. Properties of the logistic regression: *R*^2^: 0.528, *p*: 7.562e-08, inflection point (x50): −0.026, slope: 2.259. n.s.: *p* > 0.05, *: *p* ≤ 0.05, **: *p* ≤ 0.01 and ***: *p* ≤ 0.001; **a**: two-tailed Welch’s t-test with Benjamini–Hochberg correction for multiple comparisons, **c**, **e**: two-tailed Wilcoxon signed rank test (*p*-values were Bonferroni corrected for multiple testing), **f**: *F*-test comparing the sigmoid fit against a constant mean model. For detailed description of statistics see Supplementary Data 1. Source data are provided as a Source data file.

An important consideration for burst coding is whether BCNs encode the aperture’s physical properties (width) or their contextual parameters (outcome). We surmised that a mere encoding of the physical parameters would require the BCNs to retain their code (wide = burst, narrow = no burst), when the aperture context is changed during the reversed rule stage (Fig. 1d). We observed the opposite in that BCNs inverted their burst encoding of the physical stimuli when comparing narrow/wide encoding in expert sessions of the initial and reversed rule (Fig. 3a shows BC units, for VPM, POm, ZIv, see Supplementary Fig. 4). To explore how flexible the burst-code inversion is, we trained mice in an additional rule reversal stage following the reversed rule stage, where we restored the initial rule set (wide = rewarded, narrow = punished). After relearning, BCNs had again inverted their burst encoding back to the initial burst code (wide = burst, narrow = no burst). These results demonstrate that burst coding is not primarily determined by the physical properties of the stimuli, but rather by the association between the stimulus and the outcome, and that BCN-bursts track the rewarded stimulus through multiple rule-reversals by inverting burst-encoding.

We explored the burst-code inversion in BCNs and TCNs in more detail by calculating burst- and tonic biases, which measure the occurrence of burst- and tonic spikes upon whisker touches of the wide or narrow aperture (see Methods). This revealed that (1) BCNs employ burst coding and not tonic coding and that (2) BCNs undergo a more pronounced code-inversion after rule reversal compared to TCNs, which remained relatively stable (Fig. 3b shows units pooled from all regions; see Supplementary Fig. 5 for the individual brain regions).

A comparative longitudinal analysis of BCNs in BC, VPM, POm, and ZIv over the course of the different rule stages revealed that BCNs dynamically track the rewarded aperture with learning, independently of whether the rewarded aperture was wide or narrow. During degradation, this bias toward the rewarded aperture was markedly reduced. This reduction resulted both from an actual decrease in burst bias in individual BCNs and from a redistribution at the population level, in which narrow- and wide-encoding BCNs occurred in more similar proportions (Fig. 3c). Only the POm retains significant burst coding, possibly due to the brevity of the degradation period.

### Burst coding scales with stimulus valence and predicts licking behavior

In the next training stage, we introduced a third aperture that was intermediate between the narrow and wide aperture and was neither rewarded nor punished (hence referred to as “neutral”). We observed that the lick rates for the intermediate aperture fell between those of the rewarded and punished apertures, indicating a possible scaling of lick behavior with stimulus valence (Fig. 3d). Similarly, the burst firing responses to the neutral aperture significantly differed from the others and was positioned between those of the rewarded and punished apertures in all four recording regions (Fig. 3e), supporting the notion that burstiness scales with stimulus valence.

Furthermore, we explored whether the mouse’s decision to lick in a given trial is predicted by the strength of the burst coding during aperture sampling. Notably, the likelihood of a lick event (calculated as the proportion of trials with a lick outcome for a given burst bias) increased as a function of the burst bias (Fig. 3f), as captured by the logistic fit, which significantly predicts lick rate with good accuracy (*R*² = 0.528, *p* = 7.562e-08). The inflection point of the fit, occurring near zero burst bias (x50 = -0.026), suggests that modulation of bursting (either suppression or enhancement) could serve as an effective neural signal to inhibit or drive reward-seeking behavior. This underscores the close relationship between burst coding and behavioral outcomes.

### Burst coding emerges with learning and is correlated with task performance

**Fig. 4:**
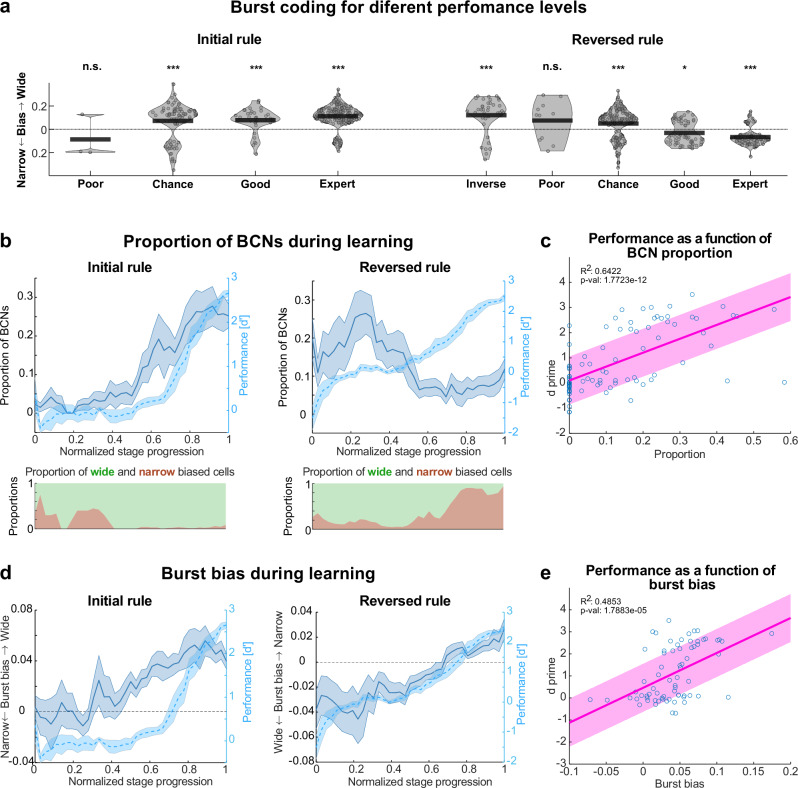
Burst encoding emerges with learning. **a** Violin plots of burst bias in pooled BCNs across BC, VPM, POm, and ZIv regions, shown for different performance levels and learning stages. Performance levels are defined as inverse (d’ ≤ −1.65), poor (−1.65 <d’ < −0.5), chance (−0.5 ≤ d’ ≤ 0.5), good (0.5 <d’ <1.65), and expert (d’ ≥ 1.65). Exact *p*-values were as follows: initial rule, *p* = 0.5000 for poor, *p* = 6.9403 × 10⁻⁴ for chance, *p* = 3.5984 × 10⁻⁴ for good, and *p* = 5.4861 × 10⁻²³ for expert; reversed rule, *p* = 3.3155 × 10⁻⁴ for inverse, *p* = 0.1748 for poor, *p* = 2.0905 × 10⁻⁶ for chance, *p* = 0.0113 for good, and *p* = 3.5552 × 10⁻¹⁰ for expert. **b** Line graphs showing the average proportion of BCNs in BC (shaded SEM) as learning progresses, with mean performance (d′) indicated by a dotted line. Right panel shows data under the reversed rule, with proportions of wide- and narrow-biased BCNs below. **c** Positive correlation between performance and BCN proportion (*R*^2^: 0.6422, *p*: 1.7723e-12, shaded area as STD of residuals). **d** Average burst bias in BC across animals over learning (shaded SEM), with bias between narrow and wide apertures shown for the initial rule (left) and reversed rule (right). Average d′ is indicated by the dotted line. **e** Session performance correlates with the average burst bias per session (*R*^2^: 0.4853, *p*: 1.7883e-05, shaded area as STD of residuals). For VPM, POm, ZIv, see Supplementary Fig. 7 and 8. n.s.: *p* > 0.05, *: *p* ≤ 0.05, **: *p* ≤ 0.01 and ***: *p* ≤ 0.001; **a** two-tailed Wilcoxon signed rank test, **c**, **e** Pearson-correlation *p*-value based on a two-sided *t*-test. For detailed description of statistics see Supplementary Data 1. Data pooled from *n* = 6 mice. Source data are provided as a Source data file.

We next investigated the relationship between task proficiency and burst coding by grouping the sessions of the six mice into performance categories and computing the strength and direction of aperture burst coding for each category. As performance improved, a burst bias towards the rewarded aperture emerged, supporting a direct link between task proficiency and burst coding (Fig. 4a shows units pooled from all regions, see Supplementary Fig. 6 for individual brain regions). Upon rule reversal, the burst bias was initially inverted, concomitantly with inverted performance (negative d’), reflecting a carryover from prior performance under the initial rules. With increased proficiency in the reversed rule stage, the burst bias shifted to favor the – now-rewarded – narrow aperture (Fig. 4a).

We next asked whether burst coding emerges during learning, by calculating the proportion of BCNs and their burst bias as a function of learning progression. In the initial learning stage, the proportion of BCNs in the BC increased in parallel with improving performance (Fig. 4b, left). After rule reversal, this proportion gradually declined, primarily attributed to a reduction in BCNs coding for the wide aperture, in favor of those coding for the narrow aperture (Fig. 4b, right). Notably, the proportion of BCNs shows a strong positive correlation with performance (*R*²: 0.6422, *p*: 1.7723e-12), highlighting the critical role of BCNs during learning and performance optimization (Fig. 4c). Similarly, the average burst bias in BC across animals evolved alongside learning, progressively favoring the rewarded aperture (Fig. 4d, left). Notably, during the rule reversal, we observed an inversion in the average burst bias, reflecting a shift in neural burst coding patterns (Fig. 4d, right). The average performance per session was positively correlated with the burst bias exhibited within that session (*R*²: 0.4853, *p*: 1.7883e-05), indicating that the strength of burst coding is closely linked to the progression of learning (Fig. 4e). Similar results were observed in all four recorded regions (for VPM, POm, ZIv, see Supplementary Figs. 7 and 8).

### BCNs predict aperture states better than other touch-modulated units

**Fig. 5:**
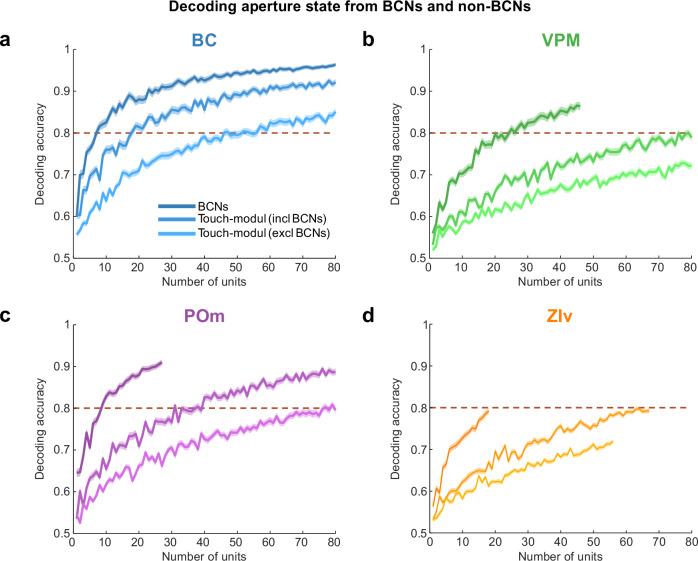
BCNs predict aperture states better than other touch-modulated units. **a**–**d** Average decoding accuracy within a 400 ms window following whisker touch (shaded area as SEM), plotted as a function of the number of units. The decoding accuracy curves are presented for BCN units alone, touch-modulated units including BCNs, and touch-modulated units excluding BCNs. The red dotted line marks a decoding accuracy threshold of 80%. Data are shown for the four recorded brain regions: BC (**a**), VPM (**b**), POm (**c**), and ZIv (**d**). Data pooled from *n* = 6 mice. Source data are provided as a Source data file.

Next, we addressed whether and to which extend aperture widths can be decoded from BCN activity by training a classifier to predict which aperture was sampled based on unit spike trains from the different recording sites (see Methods). The decoding accuracy of the classifier increased progressively with the number of units used as input. Notably, the performance of the classifier was consistently higher when trained on BCNs compared to other touch-modulated units (Fig. 5a-d). To achieve a decoding accuracy of 80% or higher, the number of units required was significantly lower for the BCN group (7 units for BC, 25 for VPM, and 9 for POm) compared to the touch-modulated group including BCNs (18 units for BC, 76 units for VPM, and 31 for POm) and excluding BCNs (46 units for BC, more than 80 for VPM, and 77 for POm) (two-tailed paired *t*-test with an *α* = 0.05).

To account for potential covariance among simultaneously recorded neurons within the same individual animal, we additionally performed animal-wise decoding analyses in expert sessions before averaging decoding performance across individuals (Supplementary Fig. 9a, b). Consistent with the area-wise analyses, BCNs consistently achieved higher decoding accuracies than both touch-modulated populations, including and excluding BCNs. Fitting decoding curves with a negative exponential saturation model revealed that BCNs exhibited significantly faster saturation dynamics than touch-modulated populations both including BCNs (*p* = 0.0022, paired two-sided *t*-test) and excluding BCNs (*p* = 0.0010, paired two-sided *t*-test), showing that high decoding performance was reached with substantially fewer units. Furthermore, the asymptotic decoding accuracy was significantly higher for BCNs compared to touch-modulated populations excluding BCNs (*p* = 0.0191, paired two-sided *t*-test), whereas no significant difference was observed when including BCNs (*p* = 0.6033, paired two-sided *t*-test). Together, these analyses demonstrate the superior predictive power of BCNs in decoding sensory information and trial outcomes.

### Thalamic burst inhibition impairs learning and task execution

**Fig. 6:**
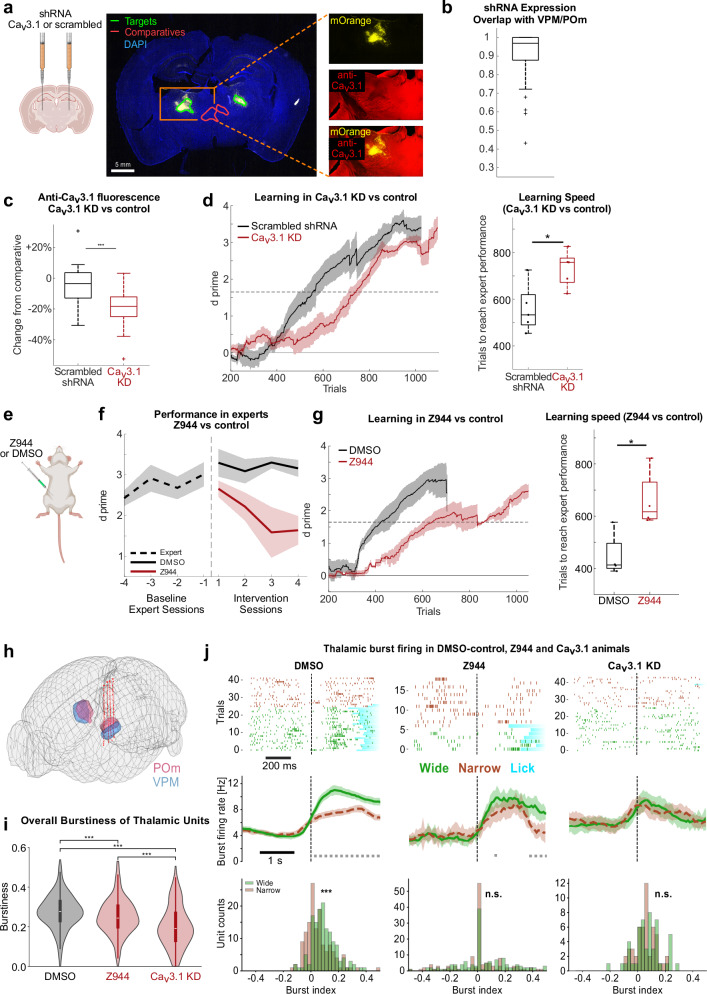
Thalamic burst inhibition impairs learning and task execution. **a** Schematic of shRNA viral injection to knock down (KD) Ca_V_3.1 in the VPM and POm (left panel), including a scrambled shRNA construct used as a control to account for non-specific effects of viral expression. Representative histological image (middle panel) showing shRNA expression (yellow, shRNA-mOrange) in the thalamus with DAPI counterstain (blue). Higher magnification insets (right panels) show mOrange fluorescence (yellow) and Ca_V_3.1 expression (via anti-Ca_V_3.1 immunofluorescence) in KD conditions. Each condition included *n* = 5 animals; histological quantification was performed on *n* = 18 brain slices from 2 scrambled shRNA control animals and *n* = 34 brain slices from 4 Ca_V_3.1 KD animals. **b** Proportion of shRNA expression within anatomical boundaries of VPM and POm (*n* = 34 brain slices from 4 animals). **c** Normalized thalamic anti-Ca_V_3.1 fluorescence intensity of Ca_V_3.1 KD animals (*n* = 34 brain slices from 4 animals) compared to scrambled shRNA controls (*n* = 18 brain slices from 2 animals) (*p* = 1.1514 × 10⁻⁴). **d** Trial-by-trial learning curves in Ca_V_3.1 KD mice compared to scrambled shRNA controls (left panel). Data is shown as cohort mean with SEM as shaded areas. Learning speed quantified as the number of trials to reach expert performance in Ca_V_3.1 KD animals and scrambled shRNA controls (right panel, *n* = 5 mice in each group, *p* = 0.0317). **e** Schematic of systemic Z944 or DMSO (control) i.p. injection in mice. **f** Session-wise performance (d′) in expert animals (dashed grey line) following systemic administration of Z944 (red) compared to DMSO control sessions (grey). Lines represent the cohort means with SEM as shaded areas (4 sessions from *n* = 6 animals). **g** Same as in d for Z944-treated animals and DMSO controls (*n* = 4 mice in each group, *p* = 0.0286). **h** Example reconstruction of a 4-shank Neuropixel recording in POm and VPM. **i** Overall burstiness (see methods) during 15 min recordings in control-DMSO, Z944 and Ca_V_3.1 KD conditions. VPM and POm units were pooled, DMSO and Z944 *n* = 422 (same units), shRNA, *n* = 305 units. Statistical comparison was performed using a Kruskal–Wallis test (H(2) = 111.22, *p* < 0.001). **j** Top: raster plots of example POm units during DMSO and Z944 (left, middle, same unit) and Ca_V_3.1 KD (right). Middle: Burst firing rate for wide (rewarded) and narrow (unrewarded) aperture relative to whisker touch (dashed line). Significant differences (*α* = 0.05) are indicated with dashed lines. Only touch-modulated POm and VPM units were selected (same units in DMSO and Z944 conditions, *n* = 140, Z944, *n* = 140, shRNA, *n* = 51). Bottom: Distribution of mean burst indices for the same units as above. Statistical comparisons performed using two-tailed Wilcoxon signed-rank tests: DMSO, *p* = 1.1 × 10⁻⁵; Z944, *p* = 0.0506; shRNA, *p* = 0.223. n.s.: *p* > 0.05, *: *p* ≤ 0.05, **: *p* ≤ 0.01 and ***: *p* ≤ 0.001; **c**, **d**, **g**: two-tailed Mann–Whitney *U*-Test, **f:** two-tailed, paired t-test, **i:** Kruskal-Wallis with post-tests. **j**: two-tailed Welch’s *t*-test with Benjamini-Hochberg correction for multiple comparisons (middle panels); two-tailed Wilcoxon signed-rank test (lower panels). For detailed description of statistics see Supplementary Data 1. Source data are provided as a Source data file.

To directly test the causal contribution of thalamic bursts to learning and task execution, we selectively suppressed T-type calcium channel–dependent bursting in VPM and POm, given their central role in sensory processing. Viral particles carrying an shRNA construct against Cacna1g were targeted to VPM and POm (Fig. 6a, b), leading to a knock-down (KD) of Ca_V_3.1, the T-type calcium channel responsible for thalamic burst firing^21^. The same viral construct has previously been validated in vitro, where Ca_V_3.1 knock-down in POm neurons abolished burst firing while preserving tonic spiking, demonstrating its specificity for silencing T-type channel–mediated bursts^22^. Adding to that, we further validated the knock-down efficiency by quantifying the reduction of Ca_V_3.1 protein expression, as indicated by a significant decrease in normalized anti-Ca_V_3.1 immuno-fluorescence intensity within the injection sites relative to neighboring non-injected tissue (Fig. 6c). Behavioral testing on the whisker-dependent discrimination task revealed impaired learning in Ca_V_3.1 KD mice, which required significantly more trials to reach threshold compared to scrambled shRNA controls (Fig. 6d; scrambled shRNA: median [IQR] = 533 [490–619.25] trials; Ca_V_3.1 KD: median [IQR] = 723 [649–792.5] trials; *p* = 0.0317; two-tailed Mann–Whitney *U*-Test). To further complement these findings, we employed a pharmacological approach by systemically administering the selective T-type calcium channel antagonist Z944^23^ via i.p. injections, during or after task learning (Fig. 6e). In expert animals, acute Z944 administration significantly reduced discrimination performance (Fig. 6f, DMSO sessions: d’ = 3.2101 ± 0.5903 vs Z944 sessions: d’ = 2.0245 ± 1.0259; *p* = 4.5871 × 10^−6^; two-tailed, paired *t*-test). In a separate experiment, testing the relationship between pharmacological burst suppression and learning, we found that Z944-treated animals exhibited impaired learning curves similar to those seen in Ca_V_3.1 KD mice (Fig. 6g, trials to reach expert performance for DMSO controls: median [IQR] = 413.5 [400.5–496.5] trials; Z944-treated mice: median [IQR] = 617; [590–730.5] trials; *p* = 0.0286; two-tailed Mann–Whitney-*U*-Test). Chronic electrophysiological recordings using Neuropixels 4-shank probes confirmed that both the Ca_V_3.1 knockdown and Z944 effectively suppressed thalamic burst firing (Fig. 6h–j). Across thalamic units, overall burstiness was significantly reduced in both Z944-treated and Ca_V_3.1 KD animals compared to DMSO controls (Fig. 6i), indicating a robust suppression of low-threshold calcium spike–dependent bursting. During task performance, burst firing in control animals was strongly modulated by sensory context, with increased burst probability during whisker contacts in the rewarded (wide) aperture condition (Fig. 6j, middle). This context-dependent modulation was abolished in both Z944 and Ca_V_3.1 KD groups, further confirming that thalamic bursts encode behaviorally relevant sensory information. Correspondingly, the distribution of burst indices was shifted towards indifferent values in both manipulations (Fig. 6j, bottom), confirming a loss of burst-coding. Together, these findings demonstrate that suppression of bursting impairs the encoding of task-relevant sensory events and consequently degrades learning and performance.

## Discussion

We identify neuronal burst activity at the single-cell level as a core mechanism of learning, emerging in burst-coding neurons (BCNs) distributed across cortical, thalamic and extrathalamic regions. BCNs encode and track the stimulus context during different rule stages, with burst coding flexibly aligning with the currently rewarded aperture. During degradation, this rule-related burst bias is reduced, reflecting both an attenuation of aperture-selective burst coding at the single-unit level and a more balanced coexistence of narrow- and wide-encoding BCNs at the population level. This suggests that burst-related reward representations are attenuated during degradation, while the more balanced representation of narrow- and wide-encoding BCNs may also reflect a shift toward a behavioral strategy in which both aperture stimuli are treated as reward-predictive. Consistent with this interpretation, mice licked at high rates to both trial types during degradation. Importantly, the defining feature of BCNs is not a simple increase in firing rate, but a selective change in temporal structure of spike trains that can occur in the presence or absence of corresponding tonic firing changes. Consequently, BCN activity cannot be adequately captured by conventional Poisson-like firing models that assume stochastic spike generation from a rate variable alone, which may have important implications for future learning models and neural network simulations. We further demonstrate that pharmacological or genetic suppression of T-type Ca^2+^ channels, essential for thalamic bursting, disrupted learning, together supporting that thalamocortical bursts couple cellular firing dynamics to associative memory formation. While memory functions have been linked to hippocampal population burst activity, known as sharp wave ripples^24,25^, our work reveals that the thalamocortical system carries its own single neuron burst code that directly drives learning. This possibility is particularly compelling in light of recent findings demonstrating that reinforcement learning induces persistent realignment of apical tuft responses in layer 5 barrel cortex neurons^26^, raising the possibility that thalamocortical burst signals may interact with dendritic integration and plasticity mechanisms in the cortex.

These insights propose the emergence of burst coding during learning as a central mechanism to strengthen synaptic connections between neurons that form the memory engram for task execution. Extensive ex-vivo work has demonstrated that high-frequency neuronal firing modulates long-term synaptic coupling via calcium influx through NMDA receptor channels, a critical process for altering synaptic strength^4,27,28^. Indeed, burst firing has been found to gate cortical plasticity in vivo^29^. Our findings extend this framework by revealing that sensory-evoked burst firing in BCNs may contribute to the formation of associative memories during learning. The sharp increase in BCN proportion during early learning, followed by loss of burst coding during task degradation, indicates that BCNs are most critical during acquisition phases. This relationship between burst firing, learning, and task performance broadens established perspectives on thalamocortical burst function. Previous models, such as the searchlight hypothesis^30^, wake-up call hypothesis^31^, and more recent theories of bursts in perception^16^ and consciousness^32^, focused on bursts as mediators of sensory processing or brain state. Our results extend this perspective by suggesting that burst firing may constitute a neural coding regime involved in learning.

The inversion of burst-coding after rule reversal demonstrates that BCNs track the stimulus context rather than the stimulus’ physical features. Furthermore, burst responses were biased toward the rewarded stimulus and were modulated linearly according to valence, which was particularly evident when a neutral stimulus was introduced in our experiments. These observations corroborate the learning model proposed by Payeur et al.^18^ for synaptic plasticity in hierarchical networks, in which bursts were proposed to convey credit-related information when operating in a non-saturated, linear regime. In addition, the observation of distributed burst coding across all four nodes of the somatosensory pathway, together with recent evidence that reduced hippocampal bursting correlates with cognitive deficits in Alzheimer’s disease^33^, suggests that burst-based coding may represent a general neural principle for learning and memory across modalities.

Importantly, our causal interventions demonstrate that thalamocortical bursts are not mere correlates but essential drivers of learning. Knock-down of Ca_V_3.1 channels in VPM and POm significantly impaired bursting and the acquisition of task performance, demonstrating the necessity of T-type channel–mediated burst firing for establishing stimulus–outcome associations. Likewise, systemic administration of the selective T-type Ca^2+^ channel blocker Z944 reduced burst incidence, slowed learning during training, and disrupted performance in expert animals.

Together, these findings support a model in which thalamocortical bursts encode learned stimulus–outcome contingencies, rather than simply relaying sensory information or driving motor output. BCN responses correlate with task performance, reverse with rule switches, scale with stimulus valence, collapse during degradation, and remain largely independent of measured whisker kinematics and licking variables. This argues against the interpretation that bursts merely reflect stronger tactile input or induce lick execution. Instead, bursts may act as a value-weighted sensory gain signal: they mark stimuli that have acquired behavioral significance and thereby bias downstream representation, attention, and plasticity. This interpretation is consistent with work showing that thalamic bursting can sharpen thalamocortical transmission by increasing timing precision and cortical synchrony^14^, and with computational models in which high-frequency bursts provide credit-related information for synaptic plasticity in hierarchical circuits^18^. Notably, burst bias remained elevated even after stable task acquisition, suggesting that these signals may reflect learned contingencies or state-value representations involved in maintaining and updating sensory–outcome associations, rather than a simple perceptual prediction-error signal, which would be expected to diminish once behavior becomes stable.

## Methods

### Statistics and reproducibility

This study was designed as an exploratory study with sample sizes of at least six animals, and no animal was excluded from the analyses. No statistical method was used to predetermine sample size. The experimenters were blinded during the interventional experiments (Fig. 6) and unblinded after the completion of the analyses. Fast-spiking units were excluded from further analyses of neuronal dynamics in BC, POm and VPM.

### Animals

All experimental procedures received approval from the local governing authority (Regierungspräsidium Karlsruhe, Germany, approval numbers: 35-9185.81/G-216/19 and 35-9185.81/G-145/24) and were conducted in accordance with their ethical guidelines. The study involved adult male C57BL/6NRj mice (Janvier Labs, Le Genest-Saint-Isle, France) aged 8–10 weeks at the onset of training. The mice were individually housed in a ventilated Scantainer (Scantainer Classic, SCANBUR A/S, Karlslunde, Denmark) maintained on a 12 h inverted light/dark cycle (lights off at 7:00 a.m. and on at 7:00 p.m.) with controlled temperature (20–24 °C) and humidity (45–65%). During behavioral training, which was conducted during daytime, mice were subjected to a food control regimen, maintaining their body weight at 85-95% of their initial weight, with ad libitum access to water.

### Behavioral setup

The setup is described in detail in Heimburg et al.^19^ and a parts list, technical drawings, and code can be found on Zenodo^34^. In brief, behavioral testing and recording were conducted on an elevated, dark-toned linear platform featuring lick ports at both ends. Mice traversed an adjustable aperture between two motorized wings to access the lick ports, with aperture widths varying trial-to-trial. Each lick port was equipped with a cannula connected to a syringe filled with condensed milk, and a speaker for white noise delivery. A piezo sensor on the cannula registered lick events that triggered either a reward (10 µL of condensed milk) or a punishment (120 dB noise, lasting 1–3 s). The setup was controlled via Syntalos^19^ (code available at https://github.com/bothlab/syntalos), which synchronized the timing of events (aperture state, reward/punishment, lick detection, and IR beam crossings), cameras, and electrophysiology.

### Behavioral paradigm

The paradigm is inspired by Krupa et al.^35^ and was recently adopted by us for mice as described in detail in Heimburg et al.^19^. In brief, mice were trained on a go/no-go whisker-dependent task on a linear track, learning to discriminate between apertures of different widths to obtain rewards and avoid punishments. Training sessions lasted 15 min, conducted twice daily with at least 2 h break between sessions. After sampling the aperture with their whiskers, mice decided to either lick or turn and run to the opposite end of the track. Aperture states were reset randomly at the beginning of each trial.

#### Initial rule stage

Go (45 mm “wide”) and no-go (25 mm “narrow”) apertures were presented with an equal probability of 50 % in a random sequence. Licking upon the go (wide) aperture triggered a reward, and licking at the no-go aperture triggered a punishment.

#### Neutral aperture stage

Reward and punishment contingencies remained as in the initial rule stage, but an additional “neutral” aperture (35 mm) was added. Licking upon the neutral aperture resulted in no outcome and no scoring. The neutral aperture stage lasted for 8 sessions.

#### Reversed rule stage

Reward and punishment contingencies were inverted (i.e., the previously rewarded aperture was punished, and vice versa). The neutral aperture was not presented.

#### Degradation stage

Aperture states and outcomes were randomized in each trial (50% chance for “wide” or “narrow” and reward or punishment, respectively) in order to degrade the apertures as an outcome predictor. The degradation stage lasted for 8 sessions.

### Data acquisition

Data acquisition during behavior was done with Syntalos^19^. An Intan module recorded all analog signals from the lick ports. The overview camera was connected to a recorder module that operated continuously throughout the session. The high-speed cameras and an event list were controlled by Syntalos via a custom-made Python script. The cameras were programmed to start recording if the animal approached the corresponding lick port, determined by the crossing of the corresponding light beam. The event list kept track of the triggered light beams and reported the state of the apertures. The list also noted if each trial was a success or failure.

### EIB manufacturing

Electronic Interface Boards (EIBs) were based on the design developed in Oettl et al.^36^. Details, such as technical drawings, can be found in Heimburg et al.^19^. In brief, approximately 30 cm of tungsten wire (12.5 μm, Item # 100211, Tungsten 99.95% CS Wire, California Fine Wire Company) was twisted into a tetrode. Sixteen tetrodes, providing a total of 64 recording channels, were mounted into a custom-designed EIB (produced by Multi-Circuit-Boards). The layout allowed for proper alignment of the tetrodes in the x-y plane. A custom-built scaffold was used to position the tips of the tetrodes before fixation on the EIB to ensure proper depth positioning of each tetrode during implantation (see Table 1). Tetrodes were then fixed to the EIB using UV-curable adhesive (3 M™ Filtek™ Supreme XTE, 3 M Deutschland GmbH, Neuss, Germany), and the opposite ends of the tetrode tungsten wires were then soldered onto the copper contacts of the EIB. Prior to implantation, the electrodes were suspended in a gold chloride solution (200 mg/dL gold chloride in deionized water, HT1004-100ML, Sigma-Aldrich, Inc., MO, USA) and gold plated to reduce their individual impedances to less than 100 kOhm. To test whether the channels conduct the signal well, a function generator (A365 Stimulus Isolator, World Precision Instruments, Inc., FL, USA) was connected to a basin with sodium chloride, in which the tetrodes were submerged. Only EIBs with ≥62 conducting channels were used for implantation.

**Table 1:** Tetrode targeting coordinates

Tetrode	A/P [mm]	M/L [mm]	D/V with respect to dura [mm]
BC 1	−1.0	3.25	−0.4
BC 2	−1.3	3.3	−0.3
BC 3	−1.6	2.7	−0.85
BC 4	−1.3	2.6	−0.85
BC 5	−1.0	2.7	−0.85
VPM 1	−1.95	1.95	−2.95
VPM 2	−1.95	1.6	−2.95
VPM 3	−1.75	1.75	−3.0
VPM 4	−1.45	1.6	−2.95
POm 1	−2.15	1.3	−2.75
POm 2	−2.3	1.5	−2.75
POm 3	−2.3	1.15	−2.8
POm 4	−2.0	1.05	−2.85
ZIv 1	−2.35	2.0	−3.7
ZIv 2	−2.6	1.75	−3.7
ZIv 3	−2.15	1.75	−3.75

*A/P* antero-posterior, *M/L* medio-lateral, *D/V* dorso-ventral.

### Surgical procedures for in vivo experiments

Thirty minutes prior to inducing general anesthesia, buprenorphine hydrochloride (Bupresol vet. Multidose 0,3 mg/ml, CP-Pharma Handelsgesellschaft mbH, Burgdorf, Germany) or carprofen (5 mg/kg BW, CP-Pharma) was administered subcutaneously at a dosage of 0.1 mg/kg of body weight. General anesthesia was induced using isoflurane (1–2% in oxygen, Isofluran Baxter, Baxter Deutschland GmbH, Germany), with careful monitoring and control of the eyelid reflex and pedal withdrawal reflex. Body temperature was maintained between 36 and 38 °C using heating pads. Eyes were protected from drying using corneal application of eye ointment (Bepanthen®, Bayer, Germany). The scalp was locally infiltrated with Lidocaine (Xylocaine® 1%, Aspen Pharma Trading Limited, Ireland) to anesthetize the surgical area. The animal was positioned in a non-traumatic stereotactic alignment apparatus (David Kopf Instruments, Tujunga, CA, USA). Small holes were drilled in the skull over the target recording sites using a dental drill (for exact coordinates see Table 1 below; 78001 Microdrill, RWD Life Science, TX, USA). The EIB was then gradually lowered into the brain until the desired depth was reached. The EIB was attached to the skull using a cyanoacrylate-based adhesive (Super-bond, Sun Medical, Japan) and dental cement (Paladur®, Kulzer, Germany). Following the surgeries, the animals were placed on a heated surface within a pre-warmed enclosure before being returned to the ventilated cabinets (Scantainer Classic, SCANBUR A/S, Karlslunde, Denmark). Behavioral experiments started following an adequate period of recovery subsequent to EIB implantation. The tetrode array containing 16 tetrodes (total of 64 recording channels), was implanted in the barrel cortex (BC; 5 tetrodes), the ventral posteromedial nucleus (VPM; 4 tetrodes), the posterior medial complex (POm; 4 tetrodes), and the ventral zona incerta (ZIv; 3 tetrodes). Coordinates are given with respect to bregma, according to^37^:

### Histology and identification of tetrode positions

After administering Ketamine-Xylazine through intraperitoneal injection (ketamine dosage: 120 mg/kg bw, CP-Pharma Handelsgesellschaft mbH, Burgdorf, Germany; xylazine dosage: 20 mg/kg bw, Xylavet^®^ 20 mg/ml, CP-Pharma Handelsgesellschaft mbH, Burgdorf, Germany), a transcardial perfusion with paraformaldehyde (PFA, 4% in phosphate-buffered saline [PBS]) was performed. The mouse’s head, along with the EIB implant, was post-fixed in PFA (4% in PBS) for a period of five days at 4 °C. Subsequently, the EIB was removed, the brain was cut into 50 µm thick sections with a vibrating microtome (Thermo Scientific Microm HM 650 V), and the tetrode traces were identified using a bright-field microscope. Through the digital delineation of tetrode marks in each section, we were able to reconstruct the complete trajectory of each tetrode in three dimensions using the Amira software (v6.5, Thermo Fisher Scientific, Waltham, MA). Subsequently, all tetrodes located outside of the designated target regions were excluded from further analysis.

Reconstruction files can be found on GitHub (https://github.com/GrohLab/Thalamocortical-bursts-encode-reward-contingencies-and-drive-associative-learning/tree/main/Data/Reconstructions) and Dryad (10.5061/dryad.hdr7sqvt1).

### shRNA-mediated knock-down of Ca_V_3.1

To suppress thalamic burst firing, we employed an shRNA-based knock-down strategy targeting the Cacna1g gene encoding the Ca_V_3.1 T-type calcium channel, following the approach described in Seol and Kuner^22^. Briefly, short hairpin RNA (shRNA) sequences against Cacna1g or a scrambled sequence as control were cloned into an adeno-associated viral (AAV) vector under the U6 promoter. Reporter expression (mOrange) was driven by the CAG promoter, enabling identification of injection sites. For stereotaxic injections (same surgical procedures as above), mice were anesthetized with isoflurane, and small craniotomies were made above the target sites. A pulled glass micropipette was backfilled with viral solution and connected to a precision syringe. AAV-shRNA (100 nL per site) was then pressure-injected bilaterally into the VPM and POm using stereotaxic coordinates relative to bregma (POm: LM = -1.15/ + 1.15, AP = -1.7, DV = -3.0; VPM: LM = -1.6/ + 1.6, AP = -1.6, DV = -3.3/-3.2). The injection pipette was left in place for 10 min to minimize reflux before being retracted. Animals were allowed to recover for at least 3 weeks before behavioral training and electrophysiological recordings to ensure stable expression of the knock-down construct. To validate that the knock-down efficiently suppressed thalamic bursting, neuropixel recordings were obtained from Ca_V_3.1 shRNA mice and reductions in burst measures were quantified (see below).

### Histological validation of knock-down and atlas registration

Brains were extracted and post-fixed as described above, before being coronally sectioned at 60 µm thickness. Sections containing the thalamus were processed for immunohistochemistry using an antibody against the Ca_V_3.1 channel (Anti-CACNA1G (Ca_V_3.1) Antibody, #ACC-021, Alomone Labs, Jerusalem 9104201, Israel), diluted 1: 200 in phosphate buffer, and counterstained with DAPI to visualize cytoarchitecture. Injection sites were identified by reporter gene expression (mOrange), and corresponding signals were imaged at high resolution. Injection volumes were reconstructed using a custom MATLAB script (publicly available, see Code availability). Briefly, regions of reporter signal were manually delineated to generate binary target masks of injection sites. For each slice, the target masks were used to extract fluorescence values from the Ca_V_3.1 immunostaining channel. 32-bit integer intensities were capped between 0 and 200, normalized to a range of 0–1, and averaged across pixels within the target mask to obtain normalized Ca_V_3.1 expression values. To assess regional specificity, the pipeline randomly projected masks of identical size as the target masks into non-injection thalamic regions. For each selection mask, normalized Ca_V_3.1 intensity values were compared to values from these randomly placed comparative masks within the thalamus. To evaluate targeting accuracy in the shRNA group, the Allen Mouse Brain Atlas was overlaid onto corresponding histological sections, and the proportion of injection volume overlapping with VPM and POm was calculated.

### Pharmacological suppression of thalamic bursting with Z944

Z944 (Ulixacaltamide) is an inhibitor of low-voltage–activated (T-type) Ca²⁺ channels (mainly Ca_V_3.1). According to Harding and Zamponi^23^, Z944 is one of the most selective T-type channel inhibitors, avoiding greater off-target effects as seen with ethosuximide^38^. Z944 (Z944 hydrochloride, #SML2635, Sigma-Aldrich Chemie GmbH, Taufkirchen, Germany) was prepared in a dimethyl sulfoxide (DMSO)-based vehicle (1% DMSO in 0.5% methylcellulose), with a concentration of 10 mg/kg BW (similar to Gambeta et al.^39^). Administration of vehicle alone served as control. Solutions were administered intraperitoneally (i.p.) at a fixed volume of 4 µL/g BW 15 min prior to behavioral testing. To validate that Z944 effectively suppressed thalamic bursting, electrophysiological recordings were obtained from treated mice, and reductions in burst measures were quantified (see below).

Within-subject intervention in expert animals (performance disruption): To assess whether ongoing task execution depends on T-type–mediated bursting, we employed a crossover design in mice that had already reached expert performance. Each animal underwent repeated behavioral sessions under vehicle (DMSO) or Z944 in alternating order.

Between-subject intervention from training onset (learning acquisition): To test whether T-type–dependent bursting is required for learning, a separate cohort received either Z944 or vehicle beginning with the first training session and continuing throughout the acquisition phase. The primary endpoint was trials-to-expert threshold. A fixed injection schedule and session timing were maintained across days as described above.

### Electrophysiological validation of pharmacological and genetic burst suppression

Mice were implanted with 4-shank Neuropixels 2.0 probes targeting the left POm and VPM. To protect the implant, a modified 3D-printed head cap system was used (based on^40^). Metal microdrives^40^ were used to attach the probes to facilitate later probe retrieval. Coordinates relative to bregma were: 1.8 mm posterior, 1.3 mm lateral, and −4.0 mm ventral (relative to brain surface). Before implantation, probes were dipped in 1 μL of CM-DiI solution (Thermo Fisher, 50 μg dissolved in 50 μL of pure ethanol^41^). The head cap base, metal microdrive, and the stainless-steel reference screw (19010-10, Fine Science Tools) were attached to the skull using Super-Bond (Sun Medical) and light-curable epoxy (Gradia Direct Flo, GC Corporation). The exposed craniotomy was covered with a thin layer of Vaseline. The ground screw was wired to the probe, and the Neuropixels headstage was secured inside the head cap. All other steps of the surgery, such as anaesthesia and analgesia, were the same as for the tetrode implants. After histological processing of brain slices, the position of each probe shank was registered to a reference brain using HERBS^42^ and SHARP-Track^43^.

### Data analysis

The behavioral data were analyzed using custom code developed in MATLAB R2023a (The MathWorks, Inc.), which is available for reference on GitHub

(https://github.com/GrohLab/Thalamocortical-bursts-encode-reward-contingencies-and-drive-associative-learning) and Zenodo (10.5281/zenodo.21249852).

*P*-values below 0.05 were considered significant. Mean values were reported with standard deviations and median values with interquartile range (IQR).

### Statistical analysis of the learning process

Task performance in each session was measured using the discriminability index d-prime (d’), also known as Fisher discrimination index, as described previously^44,45^ defined as the difference between the *z*-scores of the hit rate and the false alarm rate: $d{\prime}=z({hit}\,{rate})-z({false}\,{alarm}\,{rate})$. Due to the constraints of the z-score transformation (which cannot handle proportions of 0 or 1), hit rates and false alarm rates of 0 are typically adjusted to ($\frac{1}{2*n}$) and rates of 1 are adjusted to ($1-\frac{1}{2*n}$), with n being the number of go (for hit rate adjustment), or no-go trials (for false alarm rate adjustment), respectively. A higher d’ indicates greater discriminability, and thus a greater discrimination performance of the mice. A d’ of 0 describes a discrimination performance at chance level. The criterion for achieving expert performance was established at a d-prime of 1.65, corresponding to a one-tailed significance level of *α* = 0.05.

### Extraction of behavioral data from videos

Spatial information of the mice was extracted from video recordings using DeepLabCut^20^. Following preprocessing with median background image subtraction, the DeepLabCut model was trained on a subset of manually annotated video frames. For the high-speed videos, these markers consisted of eight data points for two whiskers on each side from whisker base to tip, and markers for the mouse’s head contours. In overview videos, spatial positioning and velocity were estimated, based on anatomical landmarks on the mouse’s body and head.

### Electrophysiological data

The raw electrophysiological data was spike sorted with KiloSort 2.0. Subsequently, the sorted clusters were curated automatically using the Ecephys spike sorting algorithm provided by the Allen Brain Observatory (code available at https://github.com/alleninstitute/ecephys_spike_sorting). The algorithm includes noise templates to identify and exclude clusters exhibiting characteristics indicative of noise, such as irregular waveform shapes and inter-spike interval (ISI) histograms. The following quality metrics were then applied to further exclude any clusters indicative of multi-unit activity: isolation distance (computed from Mahalanobis distance) greater than 15 and ISI violation below 3%, with an ISI threshold of 1.5 ms. The criteria were selected on the basis of previous studies^46–48^, where they have been demonstrated to effectively distinguish well-isolated single units from noise and multi-unit activity. The individual units were further classified into fast spiking (FS) and regular spiking (RS) units, putatively representing interneurons and excitatory neurons, respectively^49^. The threshold for differentiating RS from FS units was set to a trough-to-peak duration of 350 µs for cortex and zona incerta, and 300 µs for thalamic nuclei (Supplementary Fig. 2). These thresholds align with previous studies^50,51^ and consider the fact that extracellular waveforms in the thalamus are shorter than those in the cortex^50,51^. For further analysis, only RS units were selected for BC, VPM, and POm, while FS/RS units were selected for ZIv because of limited data in the literature in which FS/RS units in the ZI were associated with specific cell types (i.e., inhibitory/excitatory).

### Neuropixels data analysis

Neuropixels recordings were done using a OneBox (Imec). Data were acquired using SpikeGLX (https://github.com/billkarsh/SpikeGLX) at 30 kHz. Data from pharmacological manipulations were concatenated and processed using CatGT (https://github.com/billkarsh/CatGT). Further processing was done using SpikeInterface^52^ and Kilosort4^53^, followed by manual curation in Phy (https://github.com/cortex-lab/phy). We pooled data from POm and VPM and used only RS units for further analysis.

### Neural response metrics

#### Whisker-touch and locomotion responsiveness

Whisker touch responsiveness was defined by a significant increase in firing rates upon aperture touch within a 200 ms response window following aperture touch (to either of the two aperture states) relative to the baseline window (600–400 ms before touch), analyzed using a two-sided Wilcoxon signed rank test. Locomotion-encoding units were identified through stepwise multiple linear regression analysis. This method is used to identify the significant predictors of a dependent variable by iteratively removing the least significant variables until all remaining variables are significant^54^.

Bursts: Bursts are characterized as sequences of two or more spikes with an ISI of ≤ 10 ms and a tail ISI of > 15 ms, in accordance with the criteria established previously^55,56^.

#### Burstiness

To compute the burstiness per unit, burst spikes were first labelled using the above criteria. Then, burstiness is the fraction of burst spikes among all spikes in the overall analysis window (for example, the whole recording after drug injection).

#### Burst index

The burst index (${BI}$) is calculated as the difference between the proportion of burst spikes (${P}_{{bsp}}$) in the response window (0–200 ms after touch) relative to the baseline window (600–400 ms before touch). ${P}_{{bsp}}$ is defined as the number of burst spikes (${N}_{{bsp}}$) over all spikes (${N}_{{sp}}$) in a given window. In cases where no spikes are present within a window (${N}_{{sp}}$ = 0), ${P}_{{bsp}}$ is defined as 0 to allow inclusion of units that are exclusively bursting in one window. Burst indices can range between -1 (maximal burst proportion in baseline with minimal burst proportion in response window) and 1. The formula is given by: ${BI}={P}_{{bsp},{response}}-{P}_{{bsp},{baseline}}=\frac{{N}_{{bsp},{response}}}{{N}_{{sp},{response}}}-\frac{{N}_{{bsp},{baseline}}}{{N}_{{sp},{baseline}}}$

#### Burst bias, definition of BCNs

The burst bias (${BB}$) is derived from the burst indices of two different aperture conditions (${{BI}}_{1}$ and ${{BI}}_{2}$). It is calculated as the difference between the two burst indices, divided by two. Values can range between −1 (maximal burst bias for aperture 2) and 1 (maximal burst bias for aperture 1). The formula is given by: ${BB}=\frac{{{BI}}_{1}-{{BI}}_{2}}{2}$

A significant burst bias, independent of any concurrent changes in tonic firing, classifies the unit as a burst-coding neuron (BCN), as determined by a Wilcoxon rank sum test.

#### Burst fraction

The burst fraction (${f}_{b}$) is defined as the number of burst events (${N}_{{be}}$), disregarding the number of spikes within bursts, divided by the total event rate (${N}_{e}$), which includes both tonic spikes (${N}_{{tsp}}$) and burst events, as characterized in Friedenberger et al.^8^. The formula is given by: ${f}_{b}=\frac{{N}_{{be}}}{{N}_{e}}=\frac{{N}_{{be}}}{{N}_{{be}}+{N}_{{tsp}}}$

#### Tonic index

The tonic index (${TI}$) is calculated as the quotient of the number of tonic spikes (${N}_{{tsp}}$) in the response window (0 to 200 ms after touch) divided by the overall number of tonic spikes in the response and baseline windows (600–400 ms before touch) combined. Values can range between 0 (only tonic spiking in the baseline window) and 1 (only tonic spiking in the response window). The formula is given by: ${TI}=\frac{{N}_{{tsp},{response}}}{{N}_{{tsp},{response}}+{N}_{{tsp},{baseline}}}$

#### Tonic bias, definition of TCNs

The tonic bias (${TB}$) is derived from the tonic indices of two different aperture conditions (${{TI}}_{1}$ and ${{TI}}_{2}$). It is calculated as the difference between the two tonic indices. Values can range between −1 (maximal tonic bias for condition 2) and 1 (maximal tonic bias for condition 1). The formula is given by: ${TI}={{TI}}_{1}-{{TI}}_{2}$

A significant tonic bias in the absence of burst bias classifies the unit as a tonic-coding neuron (TCN), as determined by a Wilcoxon rank sum test.

#### Definition of NCNs

Whisker-touch-modulated units that were neither BCNs nor TCNs were defined as non-coding neurons (NCNs), i.e., neither their tonic firing rates nor their burst rates covaried with the aperture width.

### Surrogate spike-time shuffling for dissociating burst from firing rate coding

To test whether the number of burst-coding neurons (BCNs) and tonic-coding neurons (TCNs) exceeded expectations based on firing-rate statistics alone, we generated surrogate spike trains for each recording session. For every neuron and trial, the number of spikes occurring within the baseline (−600 to −400 ms) and response (0–200 ms) windows relative to the trigger was preserved. Spike times within these windows were then randomly reassigned across neurons by sampling from the pooled distribution of relative spike times across all units in the session, while leaving spikes outside these windows unchanged. This procedure preserved per-neuron spike counts and the overall temporal structure of population-level responses relative to the trigger while removing neuron-specific burst timing structure. For each session, 1000 surrogate datasets were generated, and burst and tonic coding were recomputed using the same analysis pipeline as for the real data.

For each session, Monte-Carlo *p*-values and z-scores were computed by comparing the observed number of BCNs or TCNs to the surrogate distribution. The *p*-value corresponded to the proportion of surrogate datasets in which the number of coding neurons was at least as high as in the real data. To assess significance across sessions, the resulting session-wise Monte–Carlo *p*-values were combined using Fisher’s method, yielding a single combined *p*-value for the dataset. This procedure tests whether the observed deviations from the surrogate expectation across sessions are collectively unlikely under the null hypothesis.

### Neural decoding analysis

The decoding of aperture states from neural spike time data was performed using the Neural Decoding Toolbox^57^. Classifier training was performed with an increasing number of units of either solely BCNs, touch-modulated units including BCNs, or touch-modulated units excluding BCNs. For each configuration, the dataset was divided into training (90% of labels) and test set (10% of labels). When insufficient units were available for partitioning, the test set was selected from the remaining units. Each set contained the spike traces of individual neurons for a given trial and the corresponding aperture labels. A support vector machine (SVM) classifier was trained using the LIBSVM software package. The classifier underwent 10-fold cross-validation, involving separate training and testing across different data partitions. To assess variability in decoding accuracy, each unit selection was bootstrapped 20 times. The mean decoding accuracy was calculated across a time window from trigger onset to 400 ms post-trigger onset.

Additional animal-wise decoding analyses were performed to preserve within-animal covariance structure and assess generalizability across individuals. For these analyses, decoding was computed independently for each animal using only expert sessions (d′≥1.65), and decoding curves were subsequently averaged across animals.

To quantitatively compare decoding dynamics between neuronal populations, decoding accuracy as a function of unit number was fitted for each animal and condition using a negative exponential saturation model,

$${Acc}(n)=A-(A-{A}_{0}){e}^{-{kn}}$$

where ${A}_{0}$ represents baseline decoding accuracy at a hypothetical unit count of zero, A denotes the asymptotic decoding accuracy, and k the saturation rate constant describing how rapidly decoding performance approaches asymptote with increasing unit number. Fits were obtained using nonlinear least-squares regression. Paired two-sided t-tests were then used to compare fitted parameters across decoding conditions across animals.

### Reporting summary

Further information on research design is available in the Nature Portfolio Reporting Summary linked to this article.

## Supplementary information

Supplementary Information

Description of additional supplementary files

Supplementary Movie 1

Supplementary Movie 2

Supplementary Movie 3

Supplementary Data 1

Reporting Summary

Transparent Peer Review file

## Source data

Source Data

## Data Availability

The datasets generated and analyzed in this study have been deposited in the Dryad Digital Repository under the following accession code: 10.5061/dryad.hdr7sqvt1. Source data are provided with this paper.

## Code availability

The code used in this study is available in both a Dryad repository^58^(10.5061/dryad.hdr7sqvt1) and a GitHub repository (10.5281/zenodo.21249852).
