# Supplementary material for: Thalamocortical bursts encode reward contingencies and drive associative learning
Source: Nat Commun. 2026 Aug 11;17:8170. doi: 10.1038/s41467-026-76581-6 (PMC13463074; doi:10.1038/s41467-026-76581-6)
Supplement: Supplementary file 1 [file 41467_2026_76581_MOESM1_ESM.pdf]

Supplementary information for

Thalamocortical bursts encode reward contingencies and drive associative learning

Filippo Heimburg*[,1], Nadin Mari Saluti[1], Lars-Lennart Oettl[1], Josephine Timm[1,2], Katharina Ziegler[1], Maria Helena Bortolozzo-Gleich[1], Thomas Kuner[3] & Alexander Groh*[,1]

[1] Medical Biophysics, Institute for Physiology and Pathophysiology, Heidelberg University, 69120, Germany

[2] Current address: Institute for Experimental Epileptology and Cognition Research, University of Bonn, 53105, Germany

[3] Institute for Anatomy and Cell Biology, Heidelberg University, 69120, Germany

*e-mail: fheimburg@gmx.de (FH); groh@uni-heidelberg.de (AG)

a

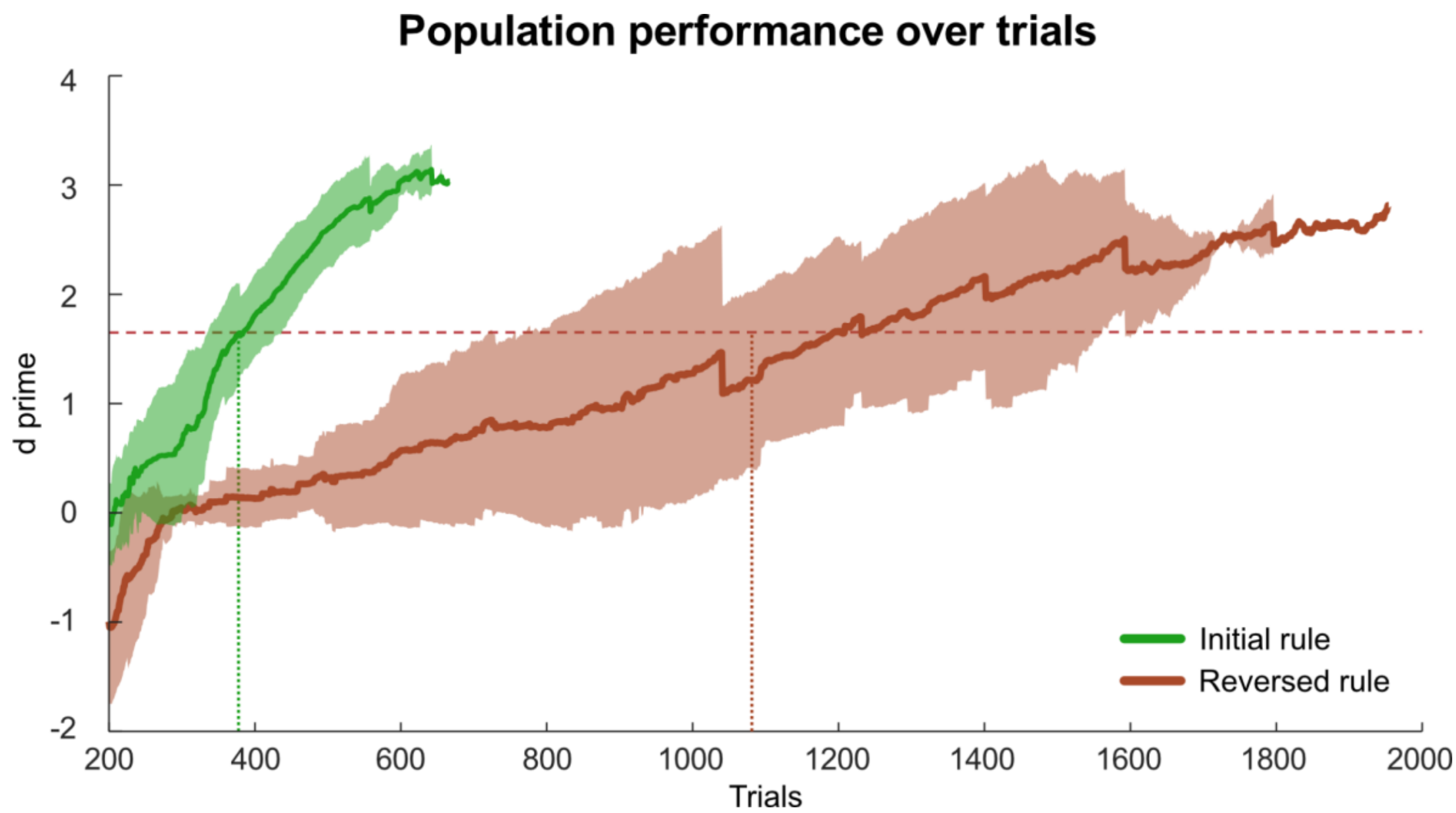

Supplementary Fig. 1 | Population performance over trials. Mean d' values are depicted as solid lines for both the initial rule (green) and reversed rule (orange), calculated using a running window of the preceding 200 trials. Shaded areas represent the standard deviation of the d' values. The horizontal dashed line indicates the expert-level performance threshold (d' = 1.65). Data was pooled from n = 6 mice. The dotted vertical lines mark the trials at which mice on average reached expert-level performance: 388±53 trials during the initial rule stage and 1077±330 trials during the reversed rule stage. Data pooled from n = 6 mice.

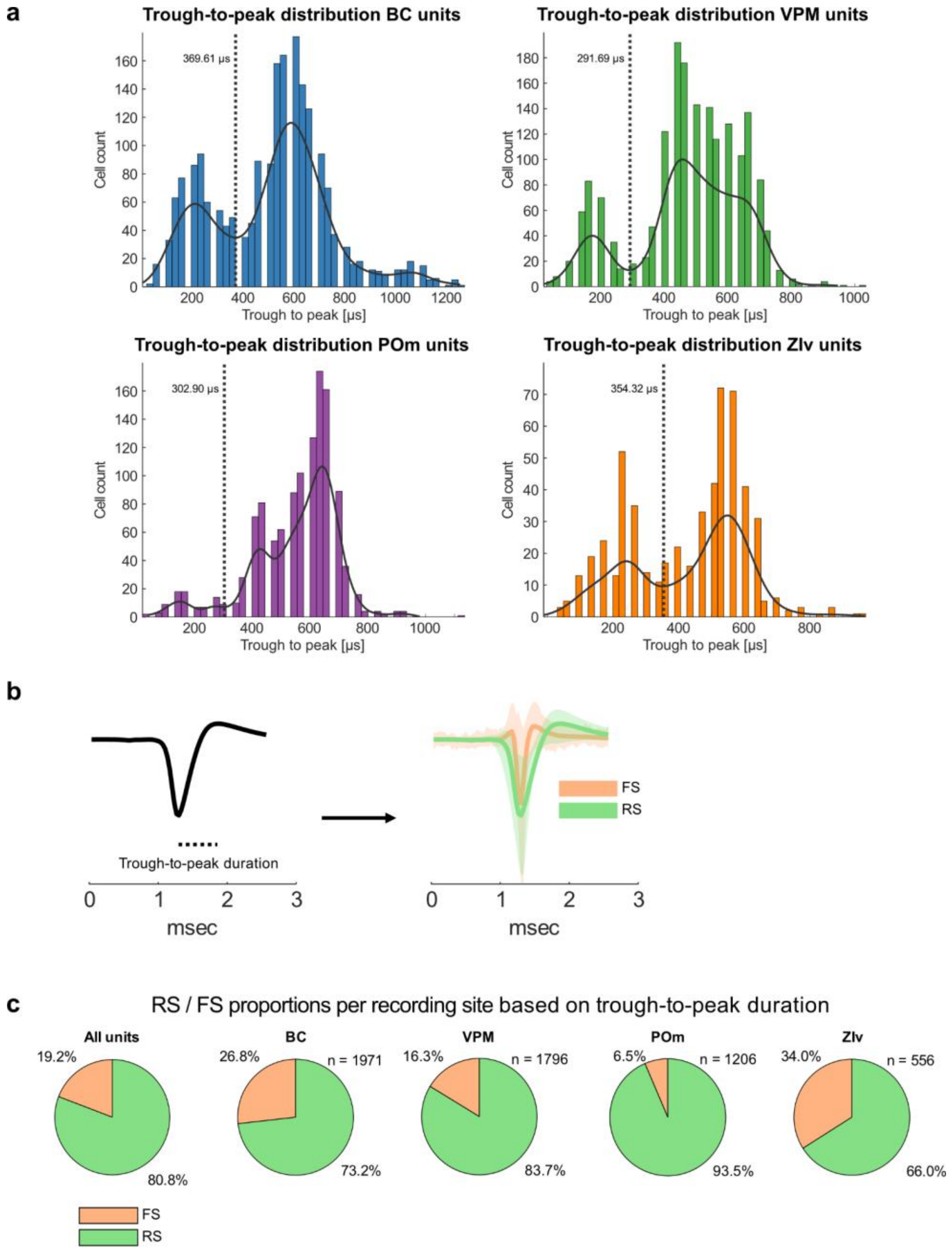

Supplementary Fig. 2 | Unit waveform analysis across brain regions. a, Distribution of trough-to-peak durations per recorded brain region: BC (blue), VPM (green), POm (purple), and ZIv (orange), pooled from n = 6 mice. The dashed vertical lines represent the local minima that best distinguishes between regular-spiking (RS) and fast-spiking (FS) units in each region. **b,** Graphical illustration of the trough-to-peak duration metric, which is defined as the time interval between the trough and the second peak of the extracellular spike waveform. The overlaid waveforms (mean ± SEM) depict the two distinct subpopulations (RS and FS units). **c,** Breakdown of the proportion of RS (green) and FS (orange) units (putative excitatory- and inhibitory neurons, respectively [1]) for each brain region pooled from n = 6 mice.

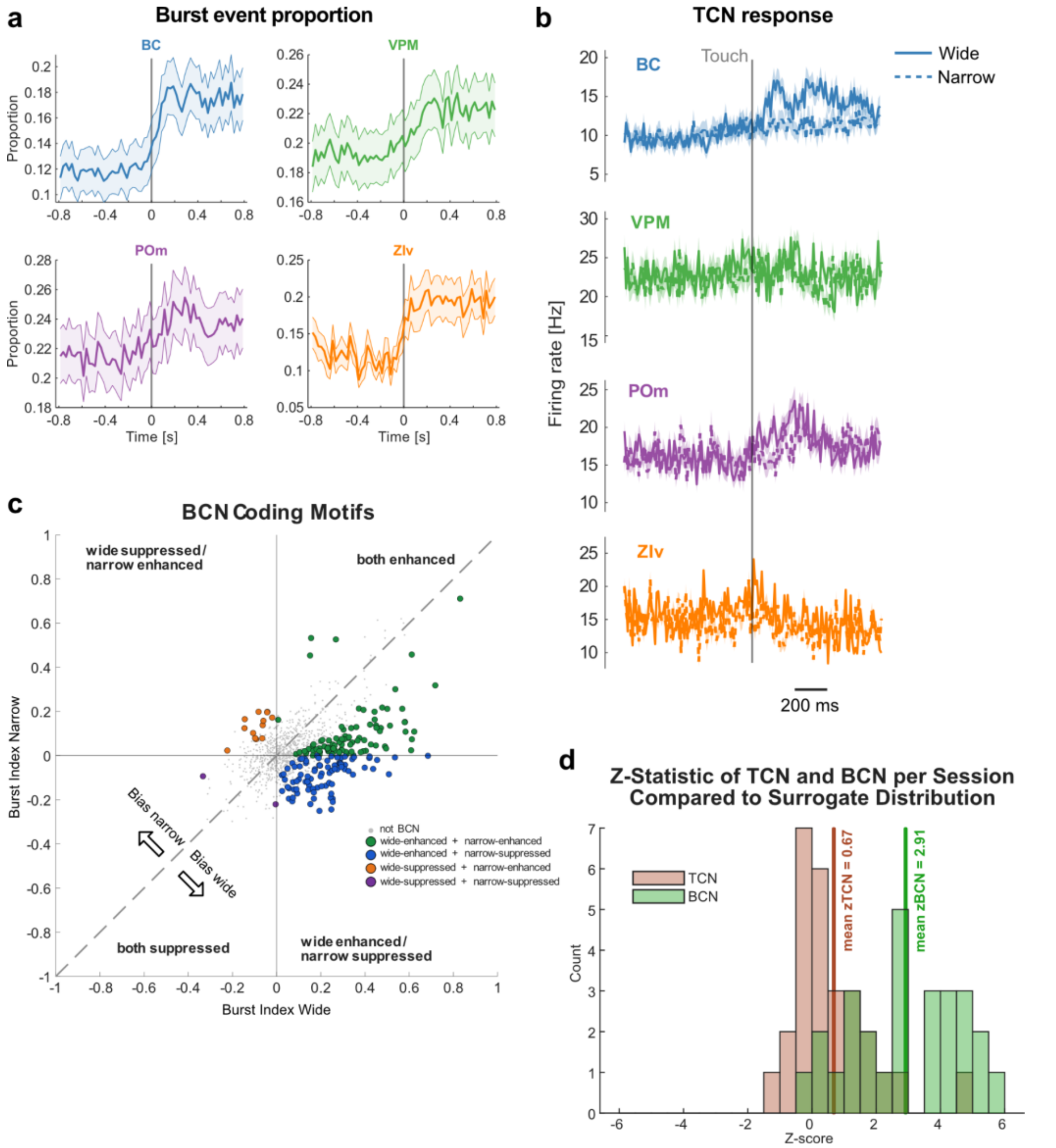

Supplementary Fig. 3 | TCN whisker-touch responses and burst event proportions in expert animals of the initial rule. a, Burst fractions[2] of touch-modulated units in response to whisker touches of the aperture (wide and narrow pooled). The mean burst event proportions develop from baseline values of 11.7 % (BC), 21.2 % (POm), 19.1 % (VPM), and 11.7 % (ZIv), to values after whisker touch of 17.2 % (BC, max. 18.7 %), 24.0 % (POm, max. 25.5 %), 21.8 % (VPM, max. 23.1 %), and 19.2 % (ZIv, max. 21.0 %). Data pooled from n = 6 mice. **b,** Tonic spike rates of TCNs in response to whisker touches of the wide (solid) or narrow (dashed) aperture. Firing rates in response to the two apertures do not differ significantly (two-tailed Welch's t-test). Data pooled from n = 6 mice. **c,** Two-dimensional scatter plot of burst indices for individual neurons across all brain areas during wide- and narrow-aperture sampling (see Methods for burst index definition). Each point represents one recorded unit (n = 1241 units). Grey points indicate non-BCNs, colored points indicate BCNs classified by four distinct coding motifs. The dashed diagonal indicates equal burst modulation for both aperture conditions; units above the diagonal exhibit a burst bias toward the narrow aperture, units below the diagonal are biased toward the wide aperture. **d,** Histogram of session-wise z-statistics quantifying the deviation of the observed BCN and TCN populations from corresponding surrogate distributions. Positive z-scores indicate stronger-than-expected enrichment relative to surrogate populations. Vertical lines indicate the mean z-score for each population.

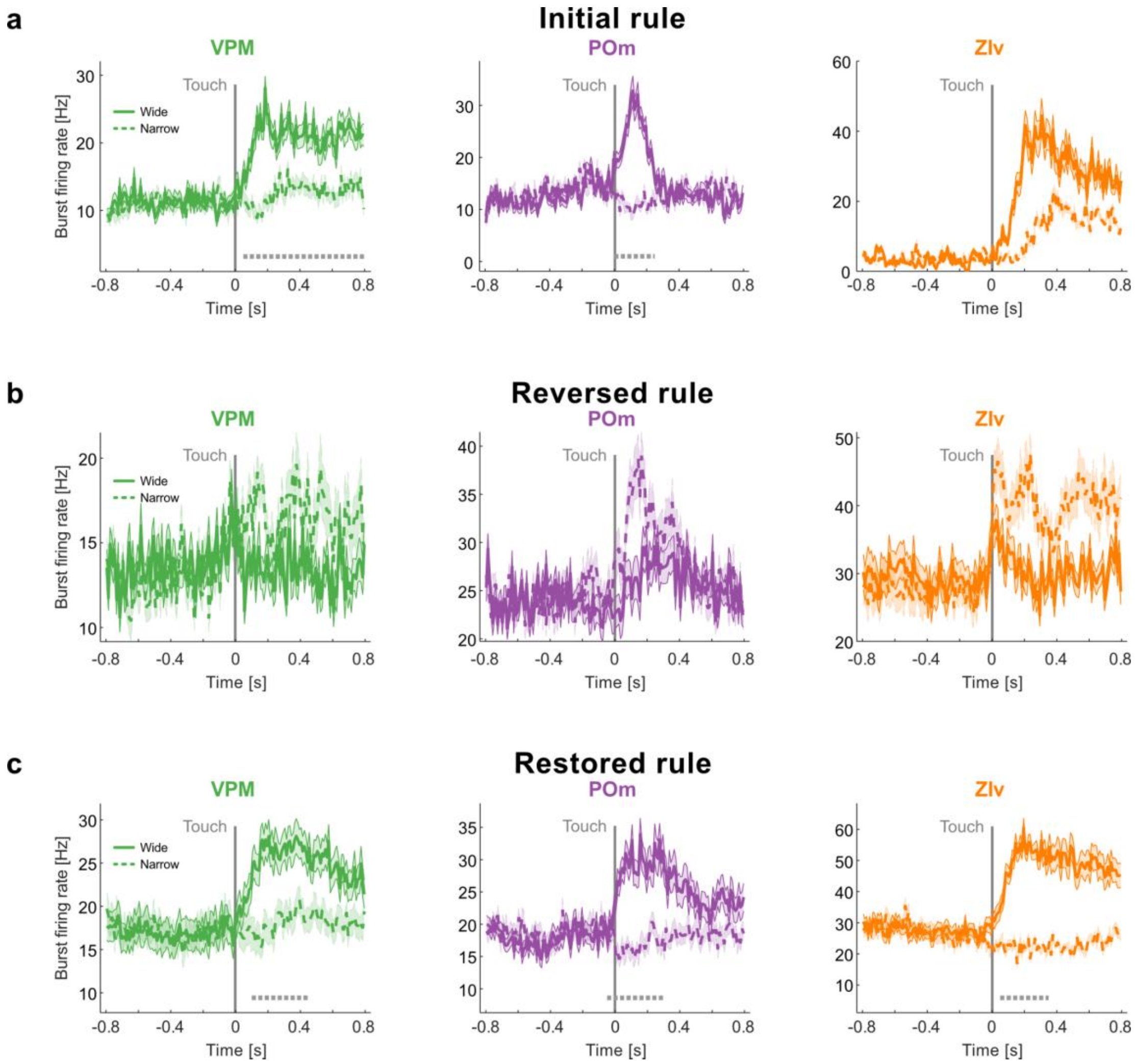

Supplementary Fig. 4 | Burst firing rates during different rule stages across brain regions. Burst firing rates in VPM (left, green), POm (middle, purple), and ZIv (right, orange) in response to wide (solid line) and narrow (dashed line) apertures, represented as mean ± SEM. Statistically significant differences (α = 0.05, two-tailed Welch's t-test) are indicated with dashed lines beneath the plot. Firing rates are shown for the initial rule (**a**), reversed rule (**b**), and the restored rule (**c**). Data pooled from n = 6 mice.

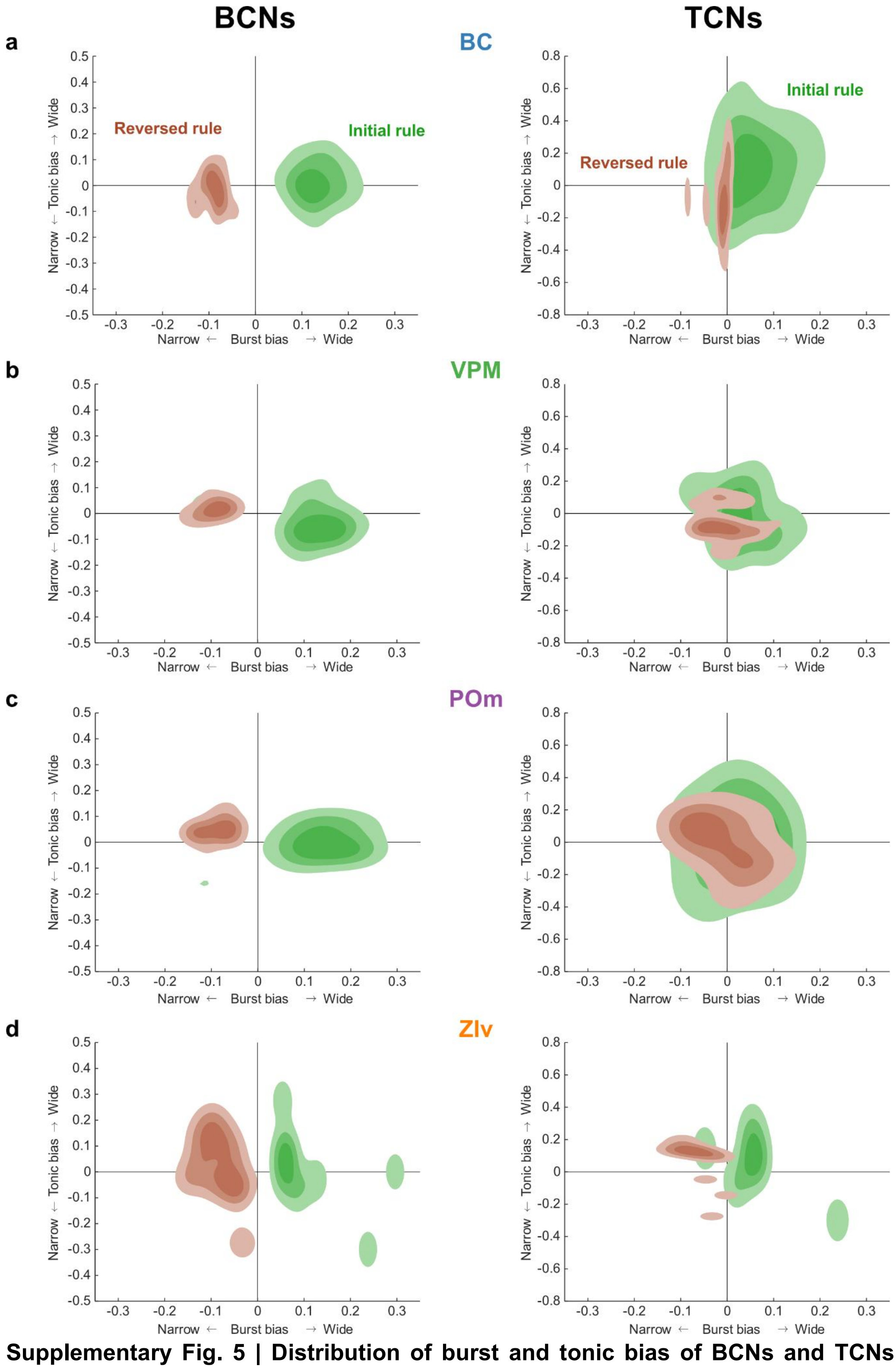

Supplementary Fig. 5 | Distribution of burst and tonic bias of BCNs and TCNs across brain regions. Distributions of burst and tonic bias from BCNs (left) and TCNs (right), for BC (**a**), VPM (**b**), POm (**c**), and ZIv (**d**). The initial rule is shown in green and the reversed rule in maroon. Contour lines represent the first quartile (Q1), median (Q2), and third quartile (Q3) of the units in the scatter plot. Data pooled from n = 6 mice.

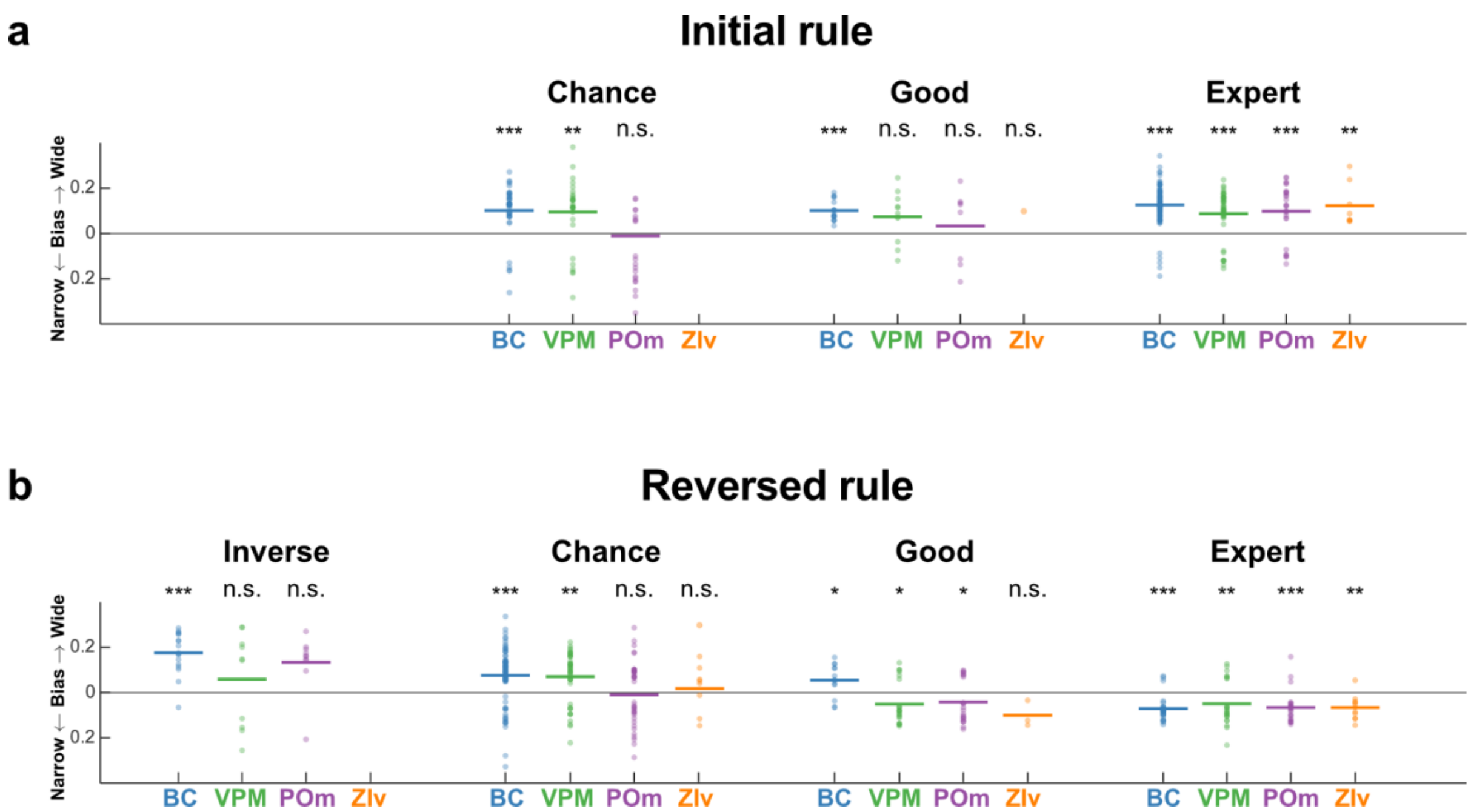

Supplementary Fig. 6 | Burst bias progression for individual BCNs during learning across brain regions. Burst bias in individual BCNs of BC, VPM, POm, and ZIv. Data is shown for the learning of the initial rule (**a**) and reversed rule (**b**). The burst bias of each unit is shown across various performance categories: inverse ($d' < -0.5$), chance ($-0.5 \leq d' \leq 0.5$), good ($0.5 < d' < 1.65$), and expert ($d' \geq 1.65$). n.s.: $p > 0.05$, *: $p \leq 0.05$, **: $p \leq 0.01$ and ***: $p \leq 0.001$; **a,b**: two-tailed Wilcoxon signed rank test. For detailed description of statistics see Supplementary Data 1. Data pooled from $n = 6$ mice.

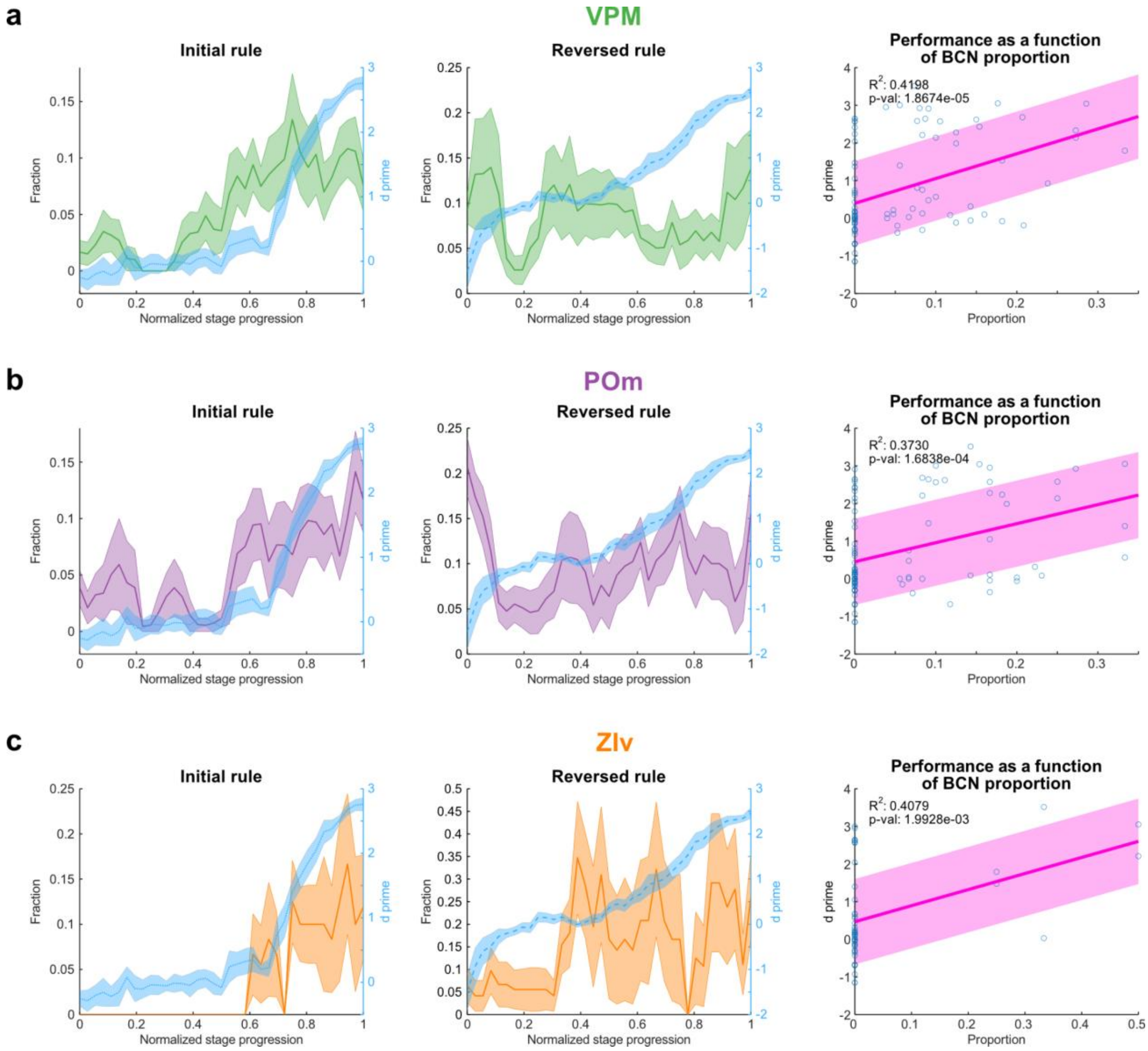

Supplementary Fig. 7 | Progression of BCN proportions with improving performance across brain regions. BCN proportions during initial (left panel) and reversed rules (mid panel) for VPM (**a**), POm (**b**), and ZIv (**c**). Average BCN proportions across animals are shown as a solid line, with SEM as shaded areas. The blue dashed line represents the mean performance (d'). The right panel displays a scatter plot of session performance (d') as a function of BCN proportion, showing a significant positive correlation in all brain regions (VPM: R^2 = 0.4198, p = 1.8674e-05; POm: R^2 = 0.3730, p = 1.6838e-04; ZIv: R^2 = 0.4079, p = 1.9928e-03). Data pooled from n = 6 mice.

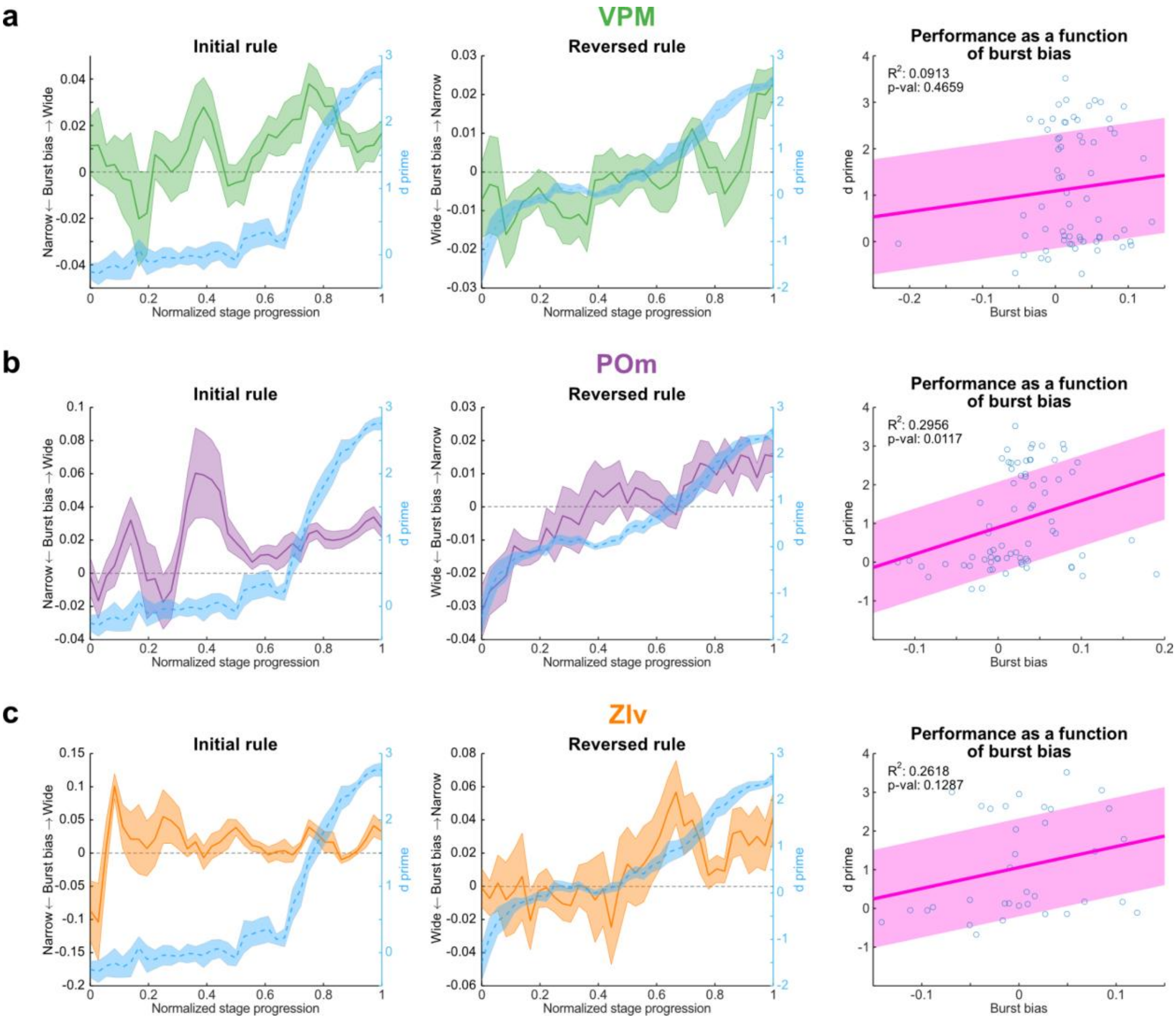

Supplementary Fig. 8 | Progression of burst bias and performance across brain regions. Average burst bias of touch-modulated units during initial (left panel) and reversed rules (mid panel) for VPM (**a**), POm (**b**), and ZIv (**c**). The average burst bias across animals is shown as a solid line, with SEM as shaded areas. The blue dashed line represents the mean performance (d'). The right panel displays a scatter plot of session performance (d') as a function of the burst bias, showing a positive correlation in all brain regions (VPM: $R^2 = 0.0913$, $p = 0.4659$; POm: $R^2 = 0.2956$, $p = 0.0117$; ZIv: $R^2 = 0.2618$, $p = 0.1287$).

Data pooled from n= 6 mice.

a

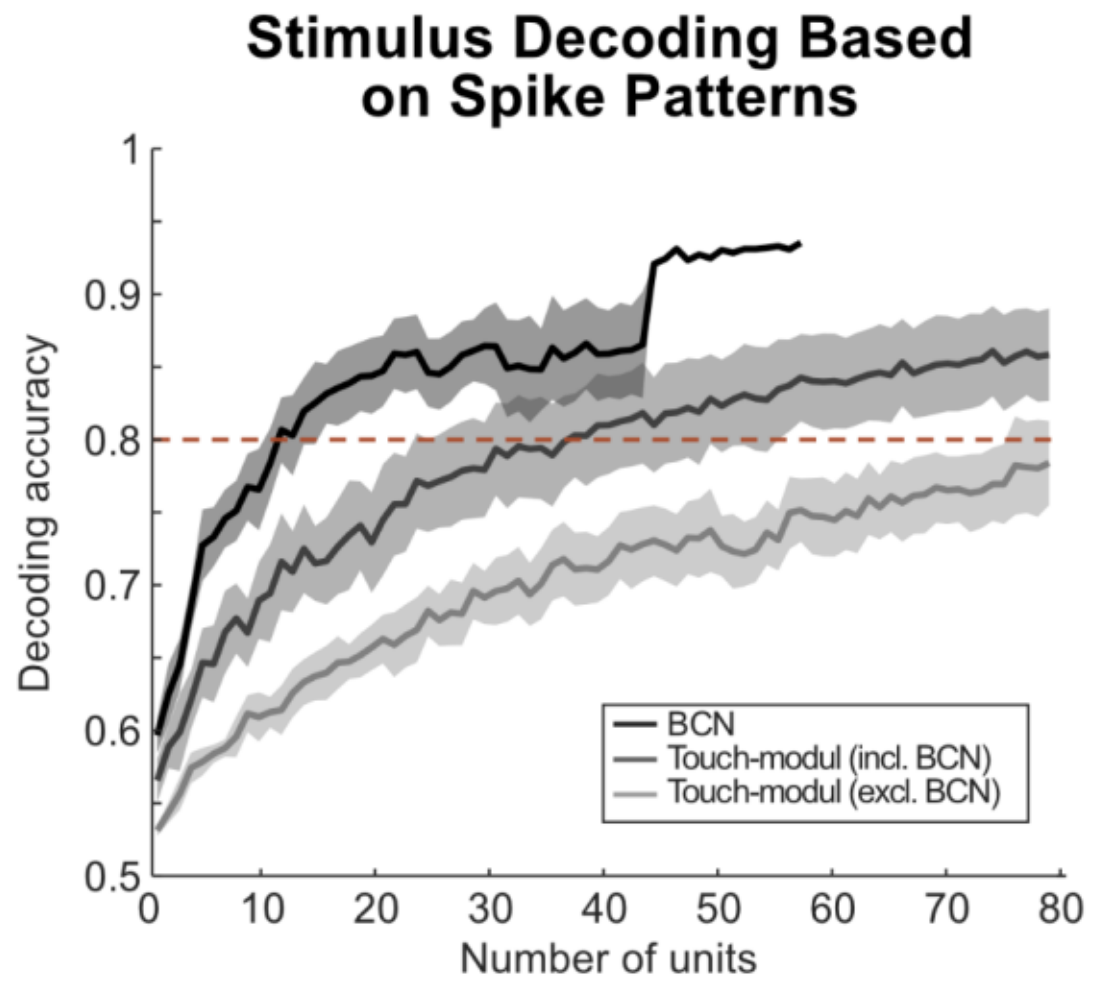

b

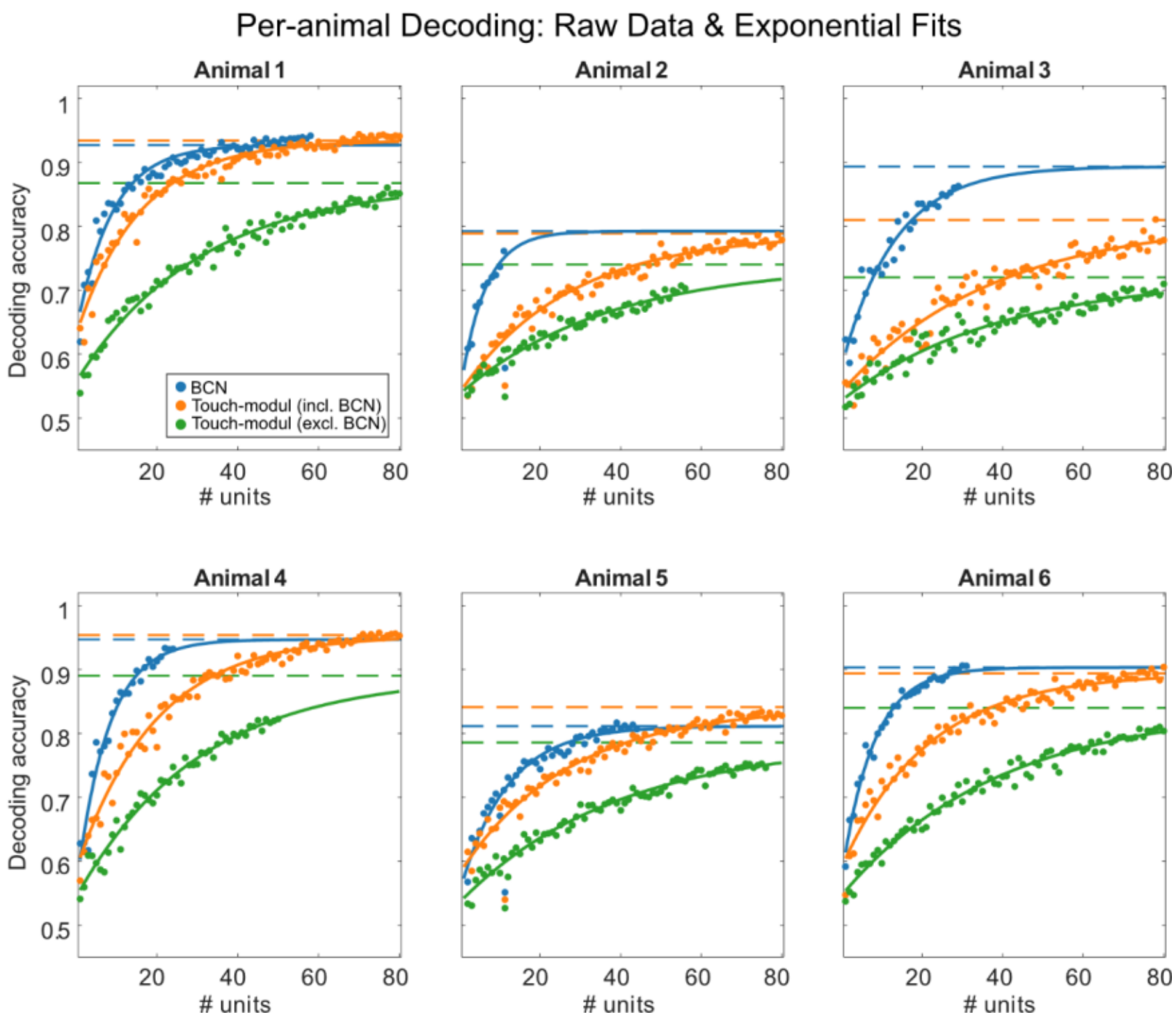

Supplementary Fig. 9 | Animal-wise aperture state decoding reveals enhanced performance of BCNs. a, Decoding accuracy as a function of unit number pooled across all 4 brain areas for classifiers trained on spike patterns from BCNs, all touch-modulated neurons (including BCNs), or touch-modulated neurons excluding BCNs. Analyses were restricted to expert sessions (d′≥1.65) obtained from n = 6 animals. Decoding was performed separately for each animal to account for putative covariant activity among simultaneously recorded units within the same individual, and accuracies were subsequently averaged across animals (SEM as shaded areas). **b,** Decoding accuracy as a function of unit number shown separately for each animal (n = 6), with dots representing the empirical decoding accuracies, whereas solid lines indicate fits of a negative exponential saturation model. Horizontal dashed lines indicate the estimated asymptotic decoding performance for each condition.

Supplementary References

1. Barthó, P. *et al.* Characterization of neocortical principal cells and interneurons by network interactions and extracellular features. *J. Neurophysiol.* **92**, 600–608 (2004).
2. Friedenberger, Z., Harkin, E., Tóth, K. & Naud, R. Silences, spikes and bursts: Three-part knot of the neural code. *J. Physiol.* **601**, 5165–5193 (2023).
