# Supplementary material for: Thalamocortical bursts encode reward contingencies and drive associative learning
Source: Nat Commun. 2026 Aug 11;17:8170. doi: 10.1038/s41467-026-76581-6 (PMC13463074; doi:10.1038/s41467-026-76581-6)
Supplement: Supplementary file 2 [file 41467_2026_76581_MOESM2_ESM.pdf]

Description of Additional Supplementary Files

Supplementary Movie 1: Example correct rejection (CR) trial captured with a high-speed camera (240 FPS, playback slowed by a factor of 10). The mouse successfully identifies the punished aperture and moves away from the lick port. Alongside the video, plots illustrate (1) the average whisker angle relative to the whisker pad (greater angles indicating protraction), (2) the head angle in relation to the midline (values $>0°$ reflect a rightward tilt), and (3) the velocity of the mouse's head within the camera's field of view.

Supplementary Movie 2: Example hit trial captured with a high-speed camera (240 FPS, playback slowed by a factor of 10). The mouse successfully identifies the rewarded aperture and licks the port to receive the reward. Alongside the video, plots illustrate (1) the average whisker angle relative to the whisker pad (greater angles indicating protraction), (2) the head angle in relation to the midline (values $>0°$ reflect a rightward tilt), and (3) the velocity of the mouse's head within the camera's field of view.

Supplementary Movie 3: Wide-angle camera recording (60 FPS, playback accelerated by a factor of 2) providing an overview of the experimental setup. The mouse navigates a linear track, alternating between obtaining rewards and avoiding punishments based on the aperture configuration. The aperture’s state (wide or narrow) changes automatically and at random. Punishment delivery speakers are located behind the track, and high-speed cameras are positioned at the reward sites to capture whisker movements during stimulus interaction.

Supplementary Data 1: Description of statistical parameters.
