# Supplementary material for: Thalamocortical bursts encode reward contingencies and drive associative learning
Source: Nat Commun. 2026 Aug 11;17:8170. doi: 10.1038/s41467-026-76581-6 (PMC13463074; doi:10.1038/s41467-026-76581-6)
Supplement: Supplementary file 3 [file 41467_2026_76581_MOESM7_ESM.pdf]

nature portfolio

Corresponding author(s): Alexander Groh

Last updated by author(s): Aug 2, 2026

Reporting Summary

Nature Portfolio wishes to improve the reproducibility of the work that we publish. This form provides structure for consistency and transparency in reporting. For further information on Nature Portfolio policies, see our Editorial Policies and the Editorial Policy Checklist.

Statistics

For all statistical analyses, confirm that the following items are present in the figure legend, table legend, main text, or Methods section.

n/a	Confirmed	
☐	☒	The exact sample size (*n*) for each experimental group/condition, given as a discrete number and unit of measurement
☐	☒	A statement on whether measurements were taken from distinct samples or whether the same sample was measured repeatedly
☐	☒	The statistical test(s) used AND whether they are one- or two-sided<br>*Only common tests should be described solely by name; describe more complex techniques in the Methods section.*
☒	☐	A description of all covariates tested
☐	☒	A description of any assumptions or corrections, such as tests of normality and adjustment for multiple comparisons
☐	☒	A full description of the statistical parameters including central tendency (e.g. means) or other basic estimates (e.g. regression coefficient) AND variation (e.g. standard deviation) or associated estimates of uncertainty (e.g. confidence intervals)
☐	☒	For null hypothesis testing, the test statistic (e.g. *F*, *t*, *r*) with confidence intervals, effect sizes, degrees of freedom and *P* value noted<br>*Give P values as exact values whenever suitable.*
☒	☐	For Bayesian analysis, information on the choice of priors and Markov chain Monte Carlo settings
☒	☐	For hierarchical and complex designs, identification of the appropriate level for tests and full reporting of outcomes
☒	☐	Estimates of effect sizes (e.g. Cohen's *d*, Pearson's *r*), indicating how they were calculated

Our web collection on statistics for biologists contains articles on many of the points above.

Software and code

Policy information about availability of computer code

Data collection	Data acquisition during behavior was done with Syntalos (Klumpp et al., 2024). An intan-module recorded all analog signals from the lick ports. The overview camera was connected to a recorder module that operated continuously throughout the session. The high-speed cameras and an event list were controlled by a custom-made Python script. The cameras were programmed to start recording when the animal approached the corresponding LP, determined by the crossing of the corresponding light beam. The event list kept track of the triggered light beams and reported the state of the apertures. The list also noted if a trial was a success or failure.
Data analysis	The behavioral data was analyzed using custom code developed in MATLAB R2023a (The MathWorks, Inc.), which is available for reference on GitHub (https://github.com/GrohLab/Thalamocortical-bursts-encode-reward-contingencies-and-drive-associative-learning) and Dryad (https://doi.org/10.5061/dryad.hdr7sqvt1).<br>P-values below 0.05 were considered significant. Mean values were reported with standard deviations and median values with interquartile range (IQR).<br><br>Statistical analysis of the learning process<br>Task performance in each session was measured using the discriminability index d-prime (d'), also known as Fisher discrimination index, as described previously [1,2] defined as the difference between the z-scores of the hit rate and the false alarm rate: d' = z(hit rate)−z(false alarm rate). Due to the constraints of the z-score transformation (which cannot handle proportions of 0 or 1), hit rates and false alarm rates of 0 are typically adjusted to (12 * n) and rates of 1 are adjusted to (1-12 * n), with n being the number of go (for hit rate adjustment), or no-go trials (for false alarm rate adjustment), respectively. A higher d' indicates greater discriminability, and thus a greater discrimination performance of the mice. A d' of 0 describes a discrimination performance at chance level. The criterion for achieving expert performance was established at a d-prime of 1.65, corresponding to a one-tailed significance level of α = 0.05.

Extraction of behavioral data from videos
Spatial information of the mice was extracted from video recordings using DeepLabCut [3]. Following preprocessing with median background image subtraction, the DeepLabCut model was trained on a subset of manually annotated video frames. For the high-speed videos, these markers consisted of eight data points for two whiskers on each side from whisker base to tip, and markers for the mouse's head contours. In overview videos, spatial positioning and velocity were estimated, based on anatomical landmarks on the mouse's body and head.

Electrophysiological data
The raw electrophysiological data was spike sorted with KiloSort 2.0. Subsequently, the sorted clusters were curated automatically using the Ecephys spike sorting algorithm provided by the Allen Brain Observatory (code available at https://github.com/alleninstitute/ecephys_spike_sorting). The algorithm includes noise templates to identify and exclude clusters exhibiting characteristics indicative of noise, such as irregular waveform shapes and inter-spike interval (ISI) histograms. The following quality metrics were then applied to further exclude any clusters indicative of multi-unit activity: isolation distance (computed from Mahalanobis distance) greater than 15 and ISI violation below 3%, with an ISI threshold of 1.5 milliseconds. The criteria were selected on the basis of previous studies [4–6], where they have been demonstrated to effectively distinguish well-isolated single units from noise and multi-unit activity. The individual units were further classified into fast spiking (FS) and regular spiking (RS) units, putatively representing interneurons and excitatory neurons, respectively [7]. The threshold for differentiating RS from FS units was set to a trough-to-peak duration of 350 µs for cortex and zona incerta, and 300 µs for thalamic nuclei (Extended Data Fig. 2). These thresholds align with previous studies [8,9] and consider the fact that extracellular waveforms in the thalamus are shorter than those in the cortex [8,9]. For further analysis, only RS units were selected for BC, VPM, and POm, while FS/RS units were selected for ZIv because of limited data in the literature in which FS/RS units in the ZI were associated with specific cell types (i.e., inhibitory / excitatory).

Neuropixels data analysis
Neuropixels recordings were done using a OneBox (Imec). Data was acquired using SpikeGLX (https://github.com/billkarsh/SpikeGLX) at 30kHz. Data from pharmacological manipulations was concatenated and processed using CatGT (https://github.com/billkarsh/CatGT). Further processing was done using SpikeInterface [10] and Kilosort [4,11], followed by manual curation in Phy (https://github.com/cortex-lab/phy). We pooled data from POm and VPM and used only RS units for further analysis.

Neural response metrics
Whisker-touch and locomotion responsiveness: Whisker touch responsiveness was defined by a significant increase in firing rates upon aperture touch within a 200 ms response window following aperture touch (to either of the two aperture states) relative to the baseline window (600 to 400 ms before touch), analyzed using a two-sided Wilcoxon signed rank test. Locomotion-encoding units were identified through stepwise multiple linear regression analysis. This method is used to identify the significant predictors of a dependent variable by iteratively removing the least significant variables until all remaining variables are significant [12].

Bursts: Bursts are characterized as sequences of two or more spikes with anISI of ≤ 10 ms and a tail ISI of > 15 ms, in accordance with the criteria established previously [13,14].

Burstiness: To compute the burstiness per unit, burst spikes were first labelled using the above criteria. Then, burstiness is the fraction of burst spikes among all spikes in the overall analysis window (for example, the whole recording after drug injection).

Burst index: The burst index (BI) is calculated as the difference between the proportion of burst spikes (Pbsp) in the response window (0 to 200 ms after touch) relative to the baseline window (600 to 400 ms before touch). Pbsp is defined as the number of burst spikes (Nbsp) over all spikes (Nsp) in a given window. Burst indices can range between -1 (maximal burst proportion in baseline with minimal burst proportion in response window) and 1. The formula is given by: BI = Pbsp,response-Pbsp,baseline = Nbsp,responseNsp,response - Nbsp,baselineNsp,baseline

Burst bias: The burst bias (BB) is derived from the burst indices of two different aperture conditions (BI1and BI2). It is calculated as the difference between the two burst indices, divided by two. Values can range between -1 (maximal burst bias for aperture 2) and 1 (maximal burst bias for aperture 1). The formula is given by: BB=BI1−BI22

A significant burst bias classifies the unit as a burst-coding neuron (BCN), as determined by a Wilcoxon rank sum test.

Burst fraction: The burst fraction (fb) is defined as the number of burst events (Nbe), disregarding the number of spikes within bursts, divided by the total event rate (Ne), which includes both tonic spikes (Ntsp) and burst events, as characterized in Friedenberger et al. (2023) [15]. The formula is given by: fb = NbeNe = NbeNbe + Ntsp

Tonic index: The tonic index (TI) is calculated as the quotient of the number of tonic spikes (Ntsp) in the response window (0 to 200 ms after touch) divided by the overall number of tonic spikes in the response and baseline windows (600 to 400 ms before touch) combined. Values can range between 0 (only tonic spiking in the baseline window) and 1 (only tonic spiking in the response window). The formula is given by: TI = Ntsp,responseNtsp,response + Ntsp,baseline

Tonic bias: The tonic bias (TB) is derived from the tonic indices of two different aperture conditions (TI1 and TI2). It is calculated as the difference between the two tonic indices. Values can range between -1 (maximal tonic bias for condition 2) and 1 (maximal tonic bias for condition 1). The formula is given by: TI =TI1 - TI2

A significant tonic bias classifies the unit as a tonic-coding neuron (TCN), as determined by a Wilcoxon rank sum test.

Neural decoding analysis
The decoding of aperture states from neural spike time data was performed using the Neural Decoding Toolbox [16]. Classifier training was performed with an increasing number of units of either solely BCNs, touch-modulated units including BCNs, or touch-modulated units excluding BCNs. For each configuration, the dataset was divided into training (90% of labels) and test set (10% of labels). When insufficient units were available for partitioning, the test set was selected from the remaining units. Each set contained the spike traces of individual neurons for a given trial and the corresponding aperture labels. A support vector machine (SVM) classifier was trained using the LIBSVM software package. The classifier underwent 10-fold cross-validation, involving separate training and testing across different data partitions. To assess variability in decoding accuracy, each unit selection was bootstrapped 20 times. The mean decoding accuracy was calculated across a time window from trigger onset to 400 ms post-trigger onset.

1. Stanislaw, H. & Todorov, N. Calculation of signal detection theory measures. Behav. Res. Methods Instrum. Comput. 31, 137–149 (1999).
2. Macmillan, N. A. Detection Theory: A User's Guide. (Cambridge University Press, Cambridge, England, 1991).

3. Mathis, A. et al. DeepLabCut: markerless pose estimation of user-defined body parts with deep learning. Nat. Neurosci. 21, 1281–1289 (2018).
4. Onodera, K. & Kato, H. K. Translaminar recurrence from layer 5 suppresses superficial cortical layers. Nat. Commun. 13, 2585 (2022).
5. Rosenberg, D. M. & Horn, C. C. Neurophysiological analytics for all! Free open-source software tools for documenting, analyzing, visualizing, and sharing using electronic notebooks. J. Neurophysiol. 116, 252–262 (2016).
6. Trautmann, E. M. et al. Accurate estimation of neural population dynamics without spike sorting. Neuron 103, 292–308.e4 (2019).
7. Barthó, P. et al. Characterization of neocortical principal cells and interneurons by network interactions and extracellular features. J. Neurophysiol. 92, 600–608 (2004).
8. Mahrach, A., Chen, G., Li, N., van Vreeswijk, C. & Hansel, D. Mechanisms underlying the response of mouse cortical networks to optogenetic manipulation. Elife 9, (2020).
9. Barthó, P. et al. Ongoing network state controls the length of sleep spindles via inhibitory activity. Neuron 82, 1367–1379 (2014).
10. Buccino, A. P. et al. SpikeInterface, a unified framework for spike sorting. Elife 9, e61834 (2020).
11. Pachitariu, M., Sridhar, S., Pennington, J. & Stringer, C. Spike sorting with Kilosort4. Nat. Methods 21, 914–921 (2024).
12. Sun, W. et al. Context value updating and multidimensional neuronal encoding in the retrosplenial cortex. Nat. Commun. 12, 6045 (2021).
13. Urbain, N. et al. Whisking-related changes in neuronal firing and membrane potential dynamics in the somatosensory thalamus of awake mice. Cell Rep. 13, 647–656 (2015).
14. de Kock, C. P. J. & Sakmann, B. High frequency action potential bursts (>or= 100 Hz) in L2/3 and L5B thick tufted neurons in anaesthetized and awake rat primary somatosensory cortex: High frequency bursts in barrel cortex. J. Physiol. 586, 3353–3364 (2008).
15. Friedenberger, Z., Harkin, E., Tóth, K. & Naud, R. Silences, spikes and bursts: Three-part knot of the neural code. J. Physiol. 601, 5165–5193 (2023).
16. Meyers, E. M. The neural decoding toolbox. Front. Neuroinform. 7, 8 (2013).

For manuscripts utilizing custom algorithms or software that are central to the research but not yet described in published literature, software must be made available to editors and reviewers. We strongly encourage code deposition in a community repository (e.g. GitHub). See the Nature Portfolio guidelines for submitting code & software for further information.

Data

Policy information about availability of data

All manuscripts must include a data availability statement. This statement should provide the following information, where applicable:

- Accession codes, unique identifiers, or web links for publicly available datasets
- A description of any restrictions on data availability
- For clinical datasets or third party data, please ensure that the statement adheres to our policy

ll the data are in the manuscript or in supplementary material. Source data and code ist available under the following link: https://doi.org/10.5061/dryad.hdr7sqvt1

Research involving human participants, their data, or biological material

Policy information about studies with human participants or human data. See also policy information about sex, gender (identity/presentation), and sexual orientation and race, ethnicity and racism.

Reporting on sex and gender	*Use the terms sex (biological attribute) and gender (shaped by social and cultural circumstances) carefully in order to avoid confusing both terms. Indicate if findings apply to only one sex or gender; describe whether sex and gender were considered in study design; whether sex and/or gender was determined based on self-reporting or assigned and methods used. Provide in the source data disaggregated sex and gender data, where this information has been collected, and if consent has been obtained for sharing of individual-level data; provide overall numbers in this Reporting Summary. Please state if this information has not been collected. Report sex- and gender-based analyses where performed, justify reasons for lack of sex- and gender-based analysis.*
Reporting on race, ethnicity, or other socially relevant groupings	*Please specify the socially constructed or socially relevant categorization variable(s) used in your manuscript and explain why they were used. Please note that such variables should not be used as proxies for other socially constructed/relevant variables (for example, race or ethnicity should not be used as a proxy for socioeconomic status). Provide clear definitions of the relevant terms used, how they were provided (by the participants/respondents, the researchers, or third parties), and the method(s) used to classify people into the different categories (e.g. self-report, census or administrative data, social media data, etc.) Please provide details about how you controlled for confounding variables in your analyses.*
Population characteristics	*Describe the covariate-relevant population characteristics of the human research participants (e.g. age, genotypic information, past and current diagnosis and treatment categories). If you filled out the behavioural & social sciences study design questions and have nothing to add here, write "See above."*
Recruitment	*Describe how participants were recruited. Outline any potential self-selection bias or other biases that may be present and how these are likely to impact results.*
Ethics oversight	*Identify the organization(s) that approved the study protocol.*

Note that full information on the approval of the study protocol must also be provided in the manuscript.

Field-specific reporting

Please select the one below that is the best fit for your research. If you are not sure, read the appropriate sections before making your selection.

- [x] Life sciences
- [ ] Behavioural & social sciences
- [ ] Ecological, evolutionary & environmental sciences

For a reference copy of the document with all sections, see nature.com/documents/nr-reporting-summary-flat.pdf

Life sciences study design

All studies must disclose on these points even when the disclosure is negative.

Sample size

Sample sizes were chosen in accordance with standard numbers of animals in comparable studies in the field. All experiments supporting the conclusions of this paper had a sample size of at least 6 mice. CaV3.1 knock-down experiments with scrambled shRNA injections as controls were replicated in 5 animals for each group respectively. Burst suppression via Z944 with DMSO controls were replicated in 4 animals for each group respectively.

The exact size of specific experimental groups are given in the figures legends, results and methods.

Data exclusions

Tetrode tracks were manually reconstructed from the microscope images using the Amira software (v6.5, Thermo Fisher Scientific, Waltham, MA). Subsequently, all tetrodes located outside of the designated target areas were excluded from further analysis.

The raw electrophysiological data was spike-sorted with KiloSort 2.0. Subsequently, the sorted clusters were curated automatically using the Ecephys spike sorting algorithm provided by the Allen Brain Observatory (code available at https://github.com/alleninstitute/ecephys_spike_sorting). The algorithm includes noise templates to identify and exclude clusters exhibiting characteristics indicative of noise, such as irregular waveform shapes and interspike interval (ISI) histograms. The following quality metrics were then applied to further exclude any clusters indicative of multi-unit activity: isolation distance (computed from Mahalanobis distance) greater than 15 and ISI violation below 3 %, with an ISI threshold of 1.5 milliseconds. The criteria were selected based on previous studies (Onodera & Kato, 2022; Rosenberg & Horn, 2016; Trautmann et al., 2019), where they have been demonstrated to effectively distinguish well-isolated single units from noise and multi-unit activity. The individual units were further classified into fast spiking (FS) and regular spiking (RS) units, putatively representing inhibitory and excitatory neurons, respectively (Barthó et al., 2004). The threshold for differentiating RS from FS units was set to a trough-to-peak duration of 350 µs for cortex and zona incerta, and 300 µs for thalamic nuclei. These thresholds align with previous studies (Barthó et al., 2014; Mahrach et al., 2020) and consider the fact that extracellular waveforms in the thalamus are shorter than those in the cortex (Barthó et al., 2014). For further analysis, only RS units were selected for BC, VPM, and POm, while FS units were selected for ZIv.

Replication

Behavioral experiments were replicated in 6 mice. CaV3.1 knock-down experiments with scrambled shRNA injections as controls were replicated in 5 animals for each group respectively. Burst suppression via Z944 with DMSO controls were replicated in 4 animals for each group respectively.

Randomization

Each experiment used a cohort of litter-mates in which all mice were exposed to the same conditions and underwent the same behavioral stages. Mice were randomly assigned to groups based on their mouse IDs, which were randomly assigned at the start.

Blinding

Recordings and analysis were run automatically by a computer with predefined parameters, which removes any subjective components of the data collection. For the manipulations (Z944, shRNA) the experimenter was blinded during the entire experiments.

Reporting for specific materials, systems and methods

We require information from authors about some types of materials, experimental systems and methods used in many studies. Here, indicate whether each material, system or method listed is relevant to your study. If you are not sure if a list item applies to your research, read the appropriate section before selecting a response.

Materials & experimental systems

n/a	Involved in the study	
[ ]	[x]	Antibodies
[x]	[ ]	Eukaryotic cell lines
[x]	[ ]	Palaeontology and archaeology
[ ]	[x]	Animals and other organisms
[x]	[ ]	Clinical data
[x]	[ ]	Dual use research of concern
[x]	[ ]	Plants

Methods

n/a	Involved in the study	
[x]	[ ]	ChIP-seq
[x]	[ ]	Flow cytometry
[x]	[ ]	MRI-based neuroimaging

Antibodies

Antibodies used

antibody against the CaV3.1 channel (Anti-CACNA1G (CaV3.1) Antibody, #ACC-021, Alomone Labs, Jerusalem 9104201, Israel), diluted 1 : 200 in phosphate buffer (as in Latchoumane et al., 2025)

Validation	Charles-francois V Latchoumane, Joon-Hyuk Lee, Seong-Wook Kim, Jinhyun Kim, Hee-Sup Shin (2025) Mediodorsal thalamic nucleus mediates resistance to ethanol through Cav3.1 T-type Ca2+ regulation of neural activity eLife 13:RP93200 https://doi.org/10.7554/eLife.93200.3

Animals and other research organisms

Policy information about studies involving animals; ARRIVE guidelines recommended for reporting animal research, and Sex and Gender in Research

Laboratory animals	The study involved adult male C57BL/6NRj mice (Janvier Labs, Le Genest-Saint-Isle, France) aged 8-10 weeks at the onset of training. The mice were individually housed in a ventilated Scantainer (Scantainer Classic, SCANBUR A/S, Karlslunde, Denmark) maintained on a 12-hour inverted light/dark cycle (lights off at 7:00 a.m. and on at 7:00 p.m.) with controlled temperature (20-24 °C) and humidity (45-65%). During behavioral training, which was conducted during day time, mice were subjected to a food control regimen, maintaining their body weight at 85-95% of their initial weight, with ad libitum access to water.
Wild animals	No wild animals were used in this study.
Reporting on sex	The experiments were done with adult male mice. Sex-specific effects were not addressed in this study.
Field-collected samples	No field-collected samples were used in this study.
Ethics oversight	All experimental procedures received approval from the local governing authority (Regierungspräsidium Karlsruhe, Germany, approval numbers: 35-9185.81/G-216/19 and 35-9185.81/G-145/24) and were conducted in accordance with their ethical guidelines.

Note that full information on the approval of the study protocol must also be provided in the manuscript.

Plants

Seed stocks	*Report on the source of all seed stocks or other plant material used. If applicable, state the seed stock centre and catalogue number. If plant specimens were collected from the field, describe the collection location, date and sampling procedures.*
Novel plant genotypes	*Describe the methods by which all novel plant genotypes were produced. This includes those generated by transgenic approaches, gene editing, chemical/radiation-based mutagenesis and hybridization. For transgenic lines, describe the transformation method, the number of independent lines analyzed and the generation upon which experiments were performed. For gene-edited lines, describe the editor used, the endogenous sequence targeted for editing, the targeting guide RNA sequence (if applicable) and how the editor was applied.*
Authentication	*Describe any authentication procedures for each seed stock used or novel genotype generated. Describe any experiments used to assess the effect of a mutation and, where applicable, how potential secondary effects (e.g. second site T-DNA insertions, mosiacism, off-target gene editing) were examined.*
