# Supplementary material for: Thalamocortical bursts encode reward contingencies and drive associative learning
Source: Nat Commun. 2026 Aug 11;17:8170. doi: 10.1038/s41467-026-76581-6 (PMC13463074; doi:10.1038/s41467-026-76581-6)
Supplement: Supplementary file 4 [file 41467_2026_76581_MOESM8_ESM.pdf]

nature portfolio

Peer Review File

Thalamocortical bursts encode reward contingencies and drive associative learning

Corresponding Author: Dr Alexander Groh

This file contains all reviewer reports in order by version, followed by all author rebuttals in order by version.

Version 0:

Reviewer comments:

Reviewer #1

(Remarks to the Author)
This manuscript presents experiments and analyses designed to track burst coding from cortex and thalamus during an ethological learning task. Establishing the role and preponderance of burst coding within the central nervous system has been an important question in system neuroscience, one thought to be a key to understanding learning and attention. The data presented in this paper show novel phenomena that bring an important advance in this question. I thought it was an exciting article and, assuming that the concerns I raise below can be addressed, I would see it well fitted to Nature Communications.
Before stating my concerns, I would like to highlight the pieces I find particularly compelling in the current manuscript. One is the report of fast changes in burst fraction in cells that do not show concomitant changes in tonic firing. Similar fast changes in burst fraction were reported in some areas of thalamus and by Naud et al. eLife (2024) for cortex but for artificial stimuli. I was not aware of such a clear proof of burst multiplexing in cortex as provided by the current manuscript. Another discovery is that of the malleability of these burst responses with reward contingencies. A malleability that is nowhere near that of tonic firing responses (Fig. 3b). Then there is the tracking of such burst response bias throughout learning, showing that burst biases appears slightly before the time where more cells start to encode touches (Fig. 3d vs Fig. 1a), suggesting that whatever this bursting means, it precedes the bulk of the changes in representation. And lastly there are two perturbation experiments that help disambiguate the causal role played by the mechanisms underlying these bursts, although the findings there are less striking. Together, this amounts to a significant advance in our understanding.
My concerns below mainly relate to the final interpretation of these data as presented in the discussion-abstract and to some missing methodological details and their interpretation.
1. Teaching signals and representations. Abstract, intro and discussion mention teaching signals, which were hypothesized to be encoded by bursts in Payeur et al. among others. While this is a great way to justify the importance of the study, I find the evidence, while very interesting, does not support well that the bursting observed are learning signals. At least not in the sense used by Payeur et al. where bursting encoded features of perceptual errors. I would like to note that the evidence presented does not disprove bursting as teaching signals either.
While there is unknown degree of perceptual error in the task studied by Heimburg et al., two piece of evidence are at odds with Payeur's perceptual error idea. One is that the burst bias seems to mostly increase during learning. If burst biases encoded an error, this error should start to reduce when the behavior starts to stabilize. The data on this is ambiguous (Fig. 4d). Then it seems to me that the neutral stimuli should elicit more perceptual error as to whether it is narrow or wide, so more variance in bursting, again ambiguous (Fig 3e). What does fit with Payeur et al. is that these signals occur before the neurons change their selectivities.
Another model that can account for these data is simply a reward contingency. For instance a Q or state value in reinforcement learning would account well for the data in the sense that it should increase with learning the reward association. It seems to me that a reward prediction error (which would be the learning signal in a reinforcement learning context, affecting the Q or state values) does not match well with the data as again, as for perceptual errors, RPE should go down when the task is well mastered and the derivative in d' goes to zero.
Together, I think that if the authors want to make claims about these being learning signals, they should anchor this claim in the right phenomenology. Perhaps it would be safer given the ambiguity of the data to refrain from these claims and only discuss the possible parrallels.
2. Teaching signals and perturbation. The perturbation of CaV KD and Z944 show a slowing down of learning and a reduction of performance in expert animals. In my understanding, abolishing a teaching signal should affect learning phase and not the performance in expert animals. The data presented would be more consistent with a model where the bursting

mechanisms affect the perception, for instance via an attention mechanism. If the bursting signals relate to attention or perception, perturbing them should also slow down learning. The current conclusions of this section seems misplaced to me.
3. Definition of BCN and TCN neurons. I could not find a precise definition of BCN and TCN. Granted BCN are neurons that show a response in burst fraction, and TCN are neurons that show a response in tonic firing. What are the constraints for the the cross modalities? Are BCN required to have no change in tonic firing and TCN required to have no changes in burst fraction? In which case, the 'not coding neurons' contain neurons that respond well in both burst fraction and tonic firing? Otherwise, it could be that the BCN are selected first, such that they contain cells that respond both positively and negatively in terms of tonic firing; then TCN are made of only neurons that do not change in burst fraction and NCN are really non-coding. Regardless, I think the statistical comparisons in Fig. 2c should not be shown since they reflect the definition of BCN and TCN, unless I am missing something. (Note that the fact that BCN have no tonic firing changes implies that these neurons cannot be accounted by a Poisson model, or a model where the firing rate is simply high in some neurons that inherit reward contingencies, I thought this was an important point but up to the authors).
Most neuroscientist that looks at this type of separation would have the impression that the authors have separated the neurons that fire strongly (BCN) from those that fire weakly (TCN). In order to clarify that this is not the case, one could make a surrogate model where the response spike times are shuffled across cells but within the time bin (function of time to touch). In this way, one would more explicitly separate the burst code from the firing rate code.

Smaller technical points.
- What happens in the calculation of burst fraction when there are no spikes (zero denominator)?
- It seems to me that the burst rates reported are very high for sorted units. Fig. 3a reports burst rates of 30 Hz. This seems an order of magnitude too large for cortex. But not too bad for thalamus. So it could be that this number is heavily weighted by thalamus.
- A error signal should come more in the reverse scenario and with some delay to account for the time to realise there has been an error. I wonder if the authors analyzed this. Fine if not.
- The claim in the discussion about the linear burst fraction in Payeur et al. seems off to me. I did not fully understand what the authors meant. Payeur et al. needed to linearize the input-output function, which is different from the linear progression.

(Remarks on code availability)
I have not looked at the code.

Reviewer #2

(Remarks to the Author)
The manuscript by Heimburg et al. investigates the role of action potential (AP) bursts in thalamocortical circuits during associative learning. Using high-density electrophysiological recordings combined with a whisker-based go/no-go paradigm in freely moving mice (recently developed by the same group), the authors recorded neuronal activity across cortical and thalamic nuclei as animals learned the task. They report that burst firing becomes increasingly prevalent across recorded regions and correlates with behavioral performance over time. Furthermore, burst events appear to encode contextual information about the stimulus, specifically whether it predicts reward. Pharmacological and genetic manipulations that suppress bursting activity in thalamic nuclei disrupted both learning and task performance. Based on these findings, the authors propose that neuronal bursts function as context-sensitive teaching signals that drive associative learning.
Overall, the study addresses an interesting and timely question in the field. The experiments are well executed, and the manipulation experiments are particularly valuable. The analyses are mostly appropriate. However, I find that the central claim is not yet fully supported by the presented evidence. Addressing the concerns detailed below would strengthen the manuscript and enhance the clarity and impact of the study.

Major comments:
1. The central claim of this study is that thalamocortical bursts act as context-sensitive teaching signals that drive associative learning. However, the results presented here more convincingly demonstrate a relationship between thalamocortical bursts and behavioral context (rewarded vs. non-rewarded) or lick execution, rather than learning per se. For example, burst activity shows a strong correlation with the animal's decision to lick (Fig. 3f), and pharmacological suppression of thalamic bursting impaired not only learning but also task performance (Fig. 6f). This raises an important concern: if bursting directly affects sensory perception or behavioral execution, it becomes difficult to disentangle whether bursts specifically contribute to learning processes or instead reflect or modulate performance-related signals. The current experiments do not clearly isolate these possibilities.
Furthermore, the concept of "teaching signal" remains insufficiently defined throughout the manuscript. It is unclear whether the authors propose that bursts instruct downstream circuits to develop task-relevant activity patterns during learning and facilitate updating of representations when task contingencies change. In addition, it is unclear how such teaching signals are expected to evolve with expertise. For example, if bursts serve as teaching signals, should their magnitude decrease once animals reach stable performance, or persist to maintain learned representations? A precise definition of a teaching signal, along with experimental evidence that distinguishes it from performance-related signals, is necessary to support the central claim of this study.

2. Touch-evoked bursting activity increases as animals learn the task and is sensitive to behavioral context (rewarded vs. non-rewarded trials). This modulation is particularly apparent in the barrel cortex, which is frequently highlighted in representative traces and plots, and to a similar or somewhat lesser extent in thalamic regions (Extended Data Figs. 4, 7, 8). The manipulation experiments presented in Fig. 6 provide valuable insight into the role of burst activity in behavior. However, these manipulations are restricted to thalamic bursts and do not address the potential contribution of cortical burst

activity. Does this focus reflect a specific mechanistic hypothesis?
Given the prominent modulation of bursting observed in the cortex, assessing the causal contribution of cortical burst activity would substantially strengthen the study and help determine whether bursting plays distinct or complementary roles across thalamic and cortical circuits.

3. Given its formulation, the burst bias (BB) does not distinguish between qualitatively different coding scenarios. For example, BB would be positive both when (1) the burst index (BI) is > 0 for the wide-aperture with BI ~ 0 in the narrow-aperture, and when (2) BI is ~ 0 for the wide-aperture but < 0 for the narrow-aperture. These scenarios reflect distinct coding strategies, yet they yield similar BB values. Distinguishing between burst enhancement and burst suppression would improve the interpretation of how burst activity encodes stimulus or contextual information.
Relatedly, it would be helpful to clarify whether some neurons encode stimulus or contextual information through reductions in burst activity rather than increases.

4. If I understood correctly from the text and statistical summary, the decoding analyses (Fig. 5) appear to have been performed using pooled single-unit data from multiple neurons recorded across six mice. Pooling units across animals does not account for inter-trial covariance among neurons recorded within the same individual animal. For example, neurons recorded simultaneously within the same animal often exhibit trial-to-trial correlated variability due to shared network or behavioral state fluctuations. Such covariance can contribute to population coding. Pooling neurons across animals disrupts these correlations because neurons from different animals cannot share trial-by-trial variability, potentially distorting decoding performance and its interpretation. Decoding analyses performed on neuronal populations within each animal, then followed by comparisons or averaging across animals, would provide a more appropriate and statistically rigorous assessment.

5. The authors claim that “un-learning” diminishes burst biases (Line 366). However, based on the presented plots in Fig. 3c, it appears that burst biases segregate into two subpopulations, one with BB > 0 and the other with BB < 0, such that the population average approaches zero, rather than individual neurons converging toward zero bias. If this interpretation is correct, the conclusion that un-learning reduces burst bias at the single-neuron level may need to be reconsidered or clarified.
Relatedly, it would be helpful to examine behavioral performance metrics during the un-learning phase. Although Fig. 1d shows that d’ approaches zero during “contingency degradation”, it remains unclear whether this reflects balanced hit and false alarm rates or a general disengagement from the task. Distinguishing between these possibilities may help interpretation of the persistence of neuronal subpopulations with BB > 0 and BB < 0 during un-learning.

6. The manuscript does not provide measurements of whisker behavior (e.g., number of whisker-aperture contacts, whisking amplitude, etc). These variables are important to support the claim that burst activity encodes contextual information, rather than simply reflecting differences in sensory input or motor output between go and no-go stimuli. Without quantifying whisker contacts and movement parameters, it remains difficult to exclude the possibility that differences in burst activity arise from variations in tactile sampling or whisking strategies across conditions.

Minor comments:
1. Line 42 “… and has so far mainly been attributed to hippocampal circuits18.19.” Based on the cited references, I believe that the authors are referring to hippocampal sharp-wave ripples. However, sharp-wave ripples are typically characterized as population-level burst events, reflecting synchronized activity across neuronal ensembles, which differs from single-cell burst firing, the primary focus of the present study. Clarifying this distinction would help avoid conceptual confusion and would better position the current study relative to prior literature.

2. Fig. 2a: The definition and calculation of “Learning progression” on the x-axis should be provided.

3. Fig. 4d, middle panel: the y-axis is labeled “wide <-> narrow,” while in Extended Data Fig. 8 the y-axis is labeled “narrow <-> wide.” This inconsistency is confusing.

4. Extended Data Fig. 5, right panels: The title of the y-axis should be “tonic preference”.

5. Fig. 4c,e and Extended Data Fig. 7 and 8, right panels: The statistical summary table states “Correlation of performance … in expert sessions.” If this is the case, it is unclear why the plots include data points with d’ values close to zero.

(Remarks on code availability)

Reviewer #3

(Remarks to the Author)
IIn their paper ‘Thalamocortical bursts encode reward contingencies and drive
associative learning‘ Heimburg et al describe single-cell activity and burst-firing patterns in the rodent somatosensory corticothalamic system during an aperture discrimination task. The authors observe a strong correlation of burst-firing and reward-contigencies, i.e., neurons burst in response to the rewarded aperture. The authors show with rule reversal experiments that these responses are indeed contingent on reward and not specific sensory cues. Finally, the authors use an iRNA approach to interfere with bursting and find effects on learning. I think this is a paper of considerable significance, because the core finding is novel and potentially very important.

Major:

1. Reward-contingent burst-firing
The observation of reward-contingent burst-firing is extraordinary. The reasons for this assessment are the following: (i) We have had many hypotheses about burst firing in the past (thalamic tonic firing vs bursting in sleep vs wakefulness, packaging of information etc etc), but this observation is different. (ii) The evidence presented by the authors is very clear. (iii) What adds to the significance of the finding is that the reward-contingent burst-firing seems to work across structures (thalamus and cortex). This is very unexpected, because investigators generally proposed very different explanations for bursting in thalamus (low-threshold Ca-spikes) and cortex (here cell-type specific explanations predominate, i.e., intrinsic bursting of layer 5 pyramids, dendritic burst mechanisms and the like. This is not, what the authors observe, however. (iv) Finally, reward coding is immensely important in the brain.

2. Last sentence of the abstract
In the last sentence of the abstract the authors link their burst coding results to memory formation. My impression is that this link stretches the results somewhat. Sticking to reward contingency is the more proximal and better substantiated interpretation here.

Minor:

1. It would be great to see some additional information about the cortical neurons recorded here (layers etc).
3. The authors target a T-type Ca-channel to affect bursting. Is this a plausible candidate for reward-contingent burst-firing across the somatosensory system? Please comment.
4. How could reward and bursting be linked cellularly? What are the reward related signals that get into corticothalamic system? Is there an effect of dopamine application on thalamocortical bursting.
5. The authors might want to consider the apical tuft data reported by Benezra from the Bruno lab.

(Remarks on code availability)

Version 1:

Reviewer comments:

Reviewer #1

(Remarks to the Author)
The authors have addressed all my concerns. I am particularly glad that they have re-analyzed their data to separate the learning vs perception conflation. The new analyses are convincing.

(Remarks on code availability)

Reviewer #2

(Remarks to the Author)
The authors have carefully addressed the concerns raised by me and the other reviewers. I thank them for their thoughtful responses, and I believe the manuscript has been significantly improved. However, I have one remaining point regarding the interpretation of the reduced burst bias during the degradation stage.
In their response to my comment #5, the authors acknowledge that this reduction likely reflects two factors: 1) a genuine attenuation of aperture-selective burst coding among individual neurons, and 2) a shift in the relative proportions of narrow- and wide-encoding BCNs, which become more balanced during degradation. However, this dual interpretation is not fully reflected in the Results and Discussion. The text mainly emphasizes that burst coding is “largely abolished” or “largely diminishes” during this stage, while overlooking the contribution of the balanced coexistence of narrow- and wide-encoding BCNs.
This point is relevant to the authors’ interpretation of the animals’ behavior during degradation. The authors note that mice showed “a strong tendency to lick regardless of trial type … with hit rates remaining near ceiling and correct rejection rates close to zero, consistent with poor stimulus discrimination”. This behavioral pattern suggests that, rather than simply undergoing “un-learning”, mice may have formed a new reward contingency or adopted a new “response strategy”, in which they treated both wide- and narrow-aperture stimuli as reward-predictive. This interpretation aligns well with the observed balanced coexistence of narrow- and wide-encoding BCNs during degradation.
I suggest that the authors reflect these points in the Results and/or Discussion. This clarification would help readers better interpret the data and further strengthen the manuscript.

(Remarks on code availability)

Reviewer #3

(Remarks to the Author)
The authors did a thorough revision of their paper and addressed all my concerns. I think the author have a novel and quite important core result. I support publication.

(Remarks on code availability)

Open Access This Peer Review File is licensed under a Creative Commons Attribution 4.0 International License, which permits use, sharing, adaptation, distribution and reproduction in any medium or format, as long as you give appropriate credit to the original author(s) and the source, provide a link to the Creative Commons license, and indicate if changes were made.
In cases where reviewers are anonymous, credit should be given to 'Anonymous Referee' and the source.
The images or other third party material in this Peer Review File are included in the article's Creative Commons license, unless indicated otherwise in a credit line to the material. If material is not included in the article's Creative Commons license and your intended use is not permitted by statutory regulation or exceeds the permitted use, you will need to obtain permission directly from the copyright holder.
To view a copy of this license, visit https://creativecommons.org/licenses/by/4.0/

REVIEWER COMMENTS

Reviewer #1 (Remarks to the Author):

This manuscript presents experiments and analyses designed to track burst coding from cortex and thalamus during an ethological learning task. Establishing the role and preponderance of burst coding within the central nervous system has been an important question in system neuroscience, one thought to be a key to understanding learning and attention. The data presented in this paper show novel phenomena that bring an important advance in this question. I thought it was an exciting article and, assuming that the concerns I raise below can be addressed, I would see it well fitted to Nature Communications.
Before stating my concerns, I would like to highlight the pieces I find particularly compelling in the current manuscript. One is the report of fast changes in burst fraction in cells that do not show concomitant changes in tonic firing. Similar fast changes in burst fraction were reported in some areas of thalamus and by Naud et al. eLife (2024) for cortex but for artificial stimuli. I was not aware of such a clear proof of burst multiplexing in cortex as provided by the current manuscript. Another discovery is that of the malleability of these burst responses with reward contingencies. A malleability that is nowhere near that of tonic firing responses (Fig. 3b). Then there is the tracking of such burst response bias throughout learning, showing that burst biases appears slightly before the time where more cells start to encode touches (Fig. 3d vs Fig. 1a), suggesting that whatever this bursting means, it precedes the bulk of the changes in representation. And lastly there are two perturbation experiments that help disambiguate the causal role played by the mechanisms underlying these bursts, although the findings there are less striking. Together, this amounts to a significant advance in our understanding.
My concerns below mainly relate to the final interpretation of these data as presented in the discussion-abstract and to some missing methodological details and their interpretation.

We thank the reviewer for the insightful and encouraging review. We revised the manuscript according to these important inputs in particular pertaining to the reviewer's main concerns related to "the final interpretation of these data as presented in the discussion-abstract and to some missing methodological details and their interpretation." See below for our detailed point-by-point replies to the reviewer comments.

1. Teaching signals and representations. Abstract, intro and discussion mention teaching signals, which were hypothesized to be encoded by bursts in Payeur et al. among others. While this is a great way to justify the importance of the study, I find the evidence, while very interesting, does not support well that the bursting observed are learning signals. At least not in the sense used by Payeur et al. where bursting encoded features of perceptual errors. I would like to note that the evidence presented does not disprove bursting as teaching signals either. While there is unknown degree of perceptual error in the task studied by Heimburg et al., two piece of evidence are at odds with Payeur's perceptual error idea. One is that the burst bias seems to mostly increase during learning. If burst biases encoded an error, this error should start to reduce when the behavior starts to stabilize. The data on this is ambiguous (Fig. 4d). Then it seems to me that the neutral stimuli should elicit more perceptual error as to whether it is

narrow or wide, so more variance in bursting, again ambiguous (Fig 3e). What does fit with Payeur et al. is that these signals occur before the neurons change their selectivities.
Another model that can account for these data is simply a reward contingency. For instance a Q or state value in reinforcement learning would account well for the data in the sense that it should increase with learning the reward association. It seems to me that a reward prediction error (which would be the learning signal in a reinforcement learning context, affecting the Q or state values) does not match well with the data as again, as for perceptual errors, RPE should go down when the task is well mastered and the derivative in d' goes to zero.
Together, I think that if the authors want to make claims about these being learning signals, they should anchor this claim in the right phenomenology. Perhaps it would be safer given the ambiguity of the data to refrain from these claims and only discuss the possible parrallels.

In response to the reviewer's important conceptual point, we have carefully revised the manuscript throughout and now refrain from using the term teaching signal as suggested by the reviewer. Instead, we more carefully frame our findings in the context of reward-contingency learning and emphasize that burst activity tracks the learned behavioral significance of stimuli.

Moreover, we did not intend to interpret BCNs in the context of a canonical RPE encoding signal. For instance, as the reviewer points out, an error signal should faint once the animals make fewer errors. However, we observed a steep ramp up in bursting when the animals started to understand the task and increased their performance (Fig. 4b, d). Thus, rather than an error-prediction, our data show that burst activity dynamically tracks the learned stimulus–outcome relationship: burst bias scales with valence, reverses after rule switches, collapses during degradation, and remains robust after accounting for motor and kinematic variables (see below). We therefore believe that the most appropriate interpretation right now is that BCNs encode learned reward contingency or contextual stimulus value, rather than an error signal.

2. Teaching signals and perturbation. The perturbation of CaV KD and Z944 show a slowing down of learning and a reduction of performance in expert animals. In my understanding, abolishing a teaching signal should affect learning phase and not the performance in expert animals. The data presented would be more consistent with a model where the bursting mechanisms affect the perception, for instance via an attention mechanism. If the bursting signals relate to attention or perception, perturbing them should also slow down learning. The current conclusions of this section seems misplaced to me.

We agree with the reviewer that if bursts were a pure teaching signal, removal of bursts should affect the learning but not the performance in expert animals. We would like to clarify that while expert animals indeed showed a drop in performance after pharmacological block of T-type calcium channels, they maintained a high performance level, well above performance threshold (Fig. 6f), suggesting that the dominant effect of burst removal is not a perceptual deficit but a learning and memory maintenance deficit. To corroborate this, we further analyzed the Z944 effect in expert animals to investigate the time course of the performance drop across the 4 sessions (see figure below). In the initial Z944 session, performance remained similar to expert

performance before Z944 administration, with no significant difference observed relative to the mean performance during the final expert sessions of the preceding stage (Wilcoxon signed-rank test, p = 0.5625). The performance then declined progressively across consecutive sessions to ~1.6 d′ by session 4. This gradual reduction argues against an acute perceptual deficit in task execution and is instead consistent with an active loss of the previously learned rule representation. This is in contrast to manipulations of sensory perception that produce an immediate performance collapse, such as whisker plucking or local lidocaine application, that we observed in our previous study (Heimburg et al., 2025).

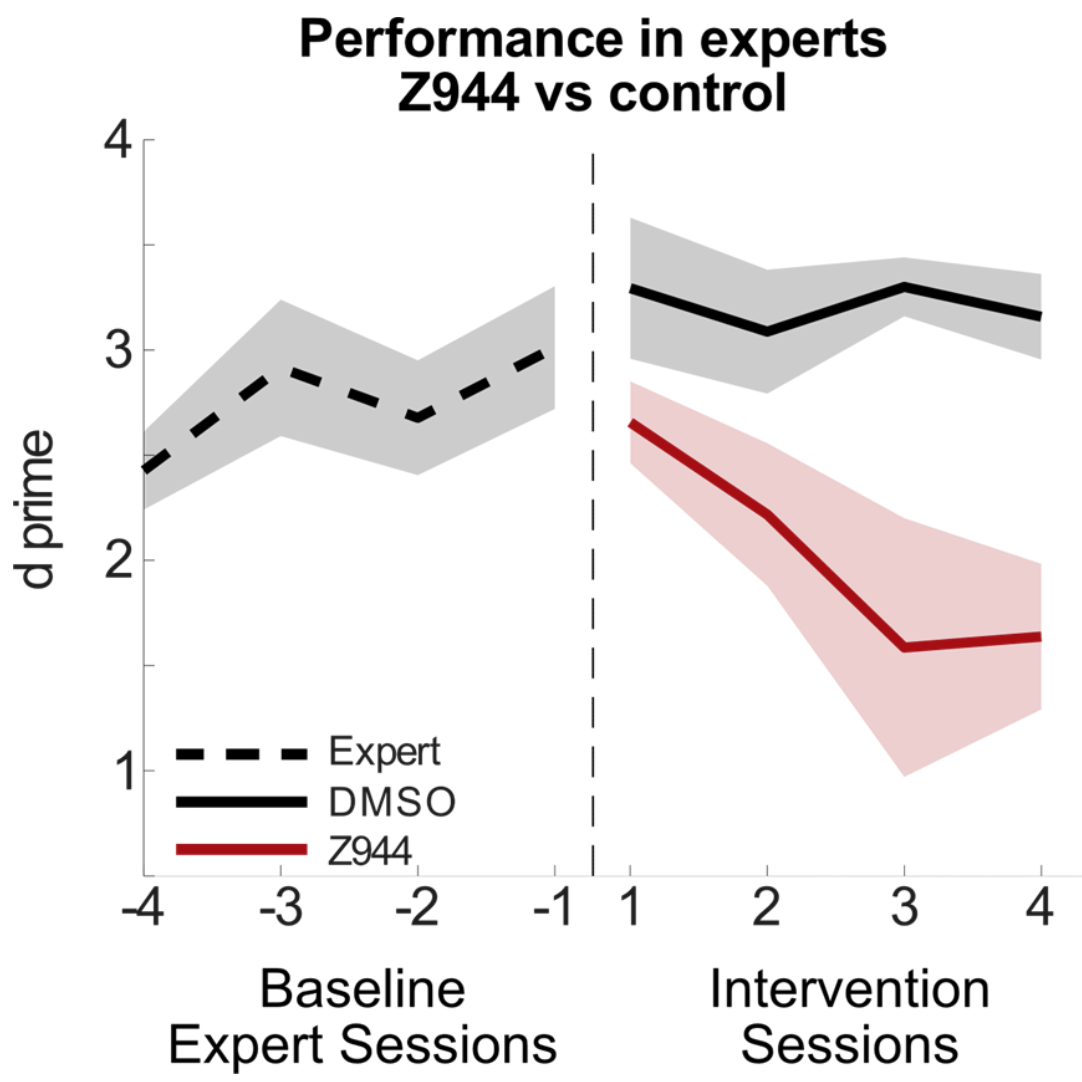

Figure: Task performance before and after Z944 blockage of T-type calcium bursting (n=6 mice)

We have now added this analysis as a replacement for Fig. 6f in the main figure, as we agree that it provides important additional context for interpreting the effects of burst suppression.

Additionally, in order to incorporate the reviewer's concern, we reworked our conclusions to refrain from the term teaching signal and also added a new paragraph at the end of the discussion about alternative interpretations of burst functions in attention and perception.

3. Definition of BCN and TCN neurons. I could not find a precise definition of BCN and TCN. Granted BCN are neurons that show a response in burst fraction, and TCN are neurons that show a response in tonic firing. What are the constraints for the the cross modalities? Are BCN required to have no change in tonic firing and TCN required to have no changes in burst fraction? In which case, the ‘not coding neurons’ contain neurons that respond well in both burst fraction and tonic firing? Otherwise, it could be that the BCN are selected first, such that they contain cells that respond both positively and negatively in terms of tonic firing; then TCN are made of only neurons that do not change in burst fraction and NCN are really non-coding. Regardless, I think the statistical comparisons in Fig. 2c should not be shown since they reflect the definition of BCN and TCN, unless I am missing something.

We acknowledge that the **definitions of BCNs and TCNs** were not sufficiently apparent, even though they were given in the methods. We implemented the reviewer's point by linking the respective results sections to the methods and by adding the terms "definition of BCNs/TCNs" to the subheadings in the methods under the respective "Neural response metrics" sections. We also added a definition of NCNs, which together read now:

"Burst bias, definition of BCNs: The burst bias (BB) is derived from the burst indices of two different aperture conditions (BI1and BI2). It is calculated as the difference between the two burst indices, divided by two. Values can range between -1 (maximal burst bias for aperture 2) and 1 (maximal burst bias for aperture 1). The formula is given by: $BB=BI1-BI22$

A significant burst bias, independent of any concurrent changes in tonic firing, classifies the unit as a burst-coding neuron (BCN), as determined by a Wilcoxon rank sum test.

Tonic bias, definition of TCNs: The tonic bias (TB) is derived from the tonic indices of two different aperture conditions (TI1 and TI2). It is calculated as the difference between the two tonic indices. Values can range between -1 (maximal tonic bias for condition 2) and 1 (maximal tonic bias for condition 1). The formula is given by: $TI = TI1 - TI2$

A significant tonic bias in the absence of burst bias classifies the unit as a tonic-coding neuron (TCN), as determined by a Wilcoxon rank sum test.

Definition of NCNs: Whisker-touch-modulated units that were neither BCNs nor TCNs were defined as non-coding neurons (NCNs), i.e. neither their tonic firing rates nor their burst rates covaried with the aperture width."

Our classification was guided by the primary aim to isolate and characterize the role of burst activity in sensory learning. Accordingly, units were first screened for a significant burst bias between stimulus conditions. Any neuron exhibiting such a burst bias, irrespective of its tonic firing profile, was classified as a BCN. Hence, TCNs exhibit only tonic bias and NCNs neither burst nor tonic bias.

Importantly, across all recorded regions (BC, VPM, POm, and ZIv), the distribution of BCNs remained highly consistent irrespective of the presence or absence of tonic modulation. Replications of the two-dimensional distributions shown in Fig. 3b revealed that the first, second, and third quartile contours (here shown in different shades) changed only marginally when considering tonic bias, indicating that both the central tendency and spread of the BCN population are largely preserved. This suggests that BCNs are primarily defined by their burst bias, whereas concomitant tonic bias has little detectable impact on their overall distribution.

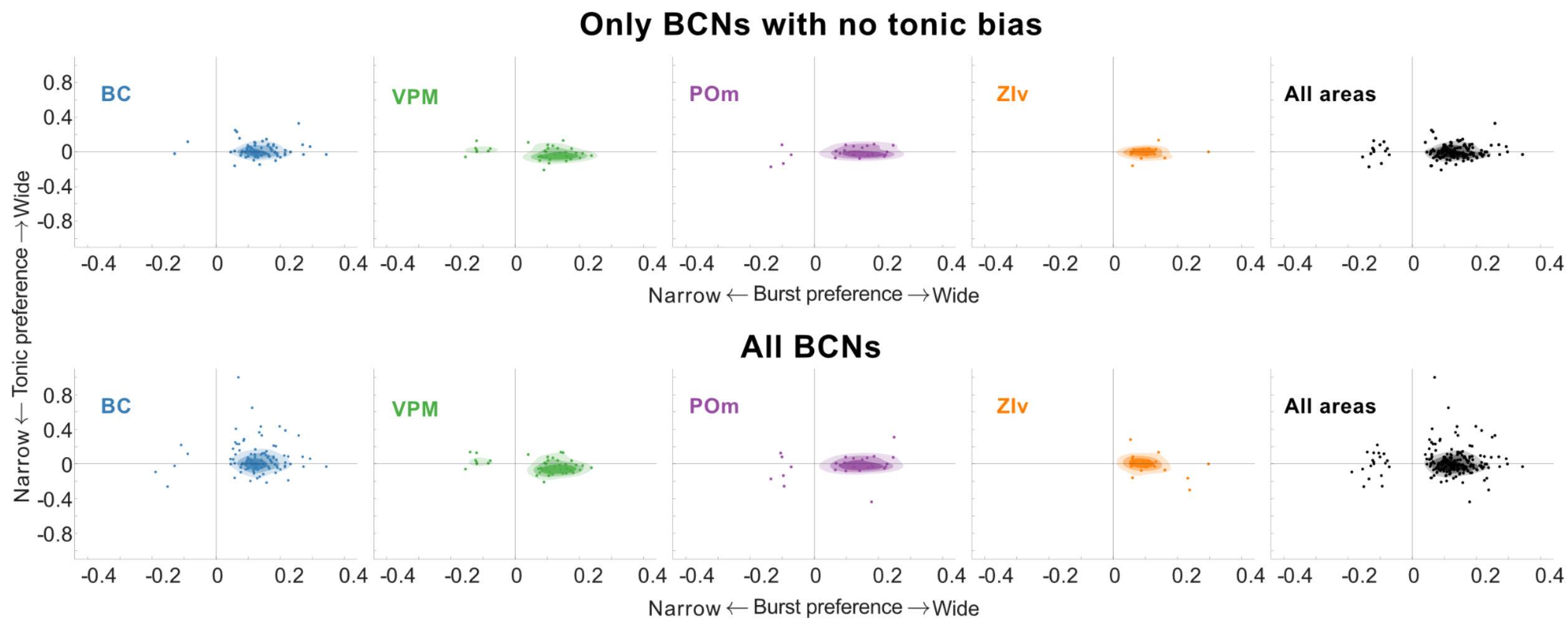

Regarding the **statistical comparisons in Fig. 2c**: these were intended to show how BCNs and TCNs were defined. We apologize that we did not make it clear that these were meant as methodological panels and we fully agree with the reviewer that they appear to present a circular logic. We now explicitly explain that this analysis illustrates our approach to define BCNs and TCNs in the results text as well as in the legend, which reads now:
"Fig.2 c, Spike rasters for example BCN (left) and TCN (right). Burst spikes are marked in red, lick events in blue. BCNs and TCNs were defined by significant differences in their burst or tonic indices in response to the different apertures (shown in adjacent panels for one exemplary BCN and TCN)."

(Note that the fact that BCN have no tonic firing changes implies that these neurons cannot be accounted by a Poisson model, or a model where the firing rate is simply high in some neurons that inherit reward contingencies, I thought this was an important point but up to the authors).

We thank the reviewer for this insightful comment and agree that this has important consequences for future computational models of learning and neural coding, particularly for frameworks relying on conventional stochastic rate assumptions. We have now incorporated this point into the Discussion section, which now reads: *"Importantly, the defining feature of BCNs is not a simple increase in firing rate, but a selective change in temporal structure of spike trains that can occur in the presence or absence of corresponding tonic firing changes. Consequently, BCN activity cannot be adequately captured by conventional Poisson-like firing models that assume stochastic spike generation from a rate variable alone, which may have important implications for future learning models and neural network simulations."*

Most neuroscientist that looks at this type of separation would have the impression that the authors have separated the neurons that fire strongly (BCN) from those that fire weakly (TCN). In order to clarify that this is not the case, one could make a surrogate model where the response spike times are shuffled across cells but within the time bin (function of time to touch). In this way, one would more explicitly separate the burst code from the firing rate code.

We thank the reviewer for this important comment. We agree that, without additional controls, the separation between BCNs and TCNs could be misinterpreted as reflecting differences in overall firing strength rather than distinct coding modes. Given the conceptual importance of this question, we now implemented a surrogate analysis in which response spike times were shuffled, while preserving the overall spike count per unit. This approach effectively disrupts burst spike patterns while maintaining the overall firing rate dynamics. We have included this analysis in Extended Data Fig. 3d, and we have expanded both the Results and Methods sections.

To test whether the number of burst-coding neurons (BCNs) and tonic-coding neurons (TCNs) exceeded expectations based on firing-rate statistics alone, we generated surrogate spike trains for each recording session. For every neuron and trial, the number of spikes occurring within the baseline (−600 to −400 ms) and response (0 to 200 ms) windows relative to the trigger was preserved. Spike times within these windows were then randomly reassigned across neurons by sampling from the pooled distribution of relative spike times across all units in the session, while leaving spikes outside these windows unchanged. This procedure preserved per-neuron spike counts and the overall temporal structure of population-level responses relative to the trigger while removing neuron-specific burst timing structure. For each session, 1000 surrogate datasets were generated, and burst and tonic coding were recomputed using the same analysis pipeline as for the real data.

For each session, Monte-Carlo p-values and z-scores were computed by comparing the observed number of BCNs or TCNs to the surrogate distribution. The p-value corresponded to the proportion of surrogate datasets in which the number of coding neurons was at least as high as in the real data. To assess significance across sessions, the resulting session-wise Monte-Carlo p-values were combined using Fisher's method, yielding a single combined p-value for the dataset. This procedure tests whether the observed deviations from the surrogate expectation across sessions are collectively unlikely under the null hypothesis.

Across sessions, the number of BCNs was significantly greater than expected from the surrogate distributions (Fisher combined Monte-Carlo test: $p = 3.95\times10^{-24}$, $X^2 = 233.21$, df=54), whereas the number of tonic-coding neurons did not differ significantly from the surrogate expectation ($p = 0.070$, $X^2 = 70.02$, df=54), indicating that disrupting the temporal spike pattern selectively affected burst coding but not tonic coding, consistent with the preservation of total spike counts within the baseline and response windows in the surrogate data. Consistent with this interpretation, the distribution of the corresponding z-statistics (computed by comparing the observed per-session counts of BCNs and TCNs to the surrogate null distribution) shows a clear positive shift for BCNs (mean zBCN = 2.91) but remains centered near zero for TCNs (mean zTCN = 0.67) (Extended Data Fig. 3d, and snapshot below).

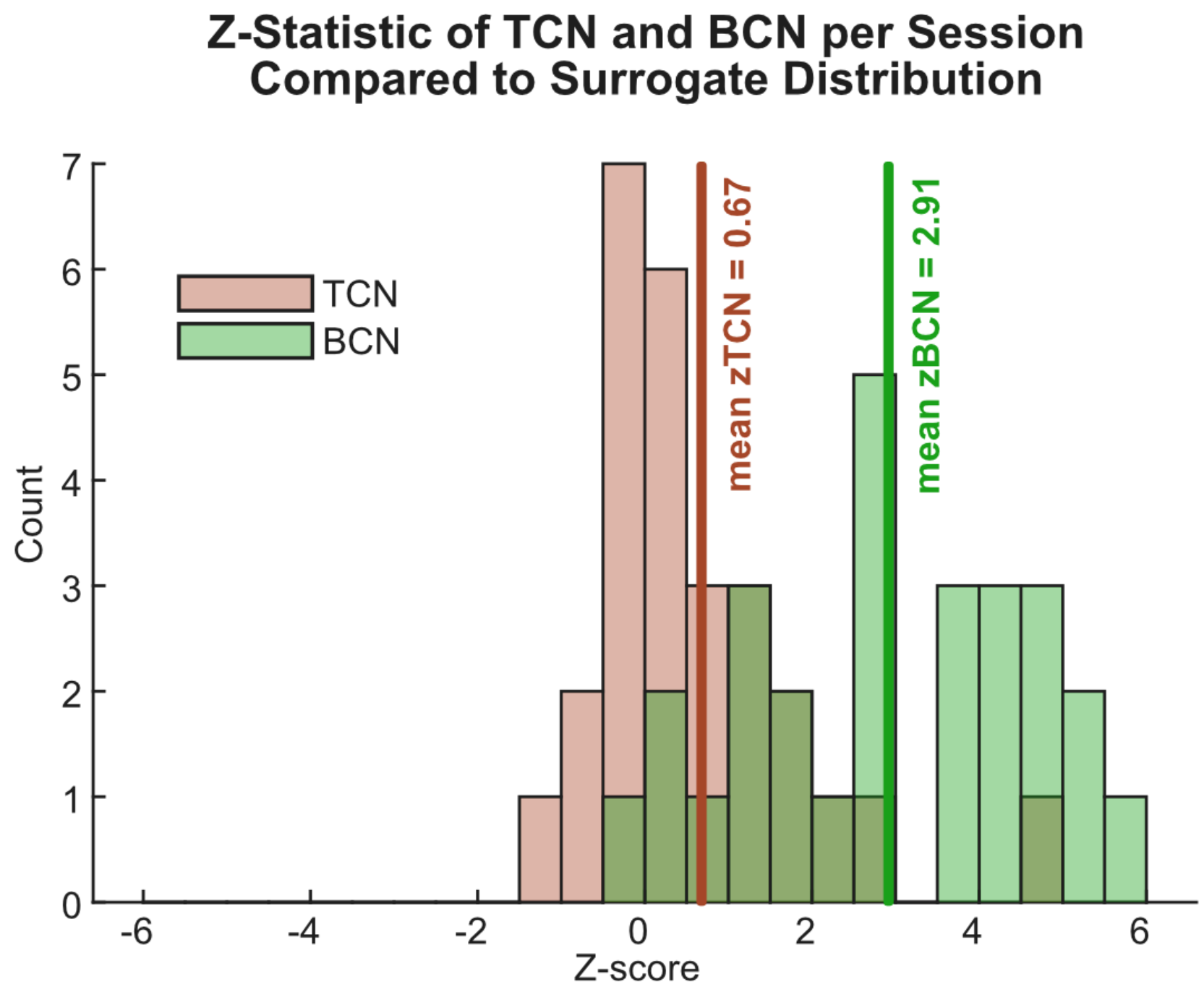

Figure: Histogram of session-wise z-statistics quantifying the deviation of the observed BCN and TCN populations from corresponding surrogate distributions. Positive z-scores indicate stronger-than-expected enrichment relative to surrogate populations. Vertical lines indicate the mean z-score for each population.

Smaller technical points.

- What happens in the calculation of burst fraction when there are no spikes (zero denominator)?

In cases where no spikes are present within a given window, burst fractions are defined as 0. We considered representing such cases as NaN; however, this would exclude trials in which neurons are only bursting in one condition (e.g., exclusively in the response window), thereby biasing the statistical analysis toward consistently and highly active units. By assigning a value of 0, we retain these trials in the analysis while maintaining a conservative estimate of burst contributions.

We added this information in the methods section (under “Burst index”), which now reads:
“P_{bsp} is defined as the number of burst spikes (N_{bsp}) over all spikes (N_{sp}) in a given window. In cases where no spikes are present within a window (N_{sp} = 0), P_{bsp} is defined as 0 to allow inclusion of units that are exclusively bursting in one window.”

- It seems to me that the burst rates reported are very high for sorted units. Fig. 3a reports burst rates of 30 Hz. This seems an order of magnitude too large for cortex. But not too bad for thalamus. So it could be that this number is heavily weighted by thalamus.

The burst firing rates reflect all burst spikes per sec (not bursts as singular events in time). The burst firing rate is thus equal to the firing rate if a neuron responds only with bursts. Since the burst rates shown in Fig. 3a are from BCNs in barrel cortex, which primarily respond with busts (for VPM, POm, ZIv, see Extended Data Fig. 4), these rates are approaching spike rates. These rates are in line with previous studies, for example (O'Connor et al., 2010) showed spike rates of well over 100 Hz in the mouse barrel cortex, especially during presentation of rewarded objects (pole position) and shortly before reward sampling (thus not a reward-related signal, but rather perceptually elicited). Other examples of single-unit firing rates in S1 which are similar to what we report here are Chang et al. (2024) and Minamisawa et al. (2018). We now changed the axes labels in the burst firing plots to "Burst spike rate [Hz]" to prevent confusion. Thank you for pointing this out.

- A error signal should come more in the reverse scenario and with some delay to account for the time to realise there has been an error. I wonder if the authors analyzed this. Fine if not.

We agree with the reviewer that, within classical reinforcement-learning frameworks, one might expect error-related signals to emerge preferentially during reversal conditions and potentially with a delayed temporal profile. While we did not explicitly analyze error prediction signals in this study, we now discuss this possibility in the Discussion as one potentially relevant function of bursting, while emphasizing that the evidence presented here is more directly consistent with bursts encoding learned reward contingencies.

- The claim in the discussion about the linear burst fraction in Payeur et al. seems off to me. I did not fully understand what the authors meant. Payeur et al. needed to linearize the input-output function, which is different from the linear progression.

We thank the reviewer for this important clarification. Our claim relates to the Payeur's et al. (2021) learning model *"(4) Integration of credit-carrying feedback signals should be close to linear and avoid saturation (that is, feedback signals should be linear with respect to any credit information)".* While we completely agree with the reviewer's opinion that the term "linearity" here refers specifically to keeping the feedback-to-burst input–output function in an approximately linear, non-saturated regime, we intended to corroborate this concept on the potential function of bursts in the encoding of credit. In our data, burst activity showed a monotonic valence dependence across three differently valued stimuli, with burst responses decreasing from rewarded to neutral to punished (Fig. 3d-f). This supports the interpretation that burst firing reflects a graded, non-binary signal, that can be modulated through both increases and decreases in activity depending on context.

In order to avoid overstatement, we reformulated the passage, which now reads:
"Furthermore, burst responses were biased toward the rewarded stimulus and were modulated linearly according to valence, which was particularly evident when a neutral stimulus was introduced in our experiments. These observations corroborate the learning model proposed by Payeur et al. (2021) for synaptic plasticity in hierarchical networks, in which bursts were proposed to convey credit-related information when operating in a non-saturated, linear regime."

Reviewer #1 (Remarks on code availability):

I have not looked at the code.

Reviewer #2 (Remarks to the Author):

The manuscript by Heimburg et al. investigates the role of action potential (AP) bursts in thalamocortical circuits during associative learning. Using high-density electrophysiological recordings combined with a whisker-based go/no-go paradigm in freely moving mice (recently developed by the same group), the authors recorded neuronal activity across cortical and thalamic nuclei as animals learned the task. They report that burst firing becomes increasingly prevalent across recorded regions and correlates with behavioral performance over time. Furthermore, burst events appear to encode contextual information about the stimulus, specifically whether it predicts reward. Pharmacological and genetic manipulations that suppress bursting activity in thalamic nuclei disrupted both learning and task performance. Based on these findings, the authors propose that neuronal bursts function as context-sensitive teaching signals that drive associative learning.
Overall, the study addresses an interesting and timely question in the field. The experiments are well executed, and the manipulation experiments are particularly valuable. The analyses are mostly appropriate. However, I find that the central claim is not yet fully supported by the presented evidence. Addressing the concerns detailed below would strengthen the manuscript and enhance the clarity and impact of the study.

Major comments:
1. The central claim of this study is that thalamocortical bursts act as context-sensitive teaching signals that drive associative learning. However, the results presented here more convincingly demonstrate a relationship between thalamocortical bursts and behavioral context (rewarded vs. non-rewarded) or lick execution, rather than learning per se. For example, burst activity shows a strong correlation with the animal's decision to lick (Fig. 3f), and pharmacological suppression of thalamic bursting impaired not only learning but also task performance (Fig. 6f). This raises an important concern: if bursting directly affects sensory perception or behavioral execution, it becomes difficult to disentangle whether bursts specifically contribute to learning processes or instead reflect or modulate performance-related signals. The current experiments do not clearly isolate these possibilities.
Furthermore, the concept of "teaching signal" remains insufficiently defined throughout the manuscript. It is unclear whether the authors propose that bursts instruct downstream circuits to develop task-relevant activity patterns during learning and facilitate updating of representations when task contingencies change. In addition, it is unclear how such teaching signals are expected to evolve with expertise. For example, if bursts serve as teaching signals, should their magnitude decrease once animals reach stable performance, or persist to maintain learned representations? A precise definition of a teaching signal, along with experimental evidence that

distinguishes it from performance-related signals, is necessary to support the central claim of this study.

We thank the reviewer for this important conceptual feedback. We agree that our original wording may have overstated the interpretation of bursts as explicit "teaching signals," and we appreciate the opportunity to clarify this distinction. In response, we have carefully revised the manuscript throughout and now refrain from using the term teaching signal as a central claim. Instead, we frame our findings more conservatively and precisely in terms of reward-contingency encoding during associative learning. In this framework, bursts don't necessarily disappear after expertise is reached. Instead, persistent burst coding in expert animals may indeed reflect the continued maintenance and updating of learned stimulus–outcome associations as the reviewer suggested.

The reviewer brings up the important point of the difficulty to "disentangle whether bursts specifically contribute to learning processes or instead reflect or modulate performance-related signals." Perhaps the most relevant data is seen in Fig. 2c, which shows that touch-evoked bursts appear directly after the touch and before reward-consumption (licking). This delay between sampling and licking, is implemented through the freely-moving task design, which thereby allows to disentangle the sensory sampling phase from the licking phase. The occurrence of bursts before licking argues against a signal that is evoked by reward consumption. Nevertheless, the busts could be viewed as sort of a pure "Go" signal for the animal to move towards the reward spout, without necessarily relating to learning. However, several observations argue against this interpretation. **First,** burst coding develops alongside task learning and reverses after rule switches, indicating that bursting tracks the learned stimulus–outcome relationship rather than fixed sensory features. **Second,** during early learning and degradation phases, licking behavior persists (see also figure below at point #5, displaying behavioral metrics during the degradation stage) while burst bias and BCN proportions remain weak or collapse, demonstrating that licking alone is insufficient to generate the burst code. **Third,** our newly added linear mixed-effects analysis (see below at point #6) shows that aperture condition remains a robust predictor of burst index even after accounting for lick occurrence, lick latency, whisking frequency, whisking cycles, touch number, touch duration, and first-touch latency. Importantly, inclusion of these behavioral covariates did not significantly improve model fit, indicating that burst modulation cannot be explained by concurrent motor or kinematic variables alone. **Fourth,** pharmacological or genetic suppression of bursting during learning impaired learning rate without abolishing licking behavior, as reflected by the persistently negative criterion metric (see figure below), indicative of a liberal response bias (i.e., licking) in signal detection theory. **Finally,** suppressing bursting in expert animals did not induce an immediate collapse of performance but instead produced a gradual decline in task execution (new Fig. 6f). We believe this observation is more consistent with disruption of learned representations than with a primary sensory or motor deficit.

In summary, our observations are more consistent with a framework in which bust-coding emerges with learning and indeed "propose that bursts instruct downstream circuits to develop task-relevant activity patterns during learning and facilitate updating of representations when task contingencies change".

Additionally, we added a new paragraph at the end of the discussion about alternative interpretations of burst functions in attention and perception.

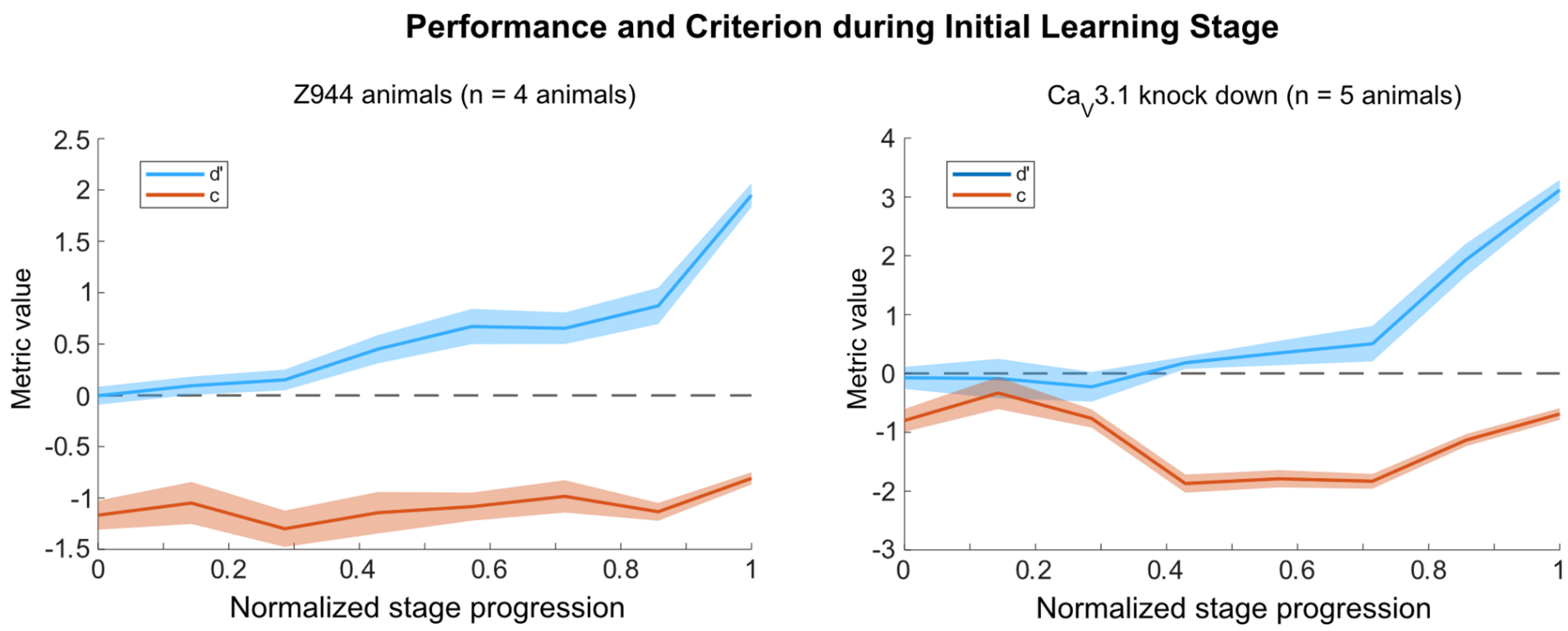

2. Touch-evoked bursting activity increases as animals learn the task and is sensitive to behavioral context (rewarded vs. non-rewarded trials). This modulation is particularly apparent in the barrel cortex, which is frequently highlighted in representative traces and plots, and to a similar or somewhat lesser extent in thalamic regions (Extended Data Figs. 4, 7, 8). The manipulation experiments presented in Fig. 6 provide valuable insight into the role of burst activity in behavior. However, these manipulations are restricted to thalamic bursts and do not address the potential contribution of cortical burst activity. Does this focus reflect a specific mechanistic hypothesis?
Given the prominent modulation of bursting observed in the cortex, assessing the causal contribution of cortical burst activity would substantially strengthen the study and help determine whether bursting plays distinct or complementary roles across thalamic and cortical circuits.

We thank the reviewer for this important point and agree that a direct manipulation of cortical bursting would be highly informative. Our decision to target thalamic bursting first was guided by mechanistic tractability rather than by the assumption that cortical bursts are unimportant. In the thalamus, burst generation is exceptionally well-defined at the cellular level: low-threshold burst firing in relay neurons is tightly linked to T-type Ca^{2+} currents, particularly $Ca_V3.1$-dependent low-threshold spikes (Jahnsen & Llinás, 1984a, 1984b; Kim et al., 2001), and thalamic relay nuclei occupy a central position in both ascending sensory and corticothalamic feedback pathways. This makes thalamic bursting a particularly suitable substrate for causal pharmacological and genetic perturbation. In addition, the thalamus is well established as both a first-order sensory relay and a higher-order node for corticocortical communication, providing a strong mechanistic rationale for testing whether burst modulation there is sufficient to influence learning-related processing across the network.

By contrast, cortical bursting is mechanistically more heterogeneous and less tractable with a comparably specific intervention. Cortical bursting can arise through several interacting processes, including Na^{+}-dependent spikes, dendritic Ca^{2+} spikes, NMDA-dependent

nonlinearities, or backpropagating action potentials (Larkum et al., 1999; Leleo & Segev, 2021). This diversity makes it considerably more difficult to suppress "bursting" in cortex in a selective and interpretable manner without simultaneously altering broader integrative or recurrent cortical computations. There are also more practical constraints in our behavioral paradigm. A spatially restricted cortical manipulation, e.g. within a single barrel, would likely require near-complete whisker plucking except for a single whisker to ensure interpretable sensory specificity. This would introduce a strong sensory deprivation that would alter behavior and cortical function. Conversely, a broader bilateral manipulation of the whole barrel field would risk perturbing large-scale cortical networks in a way that is much less specific than the thalamic interventions used here. For these reasons, we chose thalamic burst mechanisms as the most precise and interpretable entry point for causal experiments in the present study.

We agree, however, that cortical and potentially layer-specific burst mechanisms are an exciting direction that we are currently working on establishing in a new study.

3. Given its formulation, the burst bias (BB) does not distinguish between qualitatively different coding scenarios. For example, BB would be positive both when (1) the burst index (BI) is > 0 for the wide-aperture with BI ~ 0 in the narrow-aperture, and when (2) BI is ~ 0 for the wide-aperture but < 0 for the narrow-aperture. These scenarios reflect distinct coding strategies, yet they yield similar BB values. Distinguishing between burst enhancement and burst suppression would improve the interpretation of how burst activity encodes stimulus or contextual information.
Relatedly, it would be helpful to clarify whether some neurons encode stimulus or contextual information through reductions in burst activity rather than increases.

The reviewer is correct in pointing out that the burst bias, by design, does not distinguish whether a bias arises from enhanced bursting to one stimulus, suppressed bursting to the other, or a combination of both. We found this point important such that we incorporated an additional analysis into the revised manuscript. Specifically, we now quantify the joint distribution of burst indices for wide and narrow apertures and visualize these coding motifs in a two-dimensional representation (Extended Data Fig. 3c, new panels c and d, see also snapshot below). This new analysis shows that BCNs are predominantly associated with enhanced bursting during wide-aperture trials, whereas responses to the narrow aperture are distributed between weak enhancement and suppression.

To characterize how BCNs represent reward contingencies, we quantified burst indices for wide and narrow apertures and plotted their joint distribution. The burst index reflects the change in burst spike proportion between response and baseline windows and ranges from −1 to 1 (see Methods). The two-dimensional distribution revealed distinct coding motifs, with units occupying quadrants corresponding to differential burst modulation across the two stimuli.

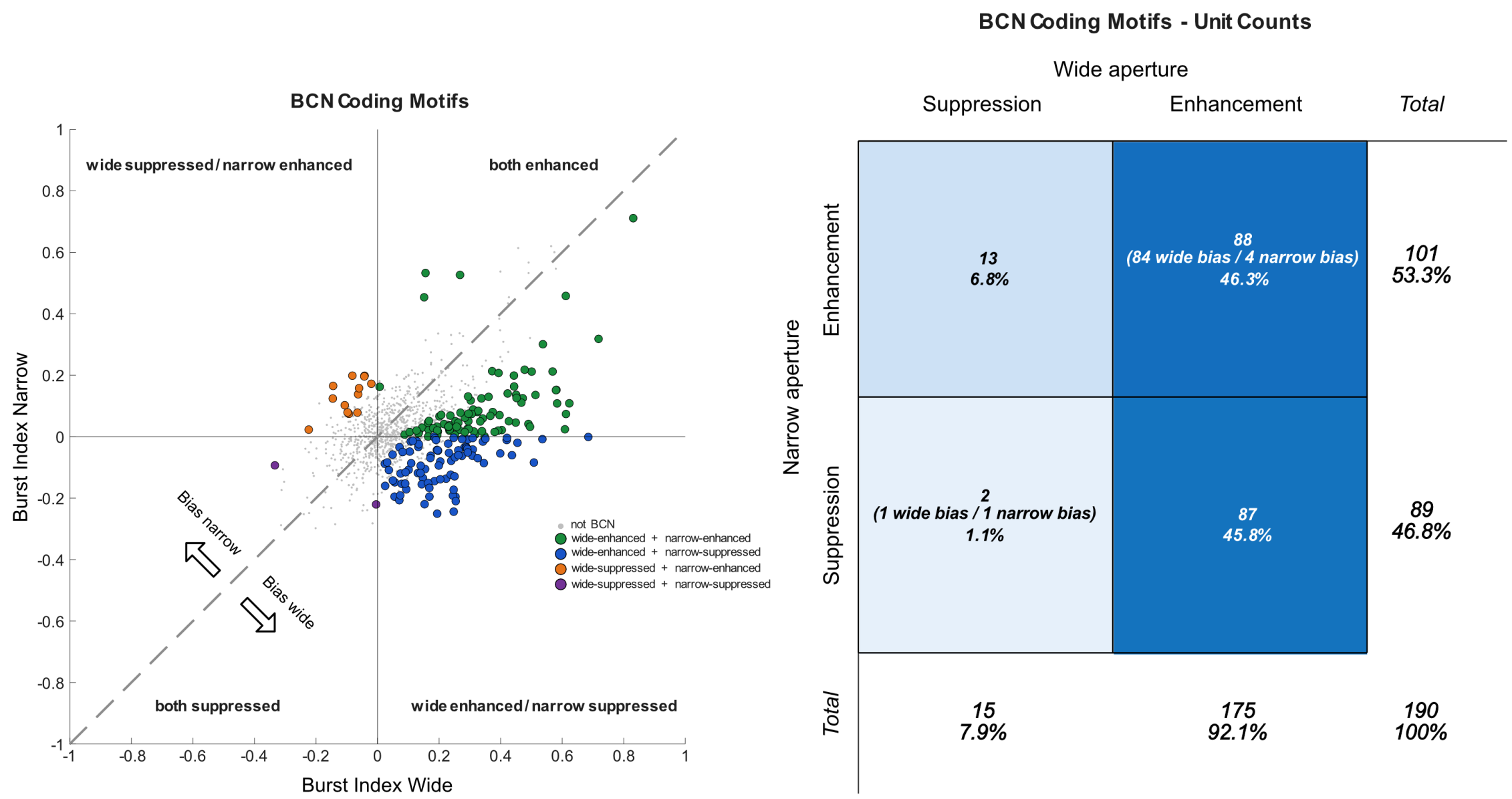

BCNs were predominantly characterized by enhanced bursting during wide-aperture trials, with 92.11% showing increased activity, whereas responses during narrow-aperture trials were more heterogeneous, comprising both weak burst suppression (46.84%) and weak enhancement (53.16%). A smaller fraction exhibited an opposite bias, favoring the narrow aperture, often accompanied by suppressed bursting during wide-aperture trials. Only a minority of units exhibited suppressed responses to both aperture conditions. Together, these findings indicate that the emerging bias toward the rewarded aperture is primarily mediated by increased bursting during its sampling, rather than by suppression of responses to the competing stimulus. Smaller unit clusters may represent specialized neuronal subpopulations that differentially encode stimulus features through variations in burst dynamics.

4. If I understood correctly from the text and statistical summary, the decoding analyses (Fig. 5) appear to have been performed using pooled single-unit data from multiple neurons recorded across six mice. Pooling units across animals does not account for inter-trial covariance among neurons recorded within the same individual animal. For example, neurons recorded simultaneously within the same animal often exhibit trial-to-trial correlated variability due to shared network or behavioral state fluctuations. Such covariance can contribute to population coding. Pooling neurons across animals disrupts these correlations because neurons from different animals cannot share trial-by-trial variability, potentially distorting decoding performance and its interpretation. Decoding analyses performed on neuronal populations within each animal, then followed by comparisons or averaging across animals, would provide a more appropriate and statistically rigorous assessment.

We thank the reviewer for this very important statistical point. We fully agree that pooling units across animals may obscure covariances that naturally exist among simultaneously recorded neurons. To directly address this concern, we performed an additional decoding analysis in

which decoding was computed separately for each animal and subsequently averaged across individuals. Since we considered this issue important, we decided to include the new analysis as new Extended Data Fig. 9 and have expanded the corresponding description in both the Results and Methods sections. Importantly, the animal-wise decoding analysis reproduced the central finding that BCNs outperform other touch-modulated populations in stimulus decoding.

At the same time, splitting decoding analyses by individuals substantially reduced the number of simultaneously available units per recording site, limiting the achievable population sizes for meaningful decoding curves for individual recording sites. We therefore pooled units from all four recording sites in this new analysis. In addition, covariance structures may also be expected within individual brain regions themselves, making the original area-wise analysis still informative from a population-coding perspective. We therefore retained the original analysis in the main figure (Fig. 5) while adding the new animal-wise decoding analysis to explicitly address concerns regarding inter-animal covariance and generalizability.

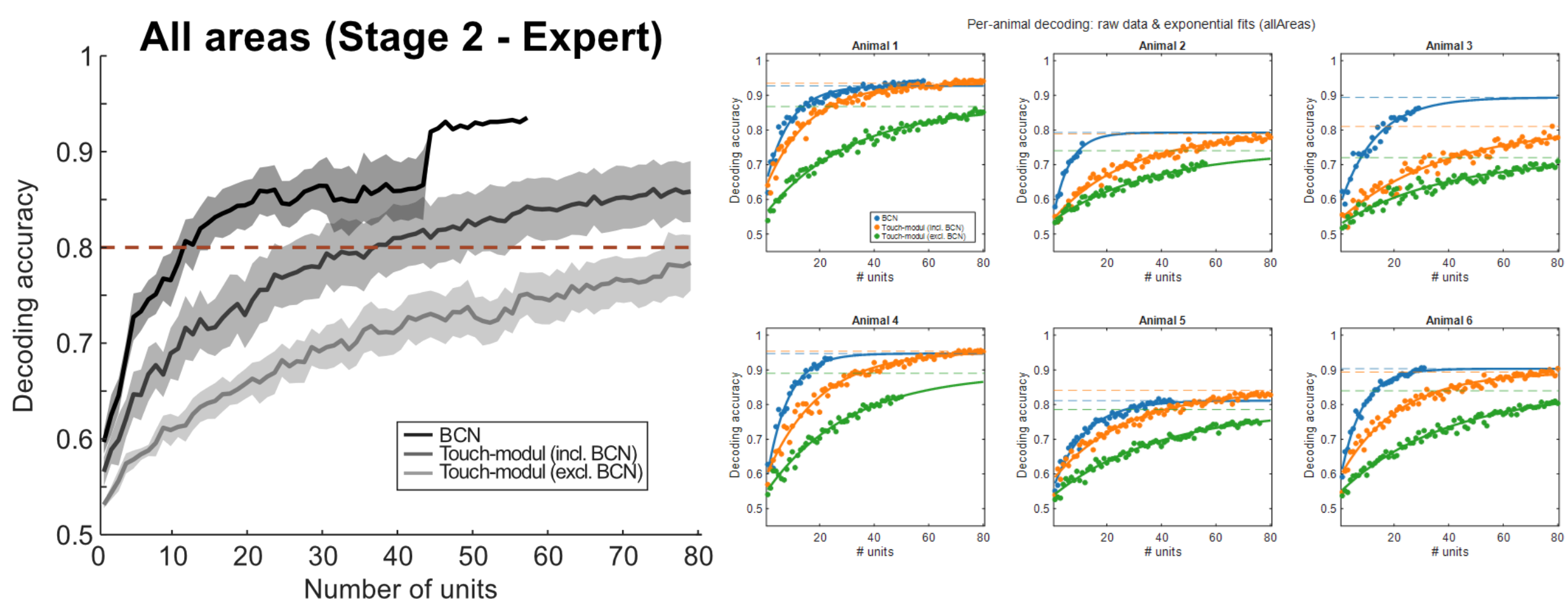

Figure: Decoding accuracy as a function of population size, decoded per animal and then averaged across animals. The plot shows decoding accuracy for BCN units, all touch-modulated units (including BCNs), and touch-modulated units excluding BCNs, pooled across all recorded areas. BCN populations consistently achieved the highest performance, showing that BCNs encode more information than touch-modulated cells per se. The lower performance observed after excluding BCNs from the touch-modulated pool indicates that BCNs account for a substantial fraction of the decodable signal.

To achieve a decoding accuracy of 80% or higher, the number of units required was significantly lower for the BCN group (12 units) compared to the touch-modulated group including BCNs (37 units) and excluding BCNs (more than 80 units).

For each animal and condition, decoding accuracy as a function of unit count was modeled using a negative exponential saturation function,

$$Acc(n) = A - (A - A_0)e^{-kn},$$

where A_0 denotes the baseline accuracy at a hypothetical unit count of zero, A the asymptotic decoding accuracy, and k the saturation rate constant. Paired comparisons of the fitted parameters across conditions (two-sided paired t-tests) revealed that BCNs exhibited a significantly higher saturation rate than touch-modulated populations, both when BCNs were included in the touch-modulated pool (p=0.0022) and when they were excluded (p=0.001). In addition, the asymptotic accuracy was significantly higher for BCNs than for touch-modulated cells excluding BCNs (p=0.0191), whereas it did not differ from that of the full touch-modulated population including BCNs (p=0.6033). No significant differences were observed in baseline accuracy between any of the compared conditions (p>0.05 for all comparisons).

5. The authors claim that “un-learning” diminishes burst biases (Line 366). However, based on the presented plots in Fig. 3c, it appears that burst biases segregate into two subpopulations, one with BB > 0 and the other with BB < 0, such that the population average approaches zero, rather than individual neurons converging toward zero bias. If this interpretation is correct, the conclusion that un-learning reduces burst bias at the single-neuron level may need to be reconsidered or clarified.

Thank you for this important point. We further stratified our analysis in Fig. 3c to address this point. We find that the diminished burst bias during un-learning is the result of both a population effect (the relative proportion of narrow- and wide-encoding subpopulations) and a reduced burst bias on the single unit level. The upper panel shows how the bias distributions in BC, VPM, POm and ZIv shift across task phases, indicating dynamic changes in population-level aperture tuning as well as on the unit level. In the degradation phase, the asymmetry is reduced, with no strong bias toward either aperture.

At the same time, the single-unit burst bias magnitude (as the absolute burst-bias values) of pooled BCNs was significantly lower during degradation than during the expert initial rule phase (Wilcoxon rank-sum, $p = 8.47 \times 10^{-7}$) or compared to all expert sessions across the initial, reversed and restored rules (Wilcoxon rank-sum, $p = 0.0188$). These findings indicate that the reduction in burst bias during degradation cannot be explained solely by a redistribution of narrow- and wide-preferring neurons, but also reflects a genuine attenuation of aperture-selective burst coding among the sampled single units.

The lower panel directly reflects the population effect: the relative fractions of narrow- and wide-encoding BCNs track the task rule and become more balanced during degradation.

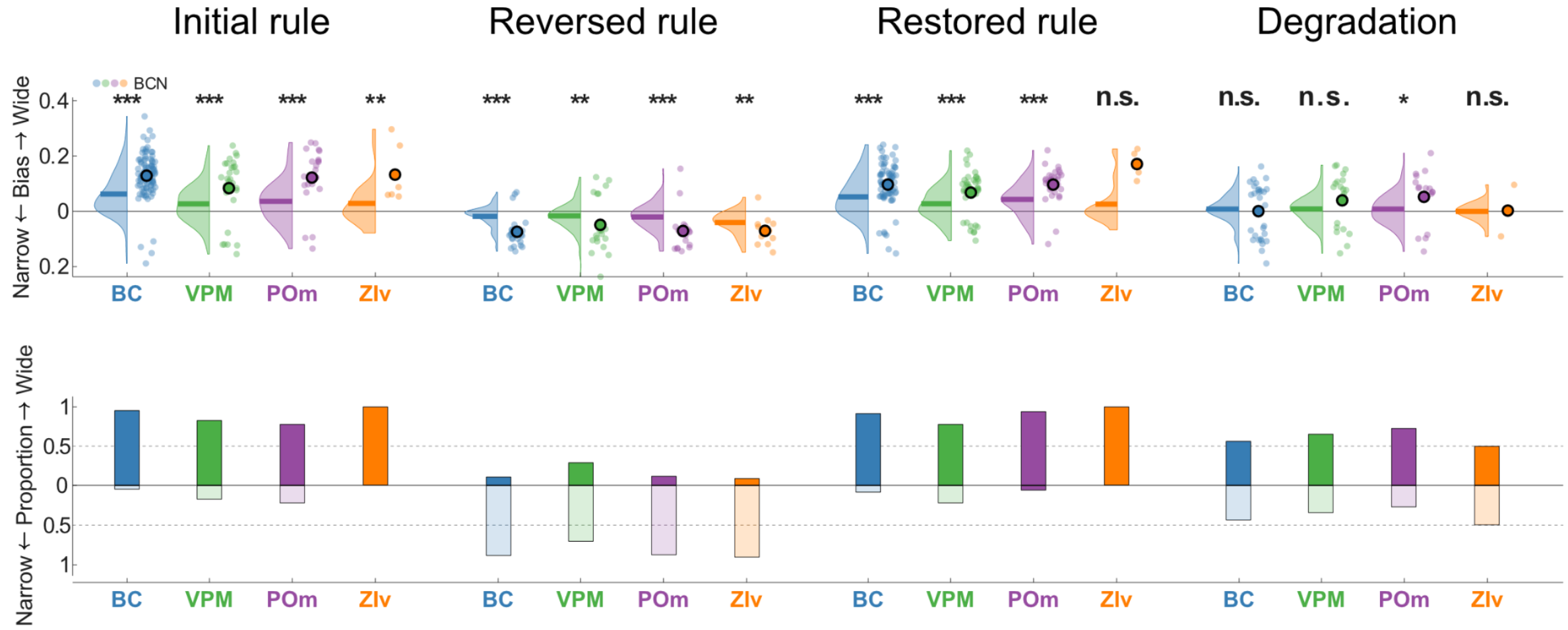

Furthermore, the analysis now also allows comparison of the overall population of touch-modulated units to BCNs only. Here we observe that the mean aperture bias of BCNs is distinct from that of the overall population, indicating that these units do not merely represent the tails of a continuous distribution of aperture tuning and their encoding properties are not captured by population-wide averages.

We would also like to note that in the data set for this study, single units could not be tracked across sessions. Therefore, conclusions about the longitudinal behavior of the same single units across different learning phases are currently not possible. We are currently establishing recording methods to track single units over time, so that we will hopefully be able to answer this important question with more rigour in a future study.

Relatedly, it would be helpful to examine behavioral performance metrics during the un-learning phase. Although Fig. 1d shows that d' approaches zero during "contingency degradation", it remains unclear whether this reflects balanced hit and false alarm rates or a general disengagement from the task. Distinguishing between these possibilities may help interpretation of the persistence of neuronal subpopulations with BB > 0 and BB < 0 during un-learning.

During the degradation stage (n = 6 animals), sensitivity (d') remained near zero, while decision criterion (c) was strongly negative across sessions. Within signal detection theory, criterion reflects response bias independently of sensory discriminability, with negative values indicating a liberal response strategy and positive values indicating a conservative one. The persistently negative criterion therefore indicates a pronounced lick bias, i.e., a strong tendency to lick regardless of trial type. This was reflected by the behavioral outcome profile, with hit rates remaining near the ceiling and correct rejection rates close to zero, consistent with poor stimulus discrimination. Notably, trial counts remained high overall (median [IQR] = 112.5 [80.5, 168.5]; mean ± SEM = 129.33 ± 9.25), demonstrating preserved task engagement, comparable to the final four sessions of the preceding rule-switch phase (median [IQR] = 114 [95, 128.5];

mean ± SEM = 118 ± 13.19). This indicates that the observed performance decline reflects a change in response strategy rather than reduced motivation or disengagement.

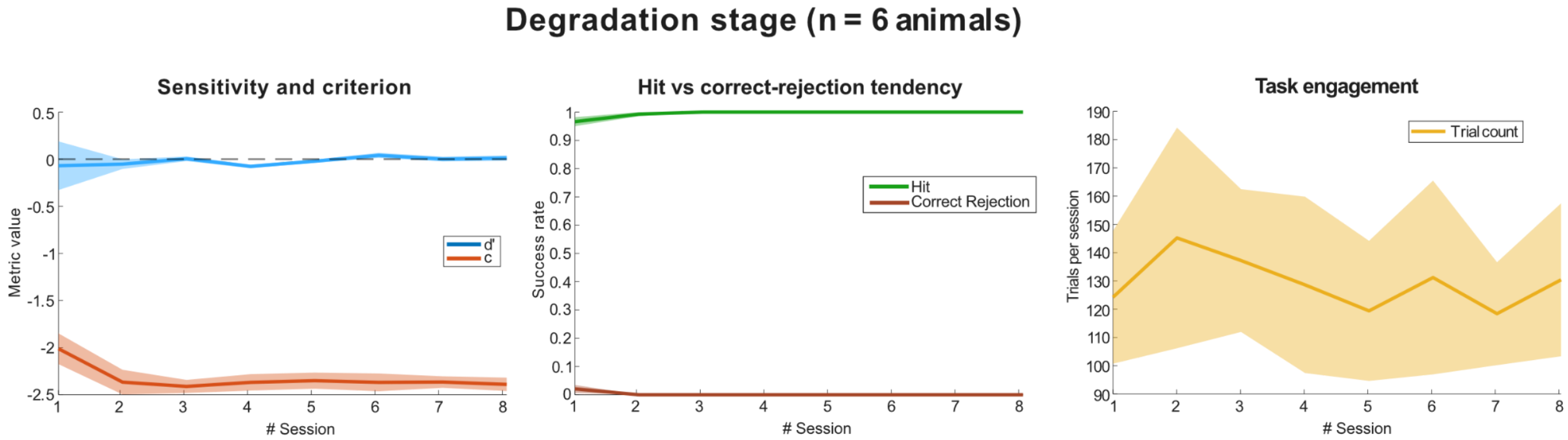

Figure: Behavioral metrics during the degradation stage (n = 6 animals). Left, sensitivity (d') and criterion (c). Middle, hit rates and correct rejection rates. Right, trial counts. Curves show mean ± SEM.

6. The manuscript does not provide measurements of whisker behavior (e.g., number of whisker-aperture contacts, whisking amplitude, etc). These variables are important to support the claim that burst activity encodes contextual information, rather than simply reflecting differences in sensory input or motor output between go and no-go stimuli. Without quantifying whisker contacts and movement parameters, it remains difficult to exclude the possibility that differences in burst activity arise from variations in tactile sampling or whisking strategies across conditions.

We appreciate this constructive comment. To assess the dependency of aperture-related modulation of bursting in BCNs on within-trial whisker kinematics and licking behavior, we fitted population-level linear mixed-effects models predicting per-trial burst index from aperture condition (fixed effect). Random intercepts were included for unit identity, animal, and brain region to account for hierarchical data structure. Analyses were restricted to BCNs during expert performance (n = 197 of 1,241 units) to specifically test whether burst activity is modulated by aperture state independently of behavioral covariates.

Wide-aperture trials were associated with significantly higher burst indices compared to narrow-aperture trials (estimated effect: Δburst index = +0.21, 95% CI [0.13, 0.29], Cohen's d = 0.53). Importantly, this effect remained robust after adjusting the model for lick occurrence, lick latency, whisking frequency, whisking cycles, number of touch events, median touch duration, and first-contact latency (estimated effect: Δburst index = +0.20, 95% CI [0.11, 0.28], Cohen's d = 0.49). The modest reduction in effect size by only 7.8% suggests that aperture-related burst modulation in BCNs is largely robust to behavioral and kinematic variables. Consistent with this, adding these covariates did not significantly improve model fit relative to an aperture-only model (likelihood ratio test: Λ = 10.4, df = 7, p = 0.166), indicating that they do not explain additional variance in burst index beyond aperture condition.

In summary, BCNs burst more strongly in wide-aperture trials than in narrow-aperture trials, and this effect persisted after controlling for trial-by-trial differences in licking, whisking, and touch.

This indicates that bursting is robustly associated with aperture width, rather than just a consequence of different behavioral patterns across trials.

Methods: Linear mixed-effects models (LME) analysis of aperture-driven burst modulation

To test whether aperture-dependent differences in burst index reflect a stimulus-driven modulation of bursting that is independent of within-trial whisker kinematics and lick behavior, we fit a series of population-level LMEs to per-trial burst indices from burst coding neurons (n = 197 units). The response variable was the per-trial burst index (see below for definition), which ranges continuously from −1 to +1, with positive values indicating stimulus-evoked burst enhancement. Restricting the analysis to BCNs introduces a degree of circularity. However, the aim was not to test for significance, but to assess how robust the effect remains after adjusting for behavioral covariates.

For each BCN, the trial-wise burst index vector was merged with seven per-trial behavioral covariates (lick occurrence, lick latency, whisking frequency, whisking cycles, number of touch events, median touch duration, and first-contact latency), which were extracted from high-speed video data processed using DeepLabCut (see below). Three nested LMEs were fit using maximum likelihood (fitlme, MATLAB) with random intercepts for animal, unit identity, and brain region:

Aperture-only model:

$$BurstIndex \sim Aperture + (1 \mid Animal) + (1 \mid UnitKey) + (1 \mid BrainRegion)$$

Full model:

$$\begin{aligned} BurstIndex \sim\ & Aperture + Lick + LickLatency + MeanWhiskingFq \\ & + WhiskCycles + TouchEvents + TouchDuration + FirstTouchLatency \\ & + (1 \mid Animal) + (1 \mid UnitKey) + (1 \mid BrainRegion) \end{aligned}$$

Covariates-only model:

$$\begin{aligned} BurstIndex \sim\ & Lick + LickLatency + MeanWhiskingFq \\ & + WhiskCycles + TouchEvents + TouchDuration + FirstTouchLatency \\ & + (1 \mid Animal) + (1 \mid UnitKey) + (1 \mid BrainRegion) \end{aligned}$$

The behavioral attenuation fraction was computed as: $attenuation = 1 - \frac{EffectSize_{Full}}{EffectSize_{Aperture-only}}$

A likelihood-ratio test was performed, comparing the full model against an aperture-only model. The test statistic $\Lambda = 2 \times (\ell_{Full} - \ell_{Reduced})$ was tested against a χ^2 distribution with degrees of freedom equal to the difference in the number of fixed-effect coefficients (df = 7). In other words, this test evaluated whether adding behavioral covariates provides a significant improvement in explaining variance in burst response beyond the effect of aperture alone.

Minor comments:

1. Line 42 "… and has so far mainly been attributed to hippocampal circuits18.19." Based on the cited references, I believe that the authors are referring to hippocampal sharp-wave ripples. However, sharp-wave ripples are typically characterized as population-level burst events, reflecting synchronized activity across neuronal ensembles, which differs from single-cell burst firing, the primary focus of the present study. Clarifying this distinction would help avoid conceptual confusion and would better position the current study relative to prior literature.

Thank you for pointing this out. We rewrote this part as suggested and moved it to the discussion which reads now: *"While memory functions have been linked to hippocampal population burst activity, known as sharp wave ripples (Buzsáki, 2015; Joo & Frank, 2018), our work reveals that the thalamocortical system carries its own previously unknown single neuron burst code that directly drives learning."*

2. Fig. 2a: The definition and calculation of "Learning progression" on the x-axis should be provided.

We appreciate the reviewer's comment regarding this point of clarification. The x-axis refers to the normalized progression within the initial training stage, where 0 corresponds to the beginning and 1 to the end of the stage. To make this more explicit, we have revised the axis label in Fig. 2a, as well as Fig. 4b,d, Fig. S7 and Fig. S8, and to "Normalized stage progression".

3. Fig. 4d, middle panel: the y-axis is labeled "wide <-> narrow," while in Extended Data Fig. 8 the y-axis is labeled "narrow <-> wide." This inconsistency is confusing.

We thank the reviewer for pointing out this inconsistency. The original inversion of the axis orientation was intended to facilitate tracking changes in burst bias in relation to the gradual increase in behavioral performance. We have now adopted a consistent axis convention across Fig. 4d and Extended Data Fig. 8 to improve clarity.

4. Extended Data Fig. 5, right panels: The title of the y-axis should be "tonic preference".

Thank you for this careful observation and apologies for the oversight in the axis labeling. To ensure consistency with Fig. 3b, we have revised the labeling and now use the same convention of "Tonic bias" and "Burst bias" for the respective axes.

5. Fig. 4c,e and Extended Data Fig. 7 and 8, right panels: The statistical summary table states "Correlation of performance … in expert sessions." If this is the case, it is unclear why the plots include data points with d' values close to zero.

We thank the reviewer for this careful observation. The description in the statistical summary table was misleading, as the plots in Fig. 4c,e and Extended Data Fig. 7 and 8 include all sessions within each stage (initial rule and reversed rule), rather than only expert sessions, as the plots are intended to capture the full learning trajectory. We have revised the labeling in the statistical summary table to accurately reflect this.

Reviewer #3 (Remarks to the Author):

IIn their paper ‘Thalamocortical bursts encode reward contingencies and drive associative learning‘ Heimburg et al describe single-cell activity and burst-firing patterns in the rodent somatosensory corticothalamic system during an aperture discrimination task. The authors observe a strong correlation of burst-firing and reward-contigencies, i.e., neurons burst in response to the rewarded aperture. The authors show with rule reversal experiments that these responses are indeed contingent on reward and not specific sensory cues. Finally, the authors use an iRNA approach to interfere with bursting and find effects on learning. I think this is a paper of considerable significance, because the core finding is novel and potentially very important.

Major:

1. Reward-contingent burst-firing

The observation of reward-contingent burst-firing is extraordinary. The reasons for this assessment are the following: (i) We have had many hypotheses about burst firing in the past (thalamic tonic firing vs bursting in sleep vs wakefulness, packaging of information etc etc), but this observation is different. (ii) The evidence presented by the authors is very clear. (iii) What adds to the significance of the finding is that the reward-contingent burst-firing seems to work across structures (thalamus and cortex). This is very unexpected, because investigators generally proposed very different explanations for bursting in thalamus (low-threshold Ca-spikes) and cortex (here cell-type specific explanations predominate, i.e., intrinsic bursting of layer 5 pyramids, dendritic burst mechanisms and the like. This is not, what the authors observe, however. (iv) Finally, reward coding is immensely important in the brain.

Thank you for this positive feedback and for sharing the excitement over these findings.

2. Last sentence of the abstract

In the last sentence of the abstract the authors link their burst coding results to memory formation. My impression is that this link stretches the results somewhat. Sticking to reward contingency is the more proximal and better substantiated interpretation here.

Thank you for pointing this out. We changed this sentence as suggested, which now reads: ”These findings identify a previously unknown burst-based neural code for stimulus–outcome

associations in the thalamocortical system and provide causal evidence linking cellular firing dynamics to reward contingency learning."

Minor:

1. It would be great to see some additional information about the cortical neurons recorded here (layers etc).

We are currently conducting a follow-up project in which mice perform the same whisker-dependent Go/No-Go task while neural activity is recorded from the barrel cortex using Neuropixels probes. The higher spatial resolution of these probes allows us to assign recorded units to specific barrel cortex layers using post hoc histological reconstruction of probe tracks and alignment to the Allen Mouse Brain Atlas. As a preliminary analysis, we examined the distribution of BCNs across barrel cortex layers and separated sessions according to behavioral performance. This initial analysis suggests that BCNs are present across layers, with an apparent enhancement in the deeper layers during high-performance sessions.

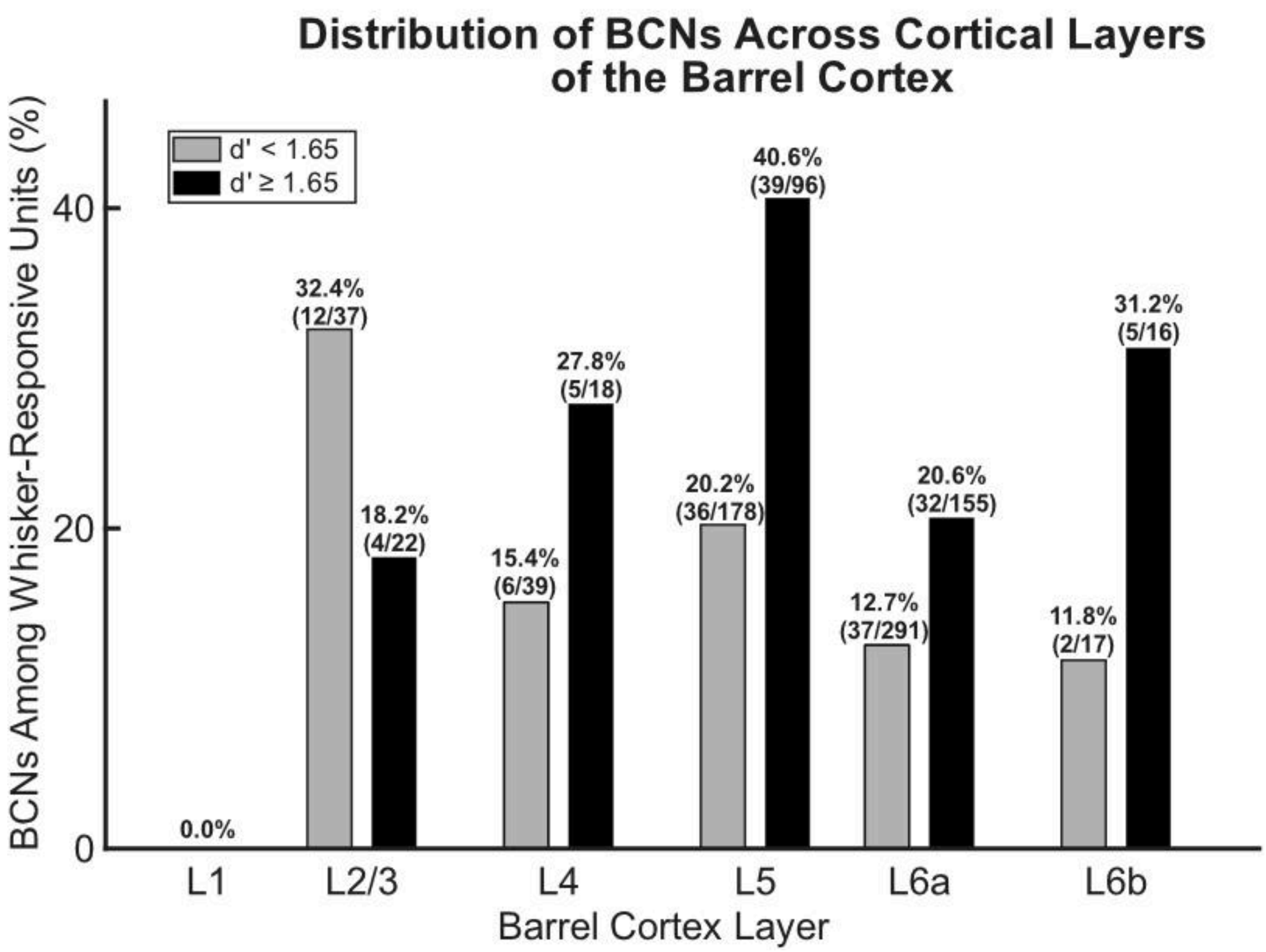

Figure: Layer-wise distribution of burst-coding neurons (BCNs) among whisker-responsive units in Barrel Cortex. Bars show the percentage of whisker-responsive units classified as BCNs in each cortical layer, separated by session performance level: low-performance sessions (d′ < 1.65, gray) and high-performance sessions (d′ ≥ 1.65, black). Percentages are indicated above

each bar; values in parentheses indicate the number of BCNs over the total number of whisker-responsive units in that layer and performance group. Data are from n=2 mice.

3. The authors target a T-type Ca-channel to affect bursting. Is this a plausible candidate for reward-contingent burst-firing across the somatosensory system? Please comment.

We thank the reviewer for this important question. Our rationale for targeting T-type Ca^{2+} channels was based on the view that they constitute a particularly well-defined mechanistic substrate for burst generation at the thalamic level. Low-threshold burst firing in thalamic relay neurons is tightly linked to T-type Ca^{2+} conductances, especially $Ca_V3.1$-mediated low-threshold spikes (Jahnsen & Llinás, 1984a, 1984b; Kim et al., 2001), and thalamic relay nuclei occupy a central position in both ascending sensory and corticothalamic feedback pathways. Thus, these channels provide a biophysically plausible substrate through which behavioral context and learned contingencies could shape thalamic burst probability. This makes thalamic bursting a particularly suitable substrate for causal pharmacological and genetic perturbation.

At the same time, we do not interpret our data as evidence that T-type Ca^{2+} channels themselves encode reward information directly. Rather, our findings suggest that these channels constitute a mechanistically accessible cellular substrate for burst signaling that can be modulated pharmacologically and genetically to shape reward-related network dynamics.

By contrast, cortical bursting is mechanistically more heterogeneous and less tractable with a comparably specific intervention. Cortical bursting can arise through several interacting processes, including Na^+-dependent spikes, dendritic Ca^{2+} spikes, NMDA-dependent nonlinearities, or backpropagating action potentials (Larkum et al., 1999; Leleo & Segev, 2021). This diversity makes it considerably more difficult to suppress bursting in cortex in a selective and interpretable manner without simultaneously altering broader integrative or recurrent cortical computations. For these reasons, we considered thalamic T-type channel–dependent bursting the most precise and interpretable entry point for testing the causal contribution of burst firing to learning-related processing in the present study.

4. How could reward and bursting be linked cellularly? What are the reward related signals that get into corticothalamic system? Is there an effect of dopamine application on thalamocortical bursting.

We thank the reviewer for this mechanistically important question. We agree that the cellular route by which reward-related information becomes linked to thalamocortical bursting is a central issue raised by our findings, and one that our present data do not yet fully resolve.

An interesting observation in this context is that POm bursting emerges very rapidly after whisker contact, in fact earlier than the classic phasic dopaminergic response latencies reported by Schultz and colleagues as approximately 50–110 ms (Schultz, 1998) and in a more recent study in mice as approximately 45 ms for reward-predictive cues (Pan et al. 2021). This timing makes a direct, first-pass dopaminergic explanation of the sensory-evoked POm burst response

less likely and instead suggests that the thalamic signal may lie functionally upstream of, or parallel to, canonical dopaminergic reward responses.

Mechanistically, we think several routes are plausible. A first possibility is that such signals are integrated within higher-order thalamic circuitry itself, and then propagated through thalamocortical networks. POm is deeply embedded in recurrent corticothalamic loops and is widely regarded as a convergence node for ascending sensory input, corticothalamic feedback, and behavioral-state-related modulation, placing it in an ideal position to express learned stimulus–outcome contingencies rather than merely relaying sensory information (Audette et al., 2019; La Terra et al., 2022). Recent work has shown that POm provides a behaviorally relevant output to dorsal striatum (Yonk et al., 2025) and inhibition thereof slows reaction times and learning acquisition. These findings support the idea that POm is positioned to integrate sensory, state, and contingency-related signals to distribute them to both cortical and subcortical circuits involved in adaptive behavior.

A second, not mutually exclusive, possibility is that dopaminergic or other neuromodulatory systems influence this process more indirectly. The thalamus receives dopaminergic innervation, and dopamine has been proposed to modulate thalamic responsiveness and excitability (Varela, 2014), potentially gating plasticity locally while also recruiting higher-order cortical regions that feed back onto thalamocortical hubs. At present, however, we are not aware of definitive evidence showing that dopamine application specifically controls burst mode in POm or VPM in the context of reward learning.

Although our data do not provide sufficient evidence to support a dopamine-dependent burst mechanism, this remains an interesting avenue for future investigation.

5. The authors might want to consider the apical tuft data reported by Benezra from the Bruno lab.

We thank the reviewer for this suggestion. We agree that recent findings from the Bruno lab on apical tuft activity provide a potential link between our results and mechanisms by which learning reshapes task-relevant representations in other parts of the somatosensory system. We have therefore incorporated this study into the revised Discussion, which now reads: *“[...] our work reveals that the thalamocortical system carries its own previously unknown single neuron burst code that directly drives learning. This possibility is particularly compelling in light of recent findings demonstrating that reinforcement learning induces persistent realignment of apical tuft responses in layer 5 barrel cortex neurons (Benezra et al. 2024), raising the possibility that thalamocortical burst signals may interact with dendritic integration and plasticity mechanisms in the cortex.”*

Bibliography

Audette, N. J., Bernhard, S. M., Ray, A., Stewart, L. T., & Barth, A. L. (2019). Rapid plasticity of higher-order thalamocortical inputs during sensory learning. *Neuron*, *103*(2), 277–291.e4. https://doi.org/10.1016/j.neuron.2019.04.037

Buzsáki, G. (2015). Hippocampal sharp wave-ripple: A cognitive biomarker for episodic memory and planning: HIPPOCAMPAL SHARP WAVE-RIPPLE. *Hippocampus*, *25*(10), 1073–1188. https://doi.org/10.1002/hipo.22488

Chang, Y.-T., Finkel, E. A., Xu, D., & O'Connor, D. H. (2024). Rule-based modulation of a sensorimotor transformation across cortical areas. *eLife*, *12*. https://doi.org/10.7554/eLife.92620

Heimburg, F., Saluti, N. M., Timm, J., Adlakha, A., Bortolozzo-Gleich, M. H., Martín-Cortecero, J., Castelanelli, M., Klumpp, M., Embray, L., Both, M., Kuner, T., & Groh, A. (2025). A tactile discrimination task to study neuronal dynamics in freely-moving mice. *Nature Communications*, *16*(1), 1–20. https://doi.org/10.1038/s41467-025-61792-0

Jahnsen, H., & Llinás, R. (1984a). Electrophysiological properties of guinea-pig thalamic neurones: an in vitro study. *The Journal of Physiology*, *349*(1), 205–226. https://doi.org/10.1113/jphysiol.1984.sp015153

Jahnsen, H., & Llinás, R. (1984b). Ionic basis for the electro-responsiveness and oscillatory properties of guinea-pig thalamic neurones in vitro. *The Journal of Physiology*, *349*(1), 227–247. https://doi.org/10.1113/jphysiol.1984.sp015154

Joo, H. R., & Frank, L. M. (2018). The hippocampal sharp wave-ripple in memory retrieval for immediate use and consolidation. *Nature Reviews. Neuroscience*, *19*(12), 744–757. https://doi.org/10.1038/s41583-018-0077-1

Kim, D., Song, I., Keum, S., Lee, T., Jeong, M. J., Kim, S. S., McEnery, M. W., & Shin, H. S. (2001). Lack of the burst firing of thalamocortical relay neurons and resistance to absence seizures in mice lacking alpha(1G) T-type Ca(2+) channels. *Neuron*, *31*(1), 35–45. https://doi.org/10.1016/s0896-6273(01)00343-9

Larkum, M. E., Zhu, J. J., & Sakmann, B. (1999). A new cellular mechanism for coupling inputs arriving at different cortical layers. *Nature*, *398*(6725), 338–341. https://doi.org/10.1038/18686

La Terra, D., Bjerre, A.-S., Rosier, M., Masuda, R., Ryan, T. J., & Palmer, L. M. (2022). The role of higher-order thalamus during learning and correct performance in goal-directed behavior. *eLife*, *11*. https://doi.org/10.7554/eLife.77177

Leleo, E. G., & Segev, I. (2021). Burst control: Synaptic conditions for burst generation in cortical layer 5 pyramidal neurons. *PLoS Computational Biology*, *17*(11), e1009558. https://doi.org/10.1371/journal.pcbi.1009558

Minamisawa, G., Kwon, S. E., Chevée, M., Brown, S. P., & O'Connor, D. H. (2018). A non-canonical feedback circuit for rapid interactions between somatosensory cortices. *Cell Reports*, *23*(9), 2718–2731.e6. https://doi.org/10.1016/j.celrep.2018.04.115

O'Connor, D. H., Peron, S. P., Huber, D., & Svoboda, K. (2010). Neural activity in barrel cortex underlying vibrissa-based object localization in mice. *Neuron*, *67*(6), 1048–1061. https://doi.org/10.1016/j.neuron.2010.08.026

Payeur, A., Guerguiev, J., Zenke, F., Richards, B. A., & Naud, R. (2021). Burst-dependent

synaptic plasticity can coordinate learning in hierarchical circuits. *Nature Neuroscience*, *24*(7), 1010–1019. https://doi.org/10.1038/s41593-021-00857-x
Schultz, W. (1998). Predictive reward signal of dopamine neurons. *Journal of Neurophysiology*, *80*(1), 1–27. https://doi.org/10.1152/jn.1998.80.1.1
Varela, C. (2014). Thalamic neuromodulation and its implications for executive networks. *Frontiers in Neural Circuits*, *8*, 69. https://doi.org/10.3389/fncir.2014.00069
Yonk, A. J., Linares-García, I., Pasternak, L., Juliani, S. E., Gradwell, M. A., George, A. J., & Margolis, D. J. (2025). Role of posterior medial thalamus in the modulation of striatal circuitry and choice behavior. *eLife*, *13*(RP98563). https://doi.org/10.7554/elife.98563.3

REVIEWERS' COMMENTS

Reviewer #1 (Remarks to the Author):

The authors have addressed all my concerns. I am particularly glad that they have re-analyzed their data to separate the learning vs perception conflation. The new analyses are convincing.

Reviewer #2 (Remarks to the Author):

The authors have carefully addressed the concerns raised by me and the other reviewers. I thank them for their thoughtful responses, and I believe the manuscript has been significantly improved. However, I have one remaining point regarding the interpretation of the reduced burst bias during the degradation stage.
In their response to my comment #5, the authors acknowledge that this reduction likely reflects two factors: 1) a genuine attenuation of aperture-selective burst coding among individual neurons, and 2) a shift in the relative proportions of narrow- and wide-encoding BCNs, which become more balanced during degradation. However, this dual interpretation is not fully reflected in the Results and Discussion. The text mainly emphasizes that burst coding is “largely abolished” or “largely diminishes” during this stage, while overlooking the contribution of the balanced coexistence of narrow- and wide-encoding BCNs.
This point is relevant to the authors’ interpretation of the animals’ behavior during degradation. The authors note that mice showed “a strong tendency to lick regardless of trial type … with hit rates remaining near ceiling and correct rejection rates close to zero, consistent with poor stimulus discrimination”. This behavioral pattern suggests that, rather than simply undergoing “un-learning”, mice may have formed a new reward contingency or adopted a new “response strategy”, in which they treated both wide- and narrow-aperture stimuli as reward-predictive. This interpretation aligns well with the observed balanced coexistence of narrow- and wide-encoding BCNs during degradation.
I suggest that the authors reflect these points in the Results and/or Discussion. This clarification would help readers better interpret the data and further strengthen the manuscript.

We thank the reviewer for this final thoughtful comment. We agree that the reduced burst bias during degradation should not be interpreted solely as a loss of aperture-selective burst coding, but rather as reflecting both an attenuation of burst bias at the single-neuron level and a population-level redistribution toward more balanced proportions of narrow- and wide-encoding BCNs. We also agree that this interpretation is relevant for understanding the animals’ behavior during degradation, particularly their high licking rates to both trial types.

To address this important point, we have revised both the Results and Discussion sections. We believe these revisions better reflect the link between the neuronal and behavioral findings.

In the Results, we now state: "During degradation, this bias toward the rewarded aperture was markedly reduced. This reduction resulted both from an actual decrease in burst bias in individual BCNs and from a redistribution at the population level, in which narrow- and wide-encoding BCNs occurred in more similar proportions (Fig. 3c)."

In the Discussion, we further elaborate: "BCNs encode and track the stimulus context during different rule stages, with burst coding flexibly aligning with the currently rewarded aperture. During degradation, this rule-related burst bias is reduced, reflecting both an attenuation of aperture-selective burst coding at the single-unit level and a more balanced coexistence of narrow- and wide-encoding BCNs at the population level. This suggests that burst-related

reward representations are attenuated during degradation, while the more balanced representation of narrow- and wide-encoding BCNs may also reflect a shift toward a behavioral strategy in which both aperture stimuli are treated as reward-predictive. Consistent with this interpretation, mice licked at high rates to both trial types during degradation."

Reviewer #3 (Remarks to the Author):

The authors did a thorough revision of their paper and addressed all my concerns. I think the author have a novel and quite important core result. I support publication.
